# Genomic Analysis of the Basal Lineage Fungus *Rhizopus oryzae* Reveals a Whole-Genome Duplication

**DOI:** 10.1371/journal.pgen.1000549

**Published:** 2009-07-03

**Authors:** Li-Jun Ma, Ashraf S. Ibrahim, Christopher Skory, Manfred G. Grabherr, Gertraud Burger, Margi Butler, Marek Elias, Alexander Idnurm, B. Franz Lang, Teruo Sone, Ayumi Abe, Sarah E. Calvo, Luis M. Corrochano, Reinhard Engels, Jianmin Fu, Wilhelm Hansberg, Jung-Mi Kim, Chinnappa D. Kodira, Michael J. Koehrsen, Bo Liu, Diego Miranda-Saavedra, Sinead O'Leary, Lucila Ortiz-Castellanos, Russell Poulter, Julio Rodriguez-Romero, José Ruiz-Herrera, Yao-Qing Shen, Qiandong Zeng, James Galagan, Bruce W. Birren, Christina A. Cuomo, Brian L. Wickes

**Affiliations:** 1 The Broad Institute of MIT and Harvard, Cambridge, Massachusetts, United States of America; 2 Los Angeles Biomedical Research Institute, Harbor-UCLA Medical Center, Torrance, California, United States of America; 3 Bioproducts and Biocatalysis Research, National Center for Agricultural Utilization Research, USDA-ARS, Midwest Area, Peoria, Illinois, United States of America; 4 Department of Biochemistry, Université de Montréal, Montreal, Canada; 5 Department of Biochemistry, University of Otago, Otago, New Zealand; 6 Department of Botany, Faculty of Science, Charles University, Prague, Czech Republic; 7 Division of Cell Biology and Biophysics, School of Biological Sciences, University of Missouri, Kansas City, Missouri, United States of America; 8 Research Faculty of Agriculture, Hokkaido University, Sapporo, Japan; 9 Departamento de Genética, Universidad de Sevilla, Sevilla, Spain; 10 Department of Microbiology and Immunology, University of Texas Health Science Center at San Antonio, San Antonio, Texas, United States of America; 11 Instituto de Fisiología Celular, Universidad Nacional Autónoma de México, Mexico City, Mexico; 12 Department of Plant Biology, University of California Davis, Davis, California, United States of America; 13 Cambridge Institute for Medical Research, Cambridge, United Kingdom; 14 Departamento de Ingeniería Genética, Unidad Irapuato, Centro de Investigación y de Estudios Avanzados del IPN, Mexico City, Mexico; University of California San Francisco, United States of America

## Abstract

*Rhizopus oryzae* is the primary cause of mucormycosis, an emerging, life-threatening infection characterized by rapid angioinvasive growth with an overall mortality rate that exceeds 50%. As a representative of the paraphyletic basal group of the fungal kingdom called “zygomycetes,” *R. oryzae* is also used as a model to study fungal evolution. Here we report the genome sequence of *R. oryzae* strain 99–880, isolated from a fatal case of mucormycosis. The highly repetitive 45.3 Mb genome assembly contains abundant transposable elements (TEs), comprising approximately 20% of the genome. We predicted 13,895 protein-coding genes not overlapping TEs, many of which are paralogous gene pairs. The order and genomic arrangement of the duplicated gene pairs and their common phylogenetic origin provide evidence for an ancestral whole-genome duplication (WGD) event. The WGD resulted in the duplication of nearly all subunits of the protein complexes associated with respiratory electron transport chains, the V-ATPase, and the ubiquitin–proteasome systems. The WGD, together with recent gene duplications, resulted in the expansion of multiple gene families related to cell growth and signal transduction, as well as secreted aspartic protease and subtilase protein families, which are known fungal virulence factors. The duplication of the ergosterol biosynthetic pathway, especially the major azole target, lanosterol 14α-demethylase (*ERG11*), could contribute to the variable responses of *R. oryzae* to different azole drugs, including voriconazole and posaconazole. Expanded families of cell-wall synthesis enzymes, essential for fungal cell integrity but absent in mammalian hosts, reveal potential targets for novel and *R. oryzae*-specific diagnostic and therapeutic treatments.

## Author Summary

*Rhizopus oryzae* is a widely dispersed fungus that can cause fatal infections in people with suppressed immune systems, especially diabetics or organ transplant recipients. Antibiotic therapy alone is rarely curative, particularly in patients with disseminated infection. We sequenced the genome of a pathogenic *R. oryzae* strain and found evidence that the entire genome had been duplicated at some point in its evolution and retained two copies of three extremely sophisticated systems involved in energy generation and utilization. The ancient whole-genome duplication, together with recent gene duplications, has led to the expansion of gene families related to pathogen virulence, fungal-specific cell wall synthesis, and signal transduction, which may contribute to the aggressive and frequently life-threatening growth of this organism. We also identified cell wall synthesis enzymes, essential for fungal cell integrity but absent in mammals, which may present potential targets for developing novel diagnostic and therapeutic treatments. *R. oryzae* represents the first sequenced fungus from the early lineages of the fungal phylogenetic tree, and thus the genome sequence sheds light on the evolution of the entire fungal kingdom.

## Introduction

**Figure 1:**
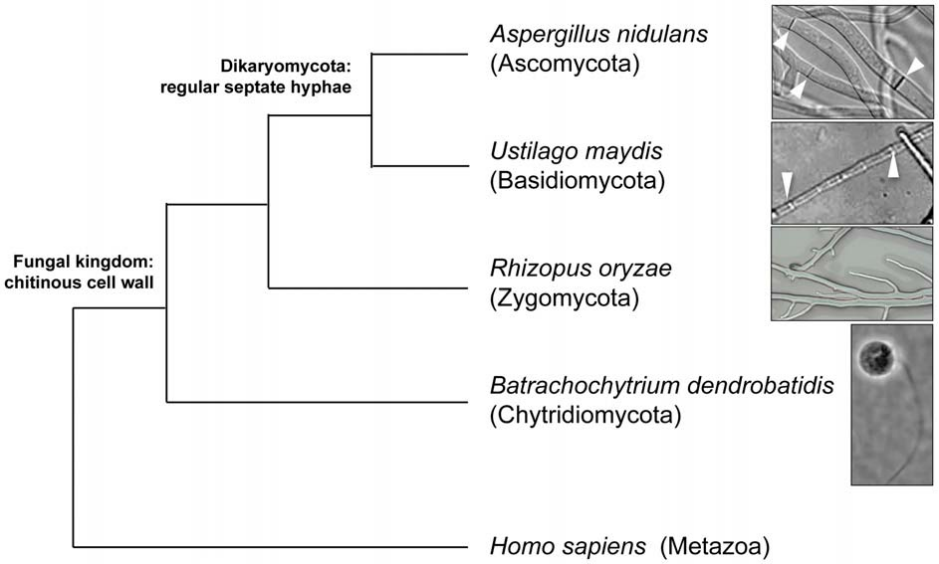
Relationship of major phyla within the fungal kingdom. Phylogeny is shown as a dendrogram using *H. sapiens* (Metazoa) as the out-group. *B. dendrobatidis* (phylum Chytridiomycota) is a unicellular organism with flagellated spores. The terrestrial multicellular fungi include the monophyletic Dikaryomycota (Ascomycota and Basidiomycota) and the more basal fungal lineages, including *R. oryzae*. In contrast to the Dikaryomycota fungi that form hyphae divided by septa (white arrows), the hyphae of *R. oryzae* are multinucleate but not divided into separate cells (coenocytic).

The fungal kingdom comprises an estimated 1.5 million diverse members spanning over 1 billion years of evolutionary history. Within the fungal kingdom, four major groups (“Phyla”)—the Chytridiomycota, Zygomycota, Ascomycota and Basidiomycota— are traditionally recognized [1],[2] (Figure 1). Recent phylogenetic studies confirm a monophyletic group (the Dikarya) that includes the ascomycetes and basidiomycetes, and proposed polyphyletic states for the two basal lineages of chytridiomycetes and zygomycetes [3]. The majority of fungal genomic resources generated thus far are for the Dikarya (http://www.ncbi.nlm.nih.gov/genomes/leuks.cgi) and typically focused on fungi that are pathogenic. However, many members of the basal lineages also are important pathogens [4],[5] while others serve as outstanding models for understanding the evolution of the entire fungal kingdom. This study reports the analysis of the genome sequence of *Rhizopus oryzae*, which represents the first fungus sequenced from the polyphyletic basal lineages described as the zygomycetes [3].

*R. oryzae* is a fast growing, filamentous fungus and is by far the most common organism isolated from patients with mucormycosis, a highly destructive and lethal infection in immunocompromised hosts [4],[5]. Approximately 60% of all disease manifestation and 90% of all rhinocerebral cases are caused by *R. oryzae* [6]. The rapid growth rate and the angioinvasive nature of the disease leads to an overall mortality of >50% [7]. In the absence of surgical removal of the infected focus, antifungal therapy alone is rarely curative, resulting in 100% mortality rate for patients with disseminated disease [8].

The genus *Rhizopus* was first described in 1821 by Ehrenberg and belongs to the order Mucorales in the phylum Zygomycota [9]. Unlike the Dikarya, fungal species belonging to this basal lineage are characterized, in part, by aseptate hyphae. If septa are produced, they occur only between the junctions of reproductive organs and mycelium, or occasionally between aged mycelia. As a saprobe, *Rhizopus* is ubiquitous in nature and a number of species in the genus are used in industry for food fermentation (e.g., tempeh, ragi), production of hydrolytic enzymes, and manufacture of the fermentation products lactic acid and fumaric acid [10].

There are taxonomic complications within the *Rhizopus* genus, including the recently proposed reclassification of *R. oryzae* (previous synonym *R. arrhizus*) to include two species, *R. oryzae* and *R. delemar* [11]. According to this new nomenclature, the sequenced strain 99–880 would be reclassified as *R. delemar*, but will be referred to as *R. oryzae* in this study in an effort to minimize confusion until this nomenclature is widely accepted.

Analysis of the *R. oryzae* genome provides multiple lines of evidence to support an ancient whole-genome duplication (WGD), which has resulted in the duplication of all protein complexes that constitute the respiratory electron transport chain, the V-ATPase, and the ubiquitin–proteasome system. The ancient WGD, together with recent gene duplications, have led to the expansion (2- to 10-fold increase) of gene families related to pathogen virulence, fungal-specific cell wall synthesis, and signal transduction, providing *R. oryzae* the genetic plasticity that could allow rapid adaptation to adverse environmental conditions, including host immune responses.

## Results/Discussion

### Genome sequencing and organization

*Rhizopus oryzae* strain 99–880, isolated from a fatal case of mucormycosis, was chosen for whole genome sequencing. The whole genome shotgun reads were generated using Sanger sequencing technology (Materials and Methods, Table S1). The genome assembly consists of 389 sequence contigs with a total length of 45.3 Mb and an N_50_ contig length of 303.7 kilobases (kb) (that is, 50% of all bases are contained in contigs of at least 303.7 kb). Over 11-fold sequence coverage provides high base accuracy within the consensus sequence, with more than 99.5% of the sequence having quality scores of at least 40 (1 error every 10^4^ bases) (Table 1).

**Table 1:** *Rhizopus oryzae* genome statistics.

**Assembly statistics**	
Total contig length (Mb)	45.26
Total scaffold length (Mb)	46.09
Average base coverage (Fold)	11
N_50_ contig (kb)	303.66
N_50_ scaffold (Mb)	3.1
Linkage groups	15
GC-content (%)	35.6
Coding (%)	40.6
Non-coding (%)	32.6
**Coding sequence**	
Percent coding (%)	39.0
Average gene size (bp)	1212
Average gene density (kb/gene)	2.6
Protein-coding genes	17,467
Exons	57,981
Average exon size (bases)	310
Exons/gene	3.3
tRNA genes	239
**Non-coding sequence**	
Introns	40,514
Introns/gene	2.32
Average intron length (base)	79
Intergenic regions	17,546
Average intergenic distance (bp)	1420

**Figure 2:**
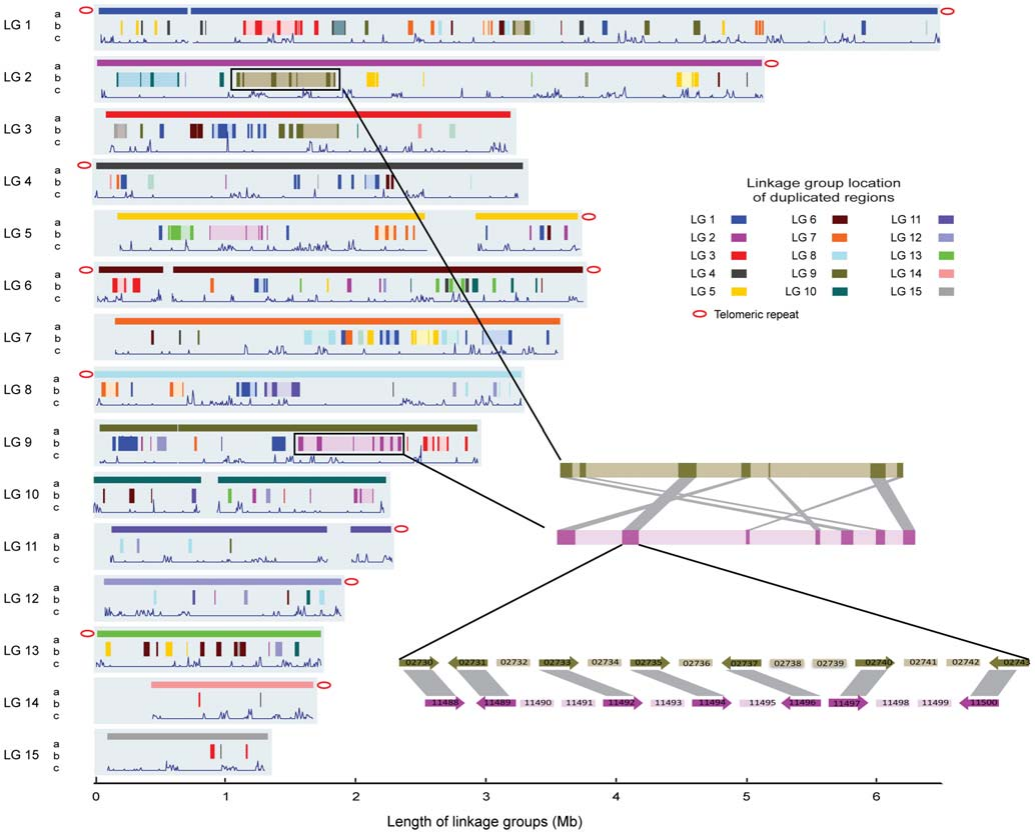
*R. oryzae* genomic structure showing duplicated regions retained after WGD and distribution of LTR transposable elements. The length of the light blue background for each linkage group is defined by the optical map. For each chromosome, row a represents the genomic scaffolds positioned on the optical linkage groups. The red oval indicates linkage to telomeric repeat arrays. Row b displays the 256 duplicated regions capturing 648 gene pairs and spanning 12% of the genome. The shaded backgrounds around some duplicated regions illustrate the duplicated blocks by merging duplicated regions that are within 200 kb after discounting the transposon sequences. These extended duplicated blocks contain the same amount of the duplicates but span 23% of the genome. A pair of corresponding duplicated regions between linkage 2 and linkage 9 are shown in the zoomed images. The numbers in the gene boxes are gene IDs. Row c corresponds to the distribution of the LTR retroelements.

An *R. oryzae* optical map of 52-fold physical coverage, consisting of 15 linkage groups, was constructed to anchor the assembly and to generate a physical map. The 22 largest scaffolds (44 Mb), corresponding to over 96% of the assembled bases, cover 95% of the optical map (Materials and Methods, Table S2), reflecting the long-range continuity of the assembly and near complete genome coverage. The remaining 5% of the optical map falls into gaps in the assembly or within the highly repetitive ends of linkage groups. We also linked reads containing telomeric tandem repeats (CCACAA)_n_ to 12 of the 30 linkage group ends, confirming that the assembly extends close to telomeric repeats (Materials and Methods, Figure 2).

### Repeat and transposable elements

The *R. oryzae* genome is highly repetitive compared with other fungal genomes (Materials and Methods, Table S3). Over 9 Mb of sequence, accounting for 20% of the assembly, consists of identifiable transposable elements (TEs) (Materials and Methods, Table 2). These include full-length and highly similar copies of many diverse types of TEs from both Class I (retrotransposon) and Class II (DNA transposon) elements. The active transcription of some TEs is supported by the identification of corresponding expressed sequence tags (ESTs) (Materials and Methods, Table 2 and Table S4), suggesting that these elements may be currently active. The Ty3/gypsy-like long terminal repeat (LTR) retrotransposons are the most abundant type of TEs, accounting for 8% of the assembly. The overall distribution of these LTR elements exhibits strong insertion-site preference, often co-localizing with tRNA genes (Figure S1).

**Table 2:** Transposable elements (TEs) in the *R. oryzae* genome.

Elements	Total bases^a^	% of assembly	Sequence identity (%)^b^	EST^c^
**Class I transposons**	**5,589,511**	**12.13**		
LTR elements / Ty3	3,700,795	8.03	97%	Yes
LINES	1,742,093	3.78	97%	Yes
DIRS	146,622	0.32	97%	Yes
**Class II transposons**	**3,462,307**	**7.50**		
Mariners	1,666,728	3.62	98%	Yes
En/Spn	314,481	0.68	98%	No
Tigger	262,307	0.57	94%	No
Crypton	191,823	0.42	98%	No
Helitron	66,534	0.14	99%	No
**Total**	**9,051,818**	**19.63**		

a The genomic distribution of the representative elements was identified using the sensitive mode of RepeatMasker version open-3.0.8, with cross_match version 0.990329.

b Sequence identity was computed based on the average identity of the full-length copies of each representative against the consensus sequence of each group.

c EST reads overlap with the identified TEs (see Table S6).

### Genome annotation and evidence for a whole-genome duplication

A total of 17,467 annotated protein-coding genes, including 13,895 genes not overlapping TEs, were predicted in the *R. oryzae* genome (Materials and Methods, Table 1). About 45% of the non-TE proteins have paralogs within the genome and are grouped into 1,870 multi-gene families. Moreover, 17% of these paralogous genes are grouped into two-member gene families, more than two-fold higher than any other representative fungal genome (Materials and Methods, Figure S2). This high proportion of duplicated gene pairs prompted an investigation into whether multiple segmental duplications or an ancestral whole-genome duplication (WGD) event occurred in *R. oryzae*.

WGD was first proposed in *Saccharomyces cerevisiae* based on the order and orientation of duplicated genes in the corresponding chromosomes [12]. This was further confirmed by comparison to a related, non-duplicated species that identified a signature of 457 duplicated gene pairs interleaved with asymmetric gene loss in duplicated regions [13],[14]. In the *R. oryzae* genome, we identified 648 paralogous gene pairs, which can be uniquely grouped into 256 duplicated regions containing at least three, and up to nine, duplicated genes (Materials and Methods, Figure S3, and Table S5, S6). Together the duplicated regions cover approximately 12% of the genome and span all 15 linkage groups (Figure 2 and Table S5). The duplicated genes in each of these regions are found in the same order and orientation, providing evidence of an ancestral duplicated state for these regions.

In addition to the similarities of the signature of WGD found in *S. cerevisiae*, we observed multiple lines of evidence to support WGD to the exclusion of independent duplications. First, if the 256 duplicated regions in *R. oryzae* are the cumulative result of multiple segmental duplications, some of the early duplicated regions should also be part of later duplication events. Such regions would be present in the genome as triplets. We estimate that the probability of segments being duplicated two or more times approaches a Poisson distribution, in which 47 triplets would be expected within the 256 duplicated segments. However, we only detected three potential triplet regions (*p*<10^−16^) (Materials and Methods, Table S5), which refutes the model of multiple segmental duplications. Second, we observed a clear correlation between the presence of TEs and breakpoints within duplicated regions, allowing us to extend the initial duplicated regions in the same orientation into larger blocks that span 23% of the genome (Materials and Methods, Figure 2).

**Figure 3:**
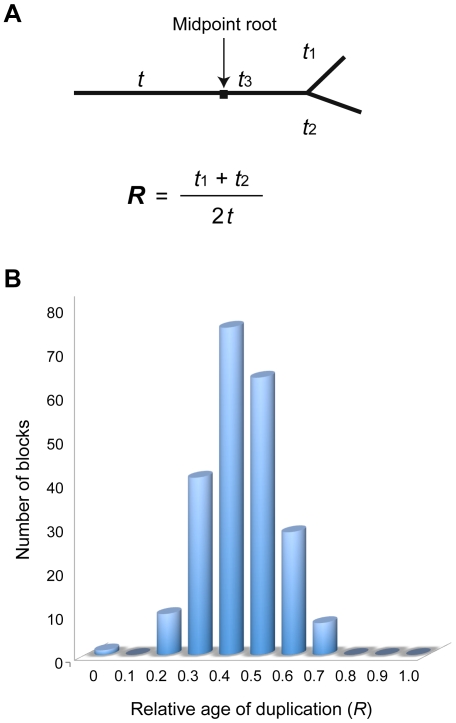
Estimation of duplication dates using *P. blakesleeanus* as an outgroup. (A) An unrooted tree diagram for the duplicated gene pairs in *R. oryzae* and their homologous gene in *P. blakesleeanus*. Midpoint rooting is used to calculate of the relative age of each duplication (*R*) in relation to the root. The branch lengths as substitutions per site for the unrooted tree topology were calculated using the WAG evolutionary model [49] employing a maximum likelihood-based package, PhyML [50]. The distance between two duplicated genes in *R. oryzae* is *t*_1_+*t*_2_, and the distances between the duplicates and their orthologous gene in *P. blaskesleeanus* are *t*+*t*_3_+*t*_1_ and *t*+*t*_3_+*t*_2_, respectively. (B) The distribution of the relative duplication time for each duplicated region in comparison to the root (*R*). *R* is normalized within each duplicated region by averaging the divergences of all the duplicated gene pairs within the region. If the divergence time between *R. oryzae* and *P. blakesleeanus* is defined as *t* using midpoint rooting, approximately 78% of all these regions were estimated to be duplicated within one standard deviation (0.115) of the mean (0.386*t*).

The comparison of protein sets of *R. oryzae* and *Phycomyces blakesleeanus*, a distantly related fungus in the order Mucorales that has been recently sequenced at the Joint Genome Institute (http://genome.jgi-psf.org/Phybl1/Phybl1.home.html), further strengthens the WGD argument. A significant excess of gene duplicates is observed in the *R. oryzae* genome compared with *P. blakesleeanus* (*p*<10^−16^) (Materials and Methods, Table S7). Out of the 648 paralogous gene pairs retained in the syntenic regions, 507 share homologs in *P. blakesleeanus* genome. More than 84% (426) of these homologous genes pairs match a single *P. blakesleeanus* gene, reflecting a 2-to-1 correspondence (*p*<10^−150^). We further estimated the relative duplication time for each duplicated region by averaging the divergences of all the duplicated gene pairs within the region (Figure 3). If the divergence time between *R. oryzae* and *P. blakesleeanus* is defined as *t* using midpoint rooting (Figure 3A), approximately 78% of all these regions were estimated to be duplicated within one standard deviation (0.115) of the mean (0.386*t*), arguing strongly for a single origin for these duplicated regions (Figure 3B).

Based on the above observations, we conclude that the modern genome of *R. oryzae* arose by a WGD event, followed by massive gene loss. This event resulted in a net gain of at least 648 genes compared to the pre-duplication ancestor. The gene pairs retained after WGD are significantly enriched for protein complexes involved in various metabolic processes (Materials and Methods, Table S8). In particular, we observed the duplication of all protein complexes that constitute the respiratory electron transport chain, the V-ATPase, and the ubiquitin–proteasome systems (Table 3 and Table S9, S10, S11). These protein complexes contain more than 100 protein subunits in total, of which about 80% were retained as duplicates after WGD, including every core subunit of all three complexes. Because an imbalance in the concentration of the subcomponents of large protein–protein complexes can be deleterious [15], duplication of entire complexes should be difficult to achieve by independent duplication events. This observation provides an additional line of evidence to support an ancient WGD in *R. oryzae*.

**Table 3:** Duplication of protein complexes in the *R. oryzae* genome*.

Complexes	Respiratory chain complexes						V-ATPase			Ubiquitin–proteasome system					
Subunits	I	II	III	IV	ATPase	Total	V_1_	V_0_	Total	Alpha	Beta	ATPase	LID	Modifier	Total
Reference genes	28	4	9	9	10	60	7	5	12	7	7	6	13	3	36
*R. oryzae* duplicates	20	3	8	8	8	47	5	3	8	6	6	5	10	2	29
% duplicated genes	71.4	75.0	88.9	88.9	80.0	78.3	71.4	60.0	66.7	85.7	85.7	83.3	76.9	66.7	80.6

*: Duplicated protein complexes in *R. oryzae* retained after WGD. The reference nuclear genes of protein complexes from *Saccharomyces cerevisiae* or *Neurospora crassa* were used to identify homologous sequences in the *R. oryzae* proteome. We searched for homologous genes using BLASTP (1e–5) and manually checked for short proteins that usually have higher e-values.

Large-scale differences exist among the duplicated genes in the post-WGD genomes of *S. cerevisiae* and *R. oryzae*. The increased copy number of some glycolytic genes in *S. cerevisiae* may have conferred a selective advantage in adapting to glucose-rich environments through rapid glucose fermentation [16]. The retention of duplicated protein complexes involved in energy generation in *R. oryzae* could have provided an advantage related to the rapid growth of this organism. About 16% of the *R. oryzae* duplicates are also retained in *S. cerevisiae* (BLASTP 1e-5). The genes retained in both systems are enriched for kinases and proteins involved in signal transduction (21%), and proteins involved in transcription/translation processes (21%) (Table S12), possibly indicating potential selective advantage for these genes in both fungal species. Among these shared gene pairs, three out of the four that show accelerated evolution encode enzymatic activities, such as hydrolase, ligase, and protease activities (Table S12).

### Gene family expansions

Compared to the genomes of sequenced dikaryotic fungi, several gene families are significantly expanded in *R. oryzae*, including the superclass of P-loop GTPases and their regulators, and the gene families that are essential for protein hydrolytic activities and cell wall synthesis (Materials and Methods, Table 4, and Tables S13, S14, S15, S16).

**Table 4:** Gene family expansion in the *R. oryzae* genome.

Species	Cell wall synthesis		Protein hydrolysis		Cell signaling	
	CHS	CDA	SAP	Subtilases	GTPases	GTPase regulators
***Rhizopus oryzae***	**23**	**34**	**28**	**23**	**184**	**246**
*Aspergillus fumigatus*	9	9	6	4	81	76
*Neurospora crassa*	7	5	17	8	84	79
*Magnaporthe grisea*	8	11	8	7	—	—
*Saccharomyces cerevisiae*	7*	2*	7	4	82	76
*Candida albicans*	8*	1*	14	2	—	—
*Cryptococcus neoformans*	8	4	7	2	78	77
*Coprinus cinereus*	9	16	2	3	86	83
*Ustilago maydis*	8	8	6	1	80	77

Expanded gene families in *R. oryzae* compared to selected dikaryotic fungal genomes.

—, not tested.

*: based on the SGD (http://www.yeastgenome.org/) and CGD (http://www.candidagenome.org/) annotation.

#### Expansion of P-loop GTPases and their regulators

To assess the complexity of the basic cellular processes in *R. oryzae*, including proteosynthesis, membrane trafficking, cytoskeletal dynamics, signalling, or cell division, we analyzed in detail a diverse group of proteins central for these processes —the superclass of P-loop GTPases (Table S13) and their regulators (Tables S14). Overall, the general structures of the distinct types of GTPase superclasses and their regulators are very similar in *R. oryzae* compared to dikaryotic fungi. However, a large proportion of these genes have multiple paralogs in *R. oryzae* resulting from gene retention after WGD and additional duplications (Materials and Methods, Table S13). Therefore, the total number of GTPases and their regulators in *R. oryzae* exceeds more than twice and three times, respectively, the number of genes in the other genomes analyzed (Table 4). As the molecular switches that mediate regulatory and signaling steps in diverse cellular processes [17], such an increase might provide the organism an enhanced capacity for coordinating growth and metabolism under highly varied environmental conditions.

#### Expansion of secreted proteases

The expansion of protease gene families in *R. oryzae* suggests an increased ability of *R. oryzae* to degrade organic matter (Materials and Methods, Table S15) and is consistent with its centuries-old use in fermentation and production of hydrolytic enzymes [10]. The most noteworthy expansions among the protease gene families are of secreted aspartic proteases (SAP) and subtilases (Table 4), which constitute important virulence factors in many pathogenic fungi [18],[19]. The large family of *R. oryzae* SAP proteins includes three pairs of genes retained after WGD and three pairs of nearly identical, tandem duplicates that likely arose from recent duplications (Figure S4). The expansion of proteolytic enzymes in *R. oryzae* may facilitate hyphal penetration through decaying organic materials or after establishment of infection through tissues and vessels. Extracellular proteolytic activity of both SAP and subtilase proteins has been linked to virulence in pathogenic *Rhizopus* isolates [20],[21], suggesting the potential utility of this group of proteins in vaccine or drug development.

#### Expansion of fungal cell wall synthesis enzymes

**Figure 4:**
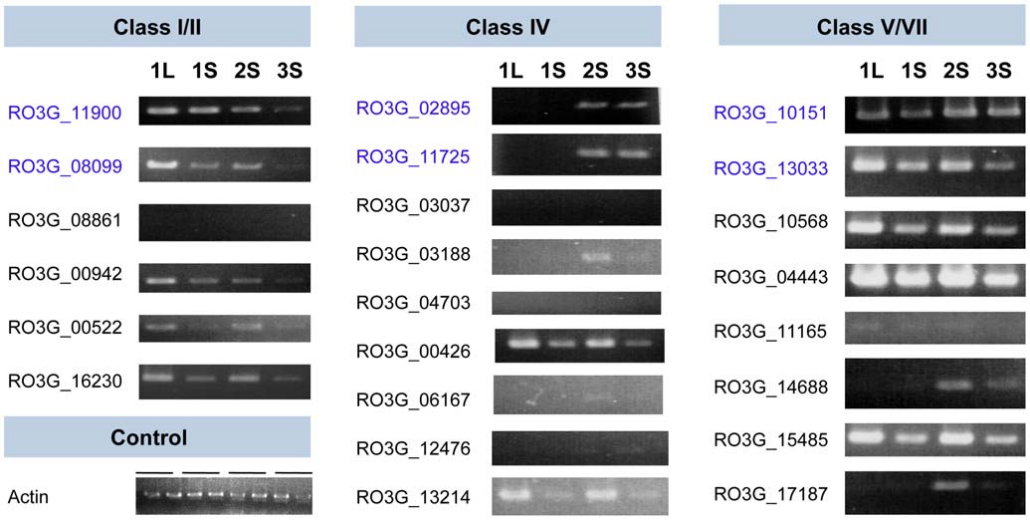
RT–PCR of *R. oryzae* chitin synthases (CHSs). Presence of a transcript was detected from mycelia grown with four different growth phases: 1L, 1-day-old liquid culture; 1S, 1-day-old agar plate; 2S, 2-day-old agar plate; and 3S, 3-day-old agar plate. Gene pairs retained after WGD as detected in the duplicated regions are shown in blue.

Another important expansion in *R. oryzae* includes gene families that are essential for the biosynthesis of the fungal cell wall, a defining cellular structure that provides physical support and osmotic integrity. Unlike dikaryotic fungi, the cell wall of *R. oryzae* and other Mucorales contains a high percentage of chitin and chitosan, which are synthesized by chitin synthases (CHS) and chitin deacetylases (CDA), respectively [22],[23]. The *R. oryzae* CHS and CDA gene families have expanded to 23 and 34 genes, respectively, more than double the numbers observed in any sequenced dikaryotic fungus (Table 4). These families include three pairs of CHS and four pairs of CDA retained after WGD. RT-PCR amplification of the CHS catalytic domains demonstrated that 20 of the 23 CHS, including all the duplicates, are transcribed, suggesting their potential functional roles (Materials and Methods, and Figure 4). Cell wall localization is predicted for 14 of the 34 identified CDA genes based on potential glycosylphosphatidylinositol (GPI)-modification sites (Materials and Methods, Table S16). The surface accessibility of these proteins suggests that they could serve as targets for reliable diagnosis of this invasive pathogen.

#### Ergosterol pathway

The ergosterol biosynthesis pathway is conserved in the *R. oryzae* genome. As a major constituent of the fungal plasma membrane [24], this fungal-specific biosynthetic pathway has been the subject of intensive investigation as a target of antifungal drugs [25]. The conservation of the entire pathway indicates that azoles, a group of drugs that specifically target this pathway [26],[27], could be used to treat *R. oryzae* infections. However, about half the genes involved in ergosterol biosynthesis, including the major azole target, lanosterol 14α-demethylase (*ERG11*, RO3G_11790, RO3G_16595), are present in multiple copies (Table S17). Acquisition of azole resistance in a clinical strain of *Candida albicans* reflected amplification of *ERG11* in a gene copy-dependent manner [28],[29]. Although experimental validation is pending, the copy number increase and divergence of duplicated protein sequences could contribute to the observed variable responses of *R. oryzae* to different azole drugs, including voriconazole and posaconazole [26],[27].

In contrast to the expansions described above, some cell wall synthesis-related genes are underrepresented in the *R. oryzae* genome. For instance, no gene encoding a putative α-1,3-glucan synthase was detected. Compared to four and three copies of β-1,3-glucan synthase (GS) reported in *S. pombe* and *S. cerevisiae*, respectively, the *R. oryzae* genome only contains two GS genes. Nevertheless, the presence of GS underlies the susceptibility of *R. oryzae* to caspofungin acetate, an antifungal agent that inhibits GS [30].

### Iron uptake and pathogenicity

Iron is required by virtually all microbial pathogens for growth and virulence [31], and sequestration of serum iron is a major host defense mechanism against *R. oryzae* infection [32]. Genomic analysis reveals that *R. oryzae* lacks genes for non-ribosomal peptide synthetases (NRPSs), the enzymes that produce the most common siderophores (hydroxamate siderophores) used by other microbes to acquire iron. Instead, *R. oryzae* relies solely on Rhizoferrin, which is ineffective in acquiring serum-bound iron [33], and therefore is heavily dependent on free iron for pathogenic growth. This explains why some patients with elevated levels of available free iron, including diabetics, are uniquely susceptible to infection by *R. oryzae* [34]. At the same time, we observed duplication of heme oxygenase (*CaHMX1*) (RO3G_07326 and RO3G_13316), the enzyme required for iron assimilation from hemin in *C. albicans* [35]. Since free iron is usually present at very low concentrations in human blood, the two copies of the heme oxygenase gene may increase iron uptake from host hemoglobin, which would be important for angioinvasive growth. The critical role of iron uptake during *R. oryzae* early infection further reinforces the strategy of treating infections as early as possible with iron chelators that cannot be utilized by *R. oryzae* as a source of iron [36].

### Insight into eukaryote evolution

As the first sequenced representative of a fungal lineage basal to the Dikarya, *R. oryzae* provides a novel vantage point for studying fungal and eukaryotic genome evolution. The *R. oryzae* genome shares a higher number of ancestral genes with metazoan genomes than dikaryotic fungi (*p*<0.00001) (Materials and Methods, Table S18). The homologs shared exclusively between *R. oryzae* and Metazoa include genes involved in transcriptional regulation, signal transduction and multicellular organism developmental processes (Figure S5). For example, in contrast to dikaryotic fungi, the *R. oryzae* genome encodes orthologs of the metazoan GTPases Rab32, the Ras-like GTPase Ral, as well as the potential positive regulators of these GTPases (Table S13, S14, Figure S6). The presence of these orthologs suggests that *R. oryzae* might share these metazoan regulatory modules, which are involved in protein trafficking, GTP-dependent exocytosis, and Ras-mediated tumorigenesis [37],[38]. In this respect, *R. oryzae* could serve as a model system for studying aspects of eukaryotic biology that cannot be addressed in dikaryotic fungi.

The genome sequence also sheds light on the evolution of multicellularity. As in other Mucorales species, *R. oryzae* hyphae are coenocytic (Figure 1), meaning that the multinucleated cytoplasm is not divided into separate cells by septa after mitosis. Our analysis suggests that the coenocytic hyphal structure of *R. oryzae* may be attributed to the absence of a functional septation initiation network (SIN), which activates actomyosin ring contraction and the formation of septa upon completion of mitosis [39]. The core components of the SIN pathway, as described in *S. pombe*, and the homologous mitotic exit network (MEN) in *S. cerevisiae*, are common to both fission and budding yeasts (Table S19), including the protein kinases Sid2 (Dbf2p/Dbf20p) and Cdc7 (Cdc15p). Our kinome analysis revealed that *R. oryzae* lacks the Sid2 ortholog. Even though the fungus possesses five copies of Cdc7 homologs, the proteins lack the characteristic C-terminal tail (Figure S7, Table S19). The chytrid fungus *Batrachochytrium dendrobatidis*, fruitfly *Drosophila melanogaster* and nematode *Caenorhabditis elegans* all lack Cdc7 orthologs. This omission suggests that Cdc7 in dikaryotic fungi may have acquired the C-terminal extension, which contributes a significant role in cytokinesis, after the divergence of the lineage leading to *Rhizopus*. Although homologous genes of these two kinase families are also reported in plants and metazoa, their functions are diverged from coordinating the termination of cell division with cytokinesis [40],[41]. We therefore hypothesize that the fungal septation pathway may have arisen in the dikaryotic lineage specifically and the multinucleate *R. oryzae* cellular organization may reflect a primitive developmental stage of multicellularity, supporting the theory that multicellularity evolved independently in metazoan, plant, and fungal lineages [42].

### Conclusions

Gene duplication plays an important role in genome evolution, thus whole genome duplication (WGD) is expected to have a large impact on the evolution of lineages in which it has occurred [43]. The post-WGD retention of entire protein complexes and gene family expansions could enable *R. oryzae* to rapidly use more complex carbohydrates for energy sources and quickly accommodate major environmental changes. This outcome of WGD may underlie its aggressive disease development observed clinically and its rapid growth rate observed experimentally (Materials and Methods, Table S20).

Due to the lack of suitable laboratory tests, the diagnosis of mucormycosis is notoriously difficult [6]. As an acute and rapidly fatal infection, delayed diagnosis has been associated with a dramatically worse outcome, thus a timely and accurate diagnostic assay is essential for earlier treatment [44]. Our analysis illustrates the value of the *R. oryzae* genome sequence in understanding the basis of angioinvasive pathogenicity and suggests ways to improve diagnosis and treatment. The *R. oryzae* specific cell wall glycoproteins (e.g., the chitin deacetylases) identified through this analysis could serve as targets for reliable diagnosis of this invasive pathogen and therefore could have a profound impact controlling the *R. oryzae* infection.

The *R. oryzae* genome also provides the first glimpse into the genome structure and dynamics of a basal fungal lineage, demonstrating the novel perspective of this model organism for the study of eukaryotic biology that cannot be addressed in dikaryotic fungi. Importantly, *R. oryzae* gene function can be experimentally studied using transformation [45]. Ongoing sequencing projects for other basal fungi, including two other Mucorales species and at least three chytrids, will further our understanding of the evolution of the fungal kingdom. In addition, the *R. oryzae* sequence also reveals an important observation about the evolution of multicellular eukaryotes, with *R. oryzae* representing a preliminary step toward multicellularity, a trait that evolved multiple times in the history of the different eukaryotic lineages.

## Materials and Methods

### Sequencing and assembly

Sanger sequencing technology was employed for the *R. oryzae* genome. The sequence was generated using three whole-genome shotgun libraries, including two plasmid libraries containing inserts averaging 4 kb and 10 kb, and a Fosmid library with 40-kb inserts (Table S1), then assembled using Arachne [46].

### Optical map

The *R. oryzae* optical map was constructed using restriction enzyme *Bsu*36I [47]. The correspondences of the restriction enzyme cutting sites and the lengths of assembly fragments based on *in silico* restriction were used to order and orient the scaffolds of the assembly to the map (Table S2).

### Telomeres

Telomeric tandem repeats (CCACAA)*_n_* of at least 24 bases were identified in the unplaced reads and linked to scaffolds based on read pair information.

### Repetitive elements

Repeat sequences were detected by searching the genome sequence against itself using CrossMatch (http://www.genome.washington.edu/UWGC/analysistools/Swat.cfm) and filtering for alignments longer than 200 bp with greater than 60% sequence similarity (Table S3).

### Transposable elements (TEs)

The full-length LTR retrotransposons were identified using the LTR_STRUCT program [48]. The DDE DNA transposons were identified using EMBOSS einverted (http://emboss.sourceforge.net/) to locate the inverted repeats, in addition to a BLAST search for the transposase. The LINE elements, DIRS-like elements, Cryptons and Helitrons from *R. oryzae* were detected in a series of TBLASTN searches of the *R. oryzae* sequence database, using the protein sequences as queries. The genomic distribution of the representative elements was identified using the sensitive mode of RepeatMasker version open-3.0.8, with cross_match version 0.990329 (Figure S1).

### Gene annotation and gene families

Protein-encoding genes were annotated using a combination of 864 manually curated genes, based on over 16,000 EST BLAST alignments and *ab initio* gene predictions of FGENESH, FGENESH+ and GENEID. Multigene families were constructed by searching each gene against every other gene using BLASTP, requiring matches with E≤10^−5^ over 60% of the longer gene length (Figure S2).

### Identification of duplicated regions

A duplicated region was defined as two genomic regions that contain at least three pairs of genes in the same order and orientation. The best BLAST hits (2754 gene pairs, among non-TE proteins) with a threshold value of E≤10^−20^ were used to search for such duplicated regions. Varying the distance between neighboring gene pairs from 10 kb to 50 kb did not significantly affect the amount of detected duplications (Table S5). We did not find duplicated regions among sets of genes with randomized locations (1000 permutation tests), attesting to the statistical significance of the duplicated regions detected through this analysis (Figure S3).

If the observed duplicated regions were created through sequential segmental duplications, the duplicated segments will follow a Poisson distribution in the genome.
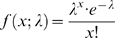
where: *e* = 2.71828;

*x* is the probability of which is given by the function; and

λ is a positive real number, equal to the expected number of occurrences that occur during the given interval.

When *f*(*x*; 1) = 100; *f*(*x*; 2) = 18.4, *f*(*x*; 3) = 6.13;

That is, for every 100 duplicates, we expect 18.4 triplications. Thus, for the 256 duplicated regions observed in the *R. oryzae* genome, the expected number of triplications would be 47; however, we only detected three. The probability for this observation is:
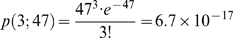



### Triplets

All the genes within the duplicated regions, including the non-paralogous genes, were used to compute multiple correspondences with other duplicated regions (Table S8). At a 10-kb distance between neighboring paralogs, we observed 174 duplicated regions, but no triplets, although the expected number of triplets is 32 if duplications were created through sequential segmental duplications. At a 20-kb distance, we only detected three potential triplet regions (Table S5).

### Comparative proteomics between *R. oryzae* to *Phycomyces blakesleeanus*

Reciprocal BLAST searches between *P. blakesleeanus* and *R. oryzae* protein sets were conducted using BLASTP, requiring matches with E≤10^−20^ over 60% of the query gene length (Table S7). For 852 duplicated genes (426 genes pairs) in *R. oryzae*, and their corresponding homologous gene in the *P. blakesleeanus* genome, we constructed unrooted trees (Figure 3A) using PhyML [49]. The mean distance of each gene pair among three homologous genes were calculated using the WAG evolutionary model [50], where the distance between two duplicated genes in *R. oryzae* is *t*_1_+*t*_2_, and the distances between the duplicates and their orthologous gene in *P. blakesleeanus* are *t*+*t*_3_+*t*_1_ and *t*+*t*_3_+*t*_2_, respectively. The relative duplication time of each duplicated region in comparison to the root is calculated as an average duplication time (*R* = ½ (*t*_1_ *+t*_2_)/*t*) of all the gene pairs within the region (Figure 3).

### Functional enrichment and conservation of retained genes

The non-TE genes were assigned functional annotation using the program Blast2GO [51] (BLAST cut-off = 1e–20). GO term enrichments in the duplicated gene set were determined using Fisher's exact test [52] (Table S8).

### Characterization of protein complexes, protein families, and ergosterol pathway

The characterized MRC complex I of *Neurospora crassa* [53] and all other complexes from *Saccharomyces cerevisiae* based on the SGD annotation (http://www.yeastgenome.org/) were used as reference sets to search homologous sequences in the *R. oryzae* proteome (Table S9, S10, S11, S17).

### Comparison of P-loop GTPases and their regulators

The GTPases were identified by BLAST and PSI-BLAST searches of the database of predicted *R. oryzae* proteins and the nr database at NCBI using query sequences of major groups of P-loop GTPases and regulators of the Ras superfamily of GTPases culled from the literature. In addition, for identification of proteins containing poorly conserved regulatory domains, HMMER searches were used with HMM profiles built from multiple alignments retrieved from Pfam (http://www.sanger.ac.uk/Software/Pfam/) or SMART (http://smart.embl-heidelberg.de/) collections. Assignment of mutual orthologs is based mainly on reciprocal BLAST (accession numbers of individual GTPases from dikaryotic fungal genomes are available upon request) (Table S13, S14).

### Characterization of protein families

Proteolytic enzymes were annotated using HMMER as well as BLAST hits to the Merops peptidase database http://merops.sanger.ac.uk/index.htm; protein numbers from other fungi were downloaded from Merops. BLAST and HMMER (http://hmmer.janelia.org) searches and manual curation were applied to characterize gene families of CHS and CDA (Tables 15). Identification of proteins of probable exocellular locations was determined using Psort algorithms (http://psort.nibb.ac.jp/form2.html) and the presence of a signal peptide (http://www.cbs.dtu.dk/services/SignalP/). The ORFs containing a putative extracellular location and signal peptide were further analyzed for the presence of high levels of serine/threonine residues and high levels of glycosylation using the program at http://us.expasy.org/tools/scanprosite/. The presence of a GPI motif was analyzed with the algorithm located at http://mendel.imp.univie.ac.at/gpi/fungi_server.html.

### Growth rate measurement and reverse transcription polymerase chain reaction detection of CHS expression

To compare the growth rate of *R. oryzae* and *A. fumigatus*, the strains were cultured at 37°C with 10^2^ spores/5 µl inoculation (Table S20). For RT-PCR tests, *R. oryzae* strain CBS 112.07 was inoculated into a MEB medium or on a MEA plate. RNA was isolated from harvested mycelia using ISOGEN (Nippon Gene, Toyma Japan), followed by purification and treatment with DNase. Detection of each chitin synthase gene transcript was performed using RT-PCR amplification with primers specific to the CHS domain sequence of each gene. Amplification was also performed with RNA that was not treated with reverse transcriptase to serve as a control to determine if the amplification product was from DNA contamination. RT-PCR amplification in a 50 µl reaction mixture with 100 ng of RNA was performed using the QIAGEN One-Step RT-PCR Kit (Valencia, CA). The reaction condition was as follows: reverse transcription at 50°C for 30 min, initial PCR activation step at 95°C for 15 min, 30 cycles of denaturing at 94°C for 30 s, annealing at 50°C for 30 s, and extension at 72°C for 1 min. A final 10 min of chain elongation at 72°C was carried out after cycle completion in a model 9700 thermal cycler (Applied Biosystems). The reaction condition was as follows: reverse transcription at 50°C for 30 min, initial PCR activation step at 94°C for 2 min, 40 cycles of denaturing at 94°C for 15 s, annealing at 55°C for 30 s, and extension at 68°C for 2 min. A final 5 min of chain elongation at 68°C was carried out after cycling completion. PCR products were resolved on agarose gels and detected by staining with ethidium bromide (Figure 4).

### Comparative proteomics

The protein sets of fungal genomes including *R. oryzae* (non-TE protein set), *Coprinus cinereus*, *Ustilago maydis*, *Fusarium verticillioides*, and *Neurospora crassa* (http://www.broad.mit.edu/annotation/fungi/fgi/), were searched using BLASTP (E≤10^−20^) against the NCBI metazoan gene sets (combining the mammal, non-mammalian vertebrates and invertebrates) available at ftp://ftp.ncbi.nlm.nih.gov/gene/DATA/GENE_INFO (February 21, 2008 version) and the dikaryotic database, including the protein sets from Ascomycete fungal genomes (*Aspergillus nidulans*, *Botrytis cinerea*, *Chaetomium globosum*, *Coccidioides immitis*, *Fusarium graminearum*, *Magnaporthe grisea*, *Neurospora crassa*, and *Sclerotinia sclerotiorum*, all generated at the Broad) and the Basidiomycete fungal genomes (*Ustilago maydis*, *Coprinus cinereus*, and *Cryptococcus neoformans* serotype A, generated at the Broad; *Phanerochaete chrysosporium* http://genome.jgi-psf.org/whiterot1/whiterot1.home.html and *Laccaria bicolor* http://genome.jgi-psf.org/Lacbi1/Lacbi1.home.html, generated at JGI) (Table S16).

### Kinome characterization

A multi-level hidden Markov model (HMM) library of the protein kinase superfamily was applied to the predicted peptides of *R. oryzae* under the HMMER software suite (v. 2.3.2, http://hmmer.janelia.org), correcting for database size with the ‘-Z’ option. The automatically retrieved sequences were individually inspected and protein kinase homologies were determined by building kinase group-specific phylogenetic trees with the annotated kinomes of *S. cerevisiae*, *S. pombe* and *Encephalitozoon cuniculi* [54].

## Supporting Information

Figure S1 Co-localization of tRNA genes and some transposable elements.
(0.83 MB JPG)

Figure S2 Comparison of protein families among fungal genomes.
(0.44 MB JPG)

Figure S3 Distribution of duplicated regions.
(0.47 MB JPG)

Figure S4 Phylogeny of fungal secreted aspartyl protease (SAP) proteins.
(0.93 MB JPG)

Figure S5 GO annotation of the *R. oryzae* metazoan homologous genes.
(0.76 MB JPG)

Figure S6 Maximum-likelihood tree of the RasGEF proteins.
(1.25 MB JPG)

Figure S7 The diagram for Cdc15p homologue.
(0.45 MB JPG)

Table S1 *Rhizopus oryzae* genome sequence strategy.
(0.05 MB PDF)

Table S2 *R. oryzae* assembly mapped to the optical map.
(0.04 MB PDF)

Table S3 Repeat content in fungal genomes.
(0.05 MB PDF)

Table S4 EST reads corresponding to identified TEs.
(0.04 MB PDF)

Table S5 Syntenic blocks for different distance parameters.
(0.06 MB PDF)

Table S6 *R. oryzae* syntenic regions and gene pairs that define each region.
(0.53 MB PDF)

Table S7 Best-blast hits between *P. blakesleeanus* and *R. oryzae*.
(0.07 MB PDF)

Table S8 GO term enrichment among the retained genes (Fisher's exact tests).
(0.06 MB PDF)

Table S9 Duplication of oxidative phosphorylation protein complexes.
(0.12 MB PDF)

Table S10 Duplication of V-ATPase.
(0.07 MB PDF)

Table S11 Duplication of ubiquitin-proteosome system.
(0.08 MB PDF)

Table S12 Retained genes in both *R. oryzae* and *S. cerevisiae*.
(0.09 MB PDF)

Table S13 Comparison of P-loop GTPases in *R. oryzae* and dikaryotic fungi.
(0.21 MB PDF)

Table S14 Regulators of Ras superfamily GTPases in *R. oryzae* and dikaryotic fungi.
(0.21 MB PDF)

Table S15 Enriched proteases gene families.
(0.08 MB PDF)

Table S16 Annotation of cell wall synthesis enzymes and secreted proteases.
(0.06 MB PDF)

Table S17 Ergosterol biosynthesis pathway in *R. oryzae*.
(0.06 MB PDF)

Table S18 Fungal homologs to Metazoa.
(0.07 MB PDF)

Table S19 Comparison of the core elements of the MEN/SIN pathway.
(0.09 MB PDF)

Table S20 Growth comparison (37°C) of *R. oryzae* 99–880 versus *A. fumigatus* AF293.
(0.08 MB PDF)
