# Supplementary figures and images for: Genomic Analysis of the Basal Lineage Fungus Rhizopus oryzae Reveals a Whole-Genome Duplication
Source: PLoS Genet. 2009 Jul 3;5(7):e1000549. doi: 10.1371/journal.pgen.1000549 (PMC2699053; doi:10.1371/journal.pgen.1000549)

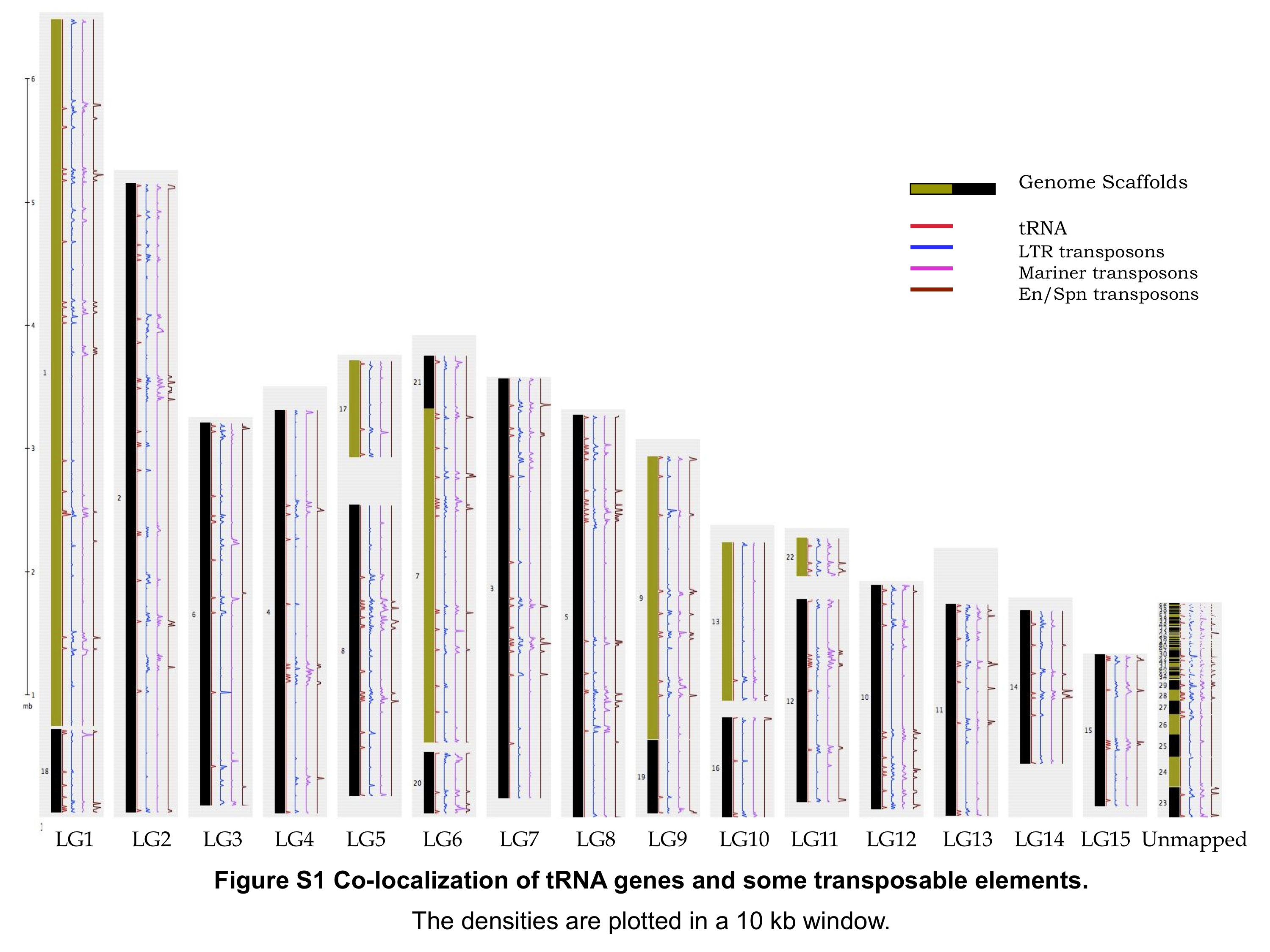

Supplement: Figure S1 — Co-localization of tRNA genes and some transposable elements. (0.83 MB JPG) [file pgen.1000549.s001.jpg]

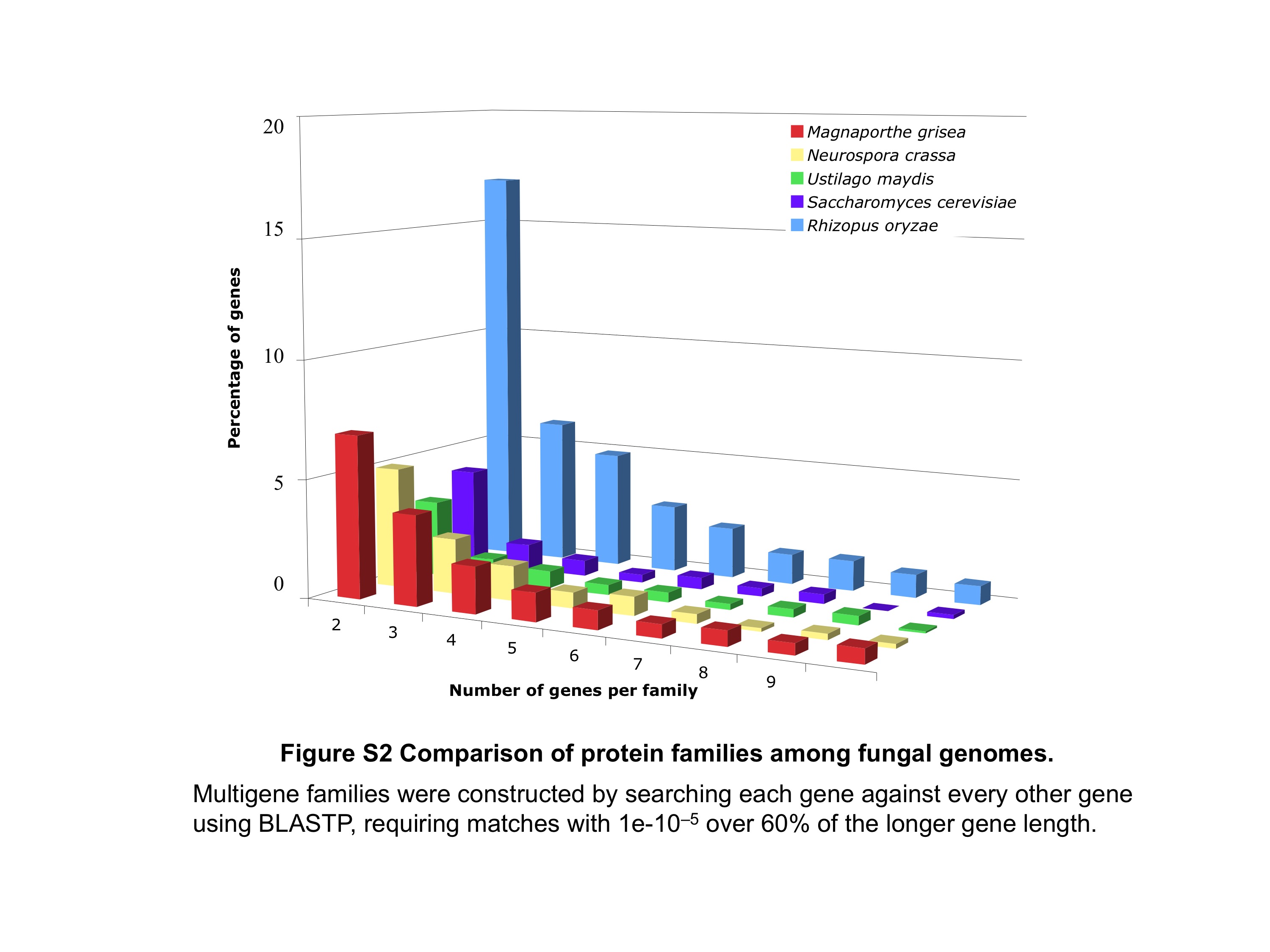

Supplement: Figure S2 — Comparison of protein families among fungal genomes. (0.44 MB JPG) [file pgen.1000549.s002.jpg]

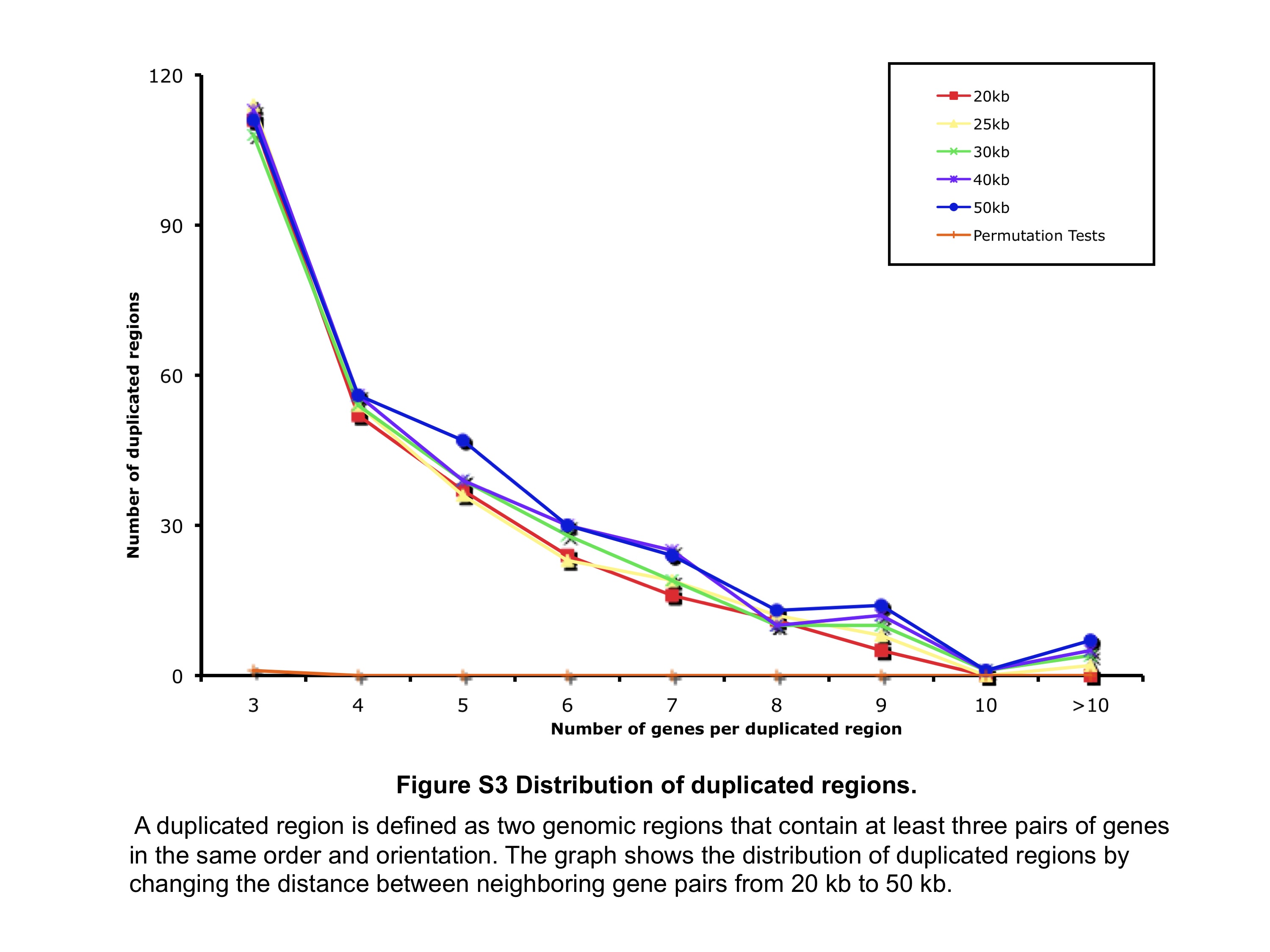

Supplement: Figure S3 — Distribution of duplicated regions. (0.47 MB JPG) [file pgen.1000549.s003.jpg]

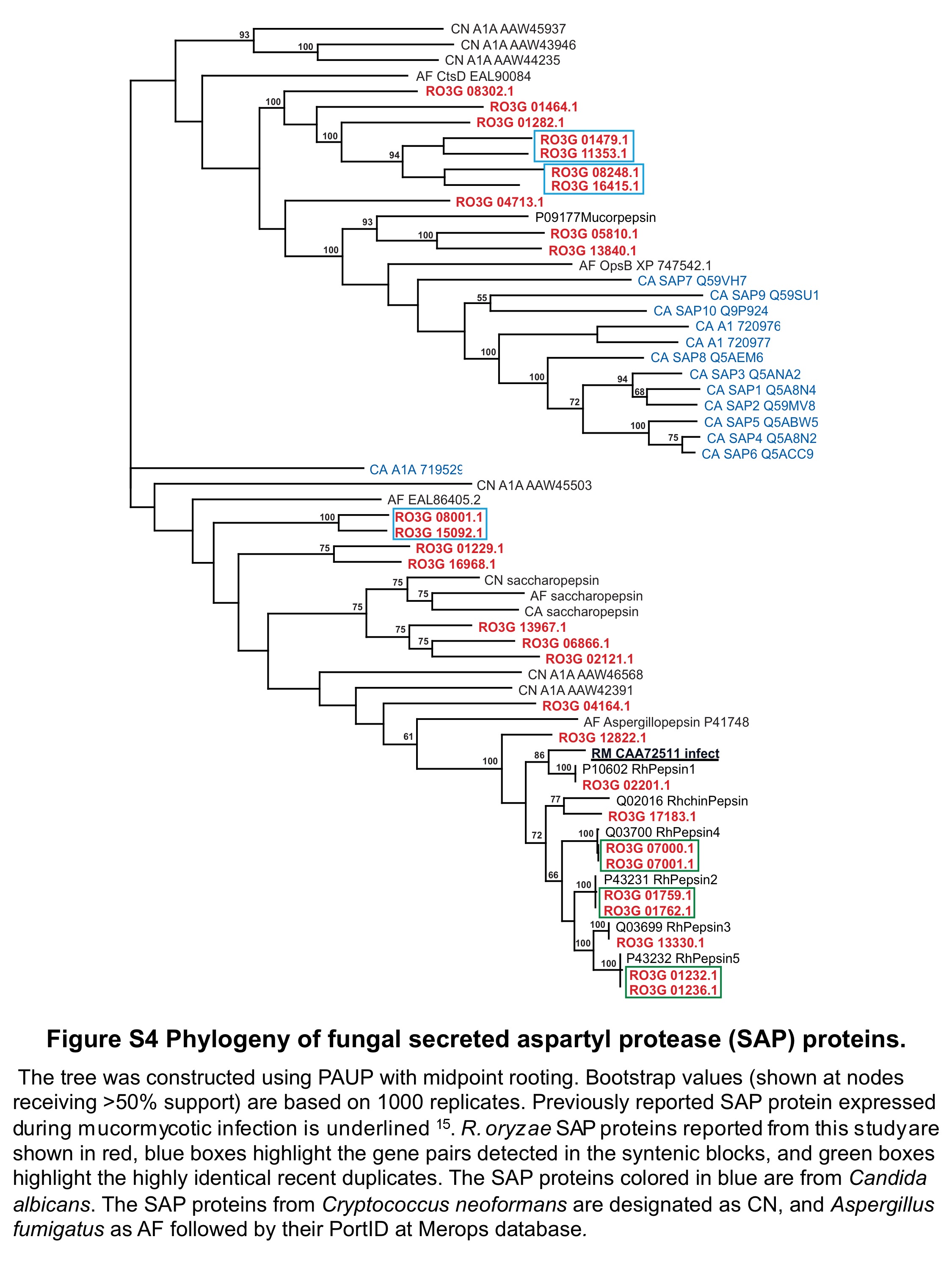

Supplement: Figure S4 — Phylogeny of fungal secreted aspartyl protease (SAP) proteins. (0.93 MB JPG) [file pgen.1000549.s004.jpg]

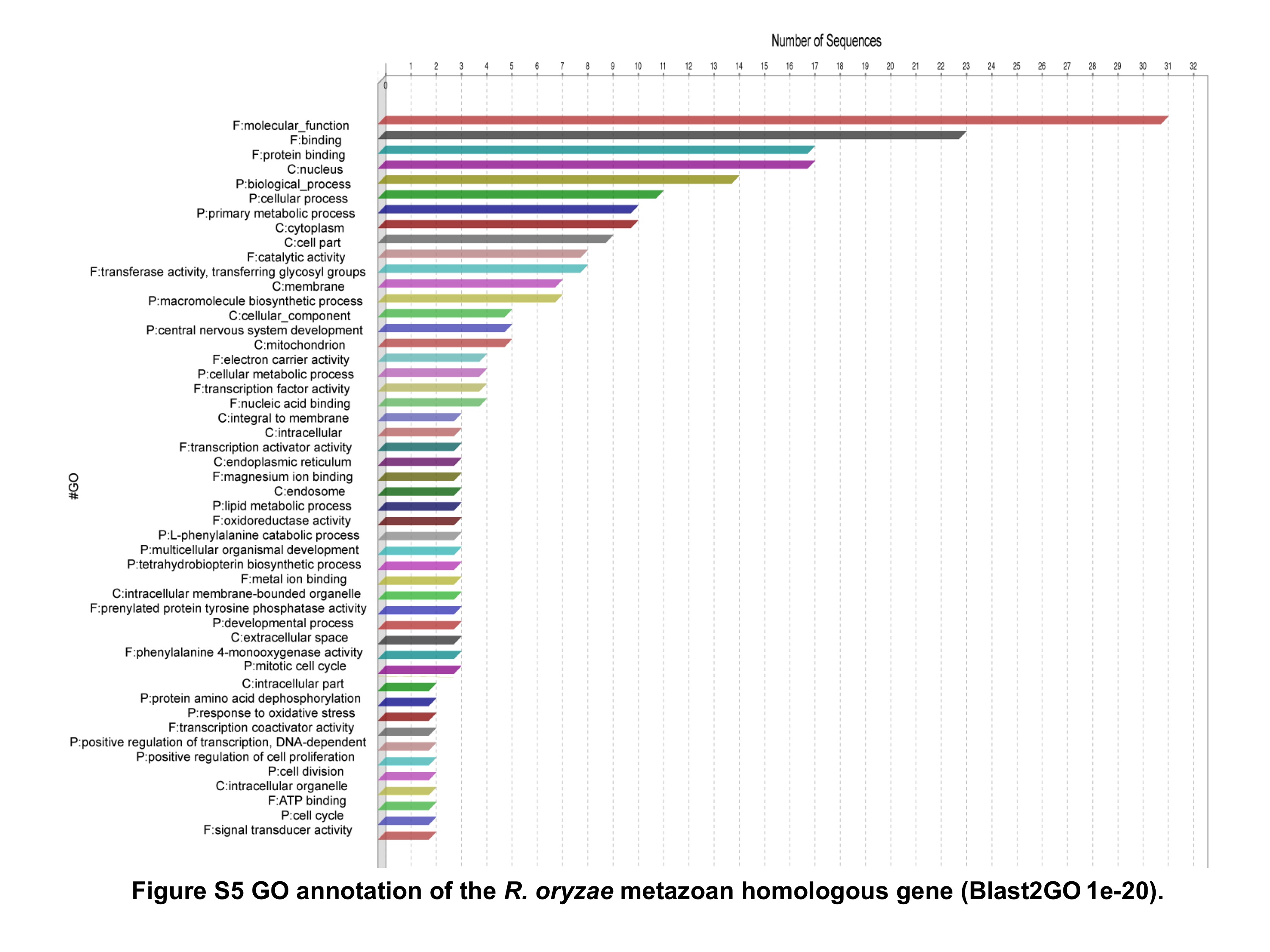

Supplement: Figure S5 — GO annotation of the R. oryzae metazoan homologous genes. (0.76 MB JPG) [file pgen.1000549.s005.jpg]

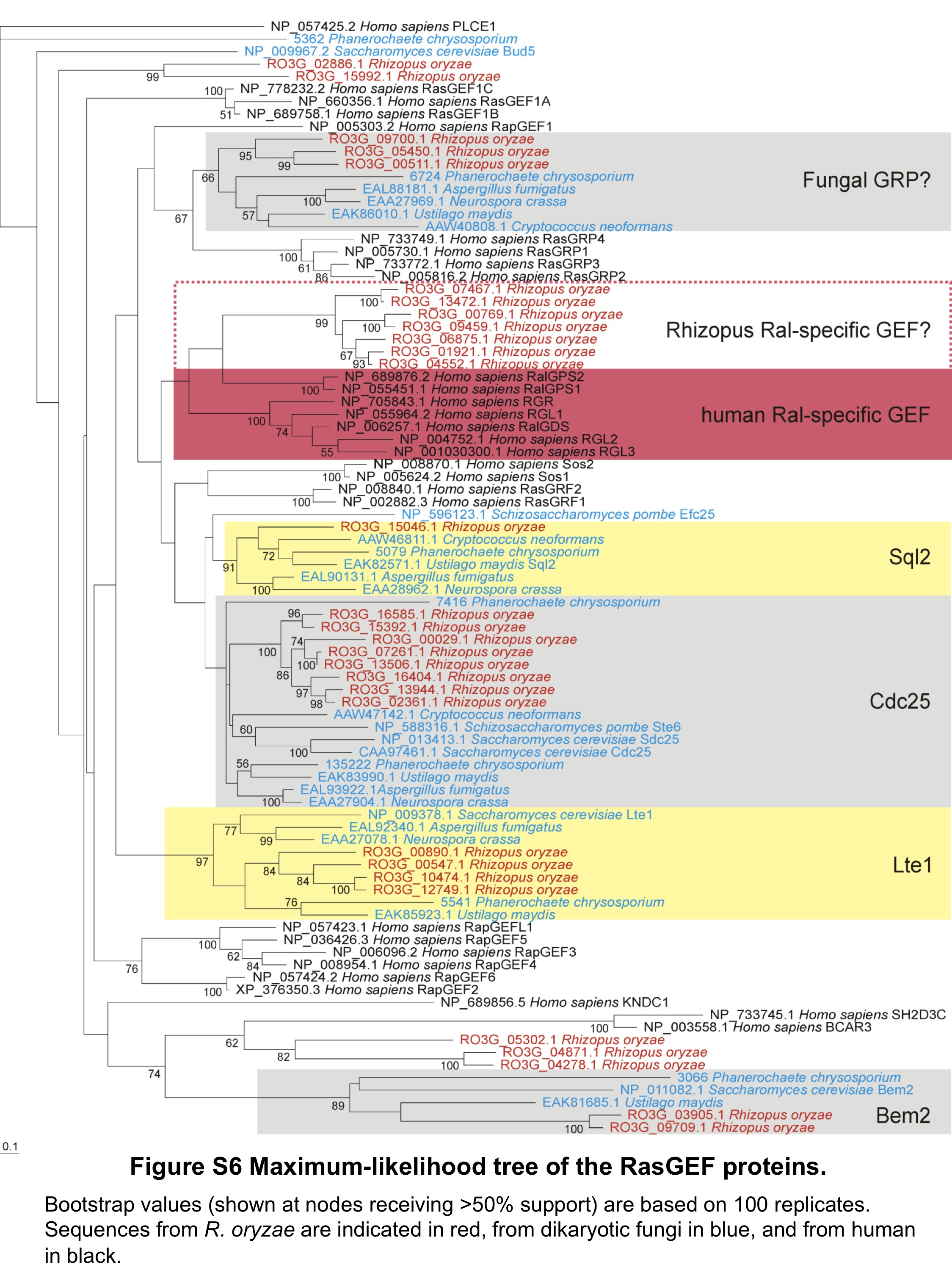

Supplement: Figure S6 — Maximum-likelihood tree of the RasGEF proteins. (1.25 MB JPG) [file pgen.1000549.s006.jpg]

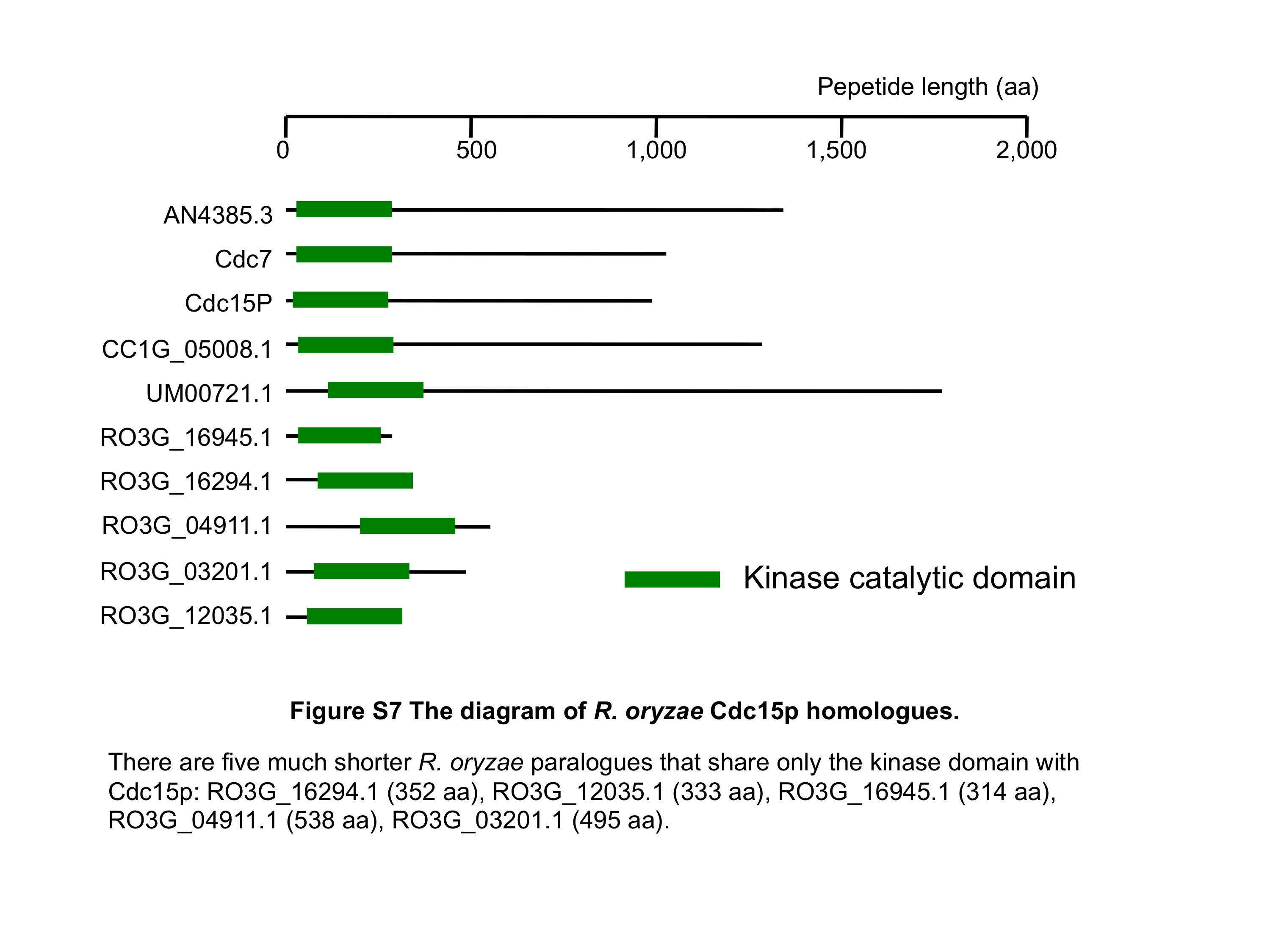

Supplement: Figure S7 — The diagram for Cdc15p homologue. (0.45 MB JPG) [file pgen.1000549.s007.jpg]
