# Supplementary material for: Genomic Analysis of the Basal Lineage Fungus Rhizopus oryzae Reveals a Whole-Genome Duplication
Source: PLoS Genet. 2009 Jul 3;5(7):e1000549. doi: 10.1371/journal.pgen.1000549 (PMC2699053; doi:10.1371/journal.pgen.1000549)
Supplement: Table S1 — Rhizopus oryzae genome sequence strategy. (0.05 MB PDF) [file pgen.1000549.s008.pdf]

Table S1 *Rhizopus oryzae* genome sequence strategy

Library	Reads	Physical Coverage (Fold)	Sequence Coverage (Fold)
4kb Plasmid	437,574	20	6.83
10kb Plasmid	148,413	16	2.17
40kb Fosmid	159,532	70	2.19
Total	747,519	106	11.19
