# Supplementary material for: Genomic Analysis of the Basal Lineage Fungus Rhizopus oryzae Reveals a Whole-Genome Duplication
Source: PLoS Genet. 2009 Jul 3;5(7):e1000549. doi: 10.1371/journal.pgen.1000549 (PMC2699053; doi:10.1371/journal.pgen.1000549)
Supplement: Table S2 — R. oryzae assembly mapped to the optical map. (0.04 MB PDF) [file pgen.1000549.s009.pdf]

Table S2 *R. oryzae* assembly mapped to the optical map

Linkage group	Map estimated size	Scaffolds	Total scaffold size
I	6,487,553	18, 1	6,412,466
II	5,153,661	2	5,107,438
III	3,210,534	6	3,104,132
IV	3,314,588	4	3,274,895
V	3,708,002	8, 17	3,145,915
VI	3,747,038	20, 7, 21	3,640,464
VII	3,572,496	3	3,411,807
VIII	3,278,578	5	3,273,673
IX	2,937,735	19, 9	2,893,547
X	2,242,117	16, 13	2,109,721
XI	2,272,156	12, 22	1,961,337
XII	1,898,532	10	1,823,771
XIII	1,742,552	11	1,718,195
XIV	1,689,232	14	1,243,565
XV	1,332,463	15	1,232,904
**Total**	**46,587,237**		**44,353,830**
