# Supplementary material for: Genomic Analysis of the Basal Lineage Fungus Rhizopus oryzae Reveals a Whole-Genome Duplication
Source: PLoS Genet. 2009 Jul 3;5(7):e1000549. doi: 10.1371/journal.pgen.1000549 (PMC2699053; doi:10.1371/journal.pgen.1000549)
Supplement: Table S3 — Repeat content in fungal genomes. (0.05 MB PDF) [file pgen.1000549.s010.pdf]

**Table S3. Repeat content in fungal genomes**

| Species | Repeat (bases) | Assembly size (bases) | % of repeat |
|---|---|---|---|
| *Rhizopus oryzae* | 15,739,510 | 45,262,477 | 34.77% |
| *Neurospora crassa* | 4,297,191 | 39,225,835 | 10.96% |
| *Magnaporthe grisea* | 3,458,269 | 39,429,053 | 8.77% |
| *Aspergillus nidulans* | 1,315,289 | 30,069,000 | 4.37% |
| *Aspergillus fumigatus* | 2,744,799 | 29,384,958 | 9.34% |
| *Fusarium graminearum* | 150,689 | 36,093,143 | 0.42% |
| *Ustilago maydis* | 618,037 | 19,683,350 | 3.14% |
