# Supplementary material for: Genomic Analysis of the Basal Lineage Fungus Rhizopus oryzae Reveals a Whole-Genome Duplication
Source: PLoS Genet. 2009 Jul 3;5(7):e1000549. doi: 10.1371/journal.pgen.1000549 (PMC2699053; doi:10.1371/journal.pgen.1000549)
Supplement: Table S4 — EST reads corresponding to identified TEs. (0.04 MB PDF) [file pgen.1000549.s011.pdf]

Table S4. EST reads corresponding to identified TEs

TRANSPOSONS	EST READS
LINE-ESTs	ROE00005168, ROE00002059, G774P563FF9.T0, G774P541RC7.T1, G774P541FC7.T1
Mariner-ESTs	ROE00006902, G774P571RF1.T0, G774P571FF1.T0
LTRs	ROE00003814, ROE00002163, ROE00002109
DIRs	G774P552RH1.T0
