# Supplementary material for: Genomic Analysis of the Basal Lineage Fungus Rhizopus oryzae Reveals a Whole-Genome Duplication
Source: PLoS Genet. 2009 Jul 3;5(7):e1000549. doi: 10.1371/journal.pgen.1000549 (PMC2699053; doi:10.1371/journal.pgen.1000549)
Supplement: Table S5 — Syntenic blocks for different distance parameters. (0.06 MB PDF) [file pgen.1000549.s012.pdf]

**Table S5 Summary of synteny testing different distance parameters**

| Distance [a] | Duplicated Regions[b] | Expected Triplets [c] | Observed Triplets | Max Genes /Block | Average genes per block | Average size per block (kb) | Genomic coverage (%) |
|---|---|---|---|---|---|---|---|
| 10,000 | 174 | 32 | 0 | 8 | 3.9 | 5 | 10.8 |
| **20,000** | **256** | **47** | **3** | **9** | **4.7** | **57** | **12.2** |
| 30,000 | 273 | 50 | 10 | 16 | 4.7 | 57 | 16.9 |
| 40,000 | 291 | 54 | 20 | 16 | 4.8 | 64 | 20.3 |
| 50,000 | 303 | 56 | 26 | 16 | 4.9 | 81 | 21.7 |

[a] The distance between two neighboring homologous gene pairs that share the same order and orientation in two genomic locations;
[b] The genomic regions that contain at least three homologous gene pairs with another genomic region;
[c] The expected triplets according the a Poisson distribution.
