# Supplementary material for: Genomic Analysis of the Basal Lineage Fungus Rhizopus oryzae Reveals a Whole-Genome Duplication
Source: PLoS Genet. 2009 Jul 3;5(7):e1000549. doi: 10.1371/journal.pgen.1000549 (PMC2699053; doi:10.1371/journal.pgen.1000549)
Supplement: Table S6 — R. oryzae syntenic regions and gene pairs that define each region. (0.53 MB PDF) [file pgen.1000549.s013.pdf]

Table S6 *R. oryzae* syntenic regions and gene pairs that define each region*

##Scaffold_1	Scaffold_1	5139455	5173290	1	2074640	2099070	-1	3		
5146698	5147430	2074640	2075870	1	-1	RO3G_01993.1	RO3G_00815.1	251	227	54.19
5139455	5140868	2084667	2085932	1	-1	RO3G_01991.1	RO3G_00819.1	665	381	78.48
5172250	5173290	2097882	2099070	-1	1	RO3G_02005.1	RO3G_00826.1	313	207	74.4
##Scaffold_1	Scaffold_19	1327841	1352787	19	239215	256226	-1	3		
1349741	1352787	239215	242170	1	-1	RO3G_00534.1	RO3G_16214.1	1506	938	79.42
1347735	1349468	245057	246785	1	-1	RO3G_00533.1	RO3G_16215.1	876	458	97.6
1327841	1328549	255346	256226	1	-1	RO3G_00525.1	RO3G_16220.1	296	186	80.11
##Scaffold_1	Scaffold_19	2462451	2479793	19	154590	169639	-1	4		
2479301	2479793	154590	155123	-1	1	RO3G_00980.1	RO3G_16178.1	133	165	63.64
2477159	2479279	155903	156742	1	-1	RO3G_00979.1	RO3G_16179.1	297	210	72.38
2475188	2476387	157429	160018	1	-1	RO3G_00978.1	RO3G_16180.1	332	375	58.4
2462451	2464245	167813	169639	-1	1	RO3G_00974.1	RO3G_16183.1	739	572	73.95
##Scaffold_1	Scaffold_19	2553337	2590631	19	180637	192629	-1	3		
2589972	2590631	180637	181650	-1	1	RO3G_01024.1	RO3G_16188.1	157	173	66.47
2587587	2588491	182690	183434	1	-1	RO3G_01022.1	RO3G_16189.1	139	210	47.62
2553337	2558893	187928	192629	-1	1	RO3G_01012.1	RO3G_16191.1	1066	1496	44.25
##Scaffold_1	Scaffold_19	3468924	3508159	19	104727	133238	-1	4		
3507656	3508159	104727	105591	1	-1	RO3G_01342.1	RO3G_16167.1	290	167	85.03
3487398	3489073	115207	116988	-1	1	RO3G_01334.1	RO3G_16170.1	665	373	82.84
3482785	3484068	117402	129254	1	-1	RO3G_01332.1	RO3G_16171.1	796	427	98.59
3468924	3470087	132259	133238	-1	1	RO3G_01331.1	RO3G_16172.1	317	291	59.11
##Scaffold_1	Scaffold_27	5632797	5642580	27	49535	57203	-1	3		
5641498	5642580	49535	50416	-1	1	RO3G_02194.1	RO3G_17174.1	384	197	98.98
5640603	5641416	50723	51617	-1	1	RO3G_02193.1	RO3G_17175.1	266	180	70.56
5632797	5633430	56831	57203	-1	1	RO3G_02190.1	RO3G_17179.1	114	65	86.15
##Scaffold_1	Scaffold_3	1654328	1697462	3	3028752	3069039	-1	7		
1697065	1697462	3028752	3029256	1	-1	RO3G_00673.1	RO3G_05370.1	158	88	90.91
1692207	1693682	3038913	3039469	-1	1	RO3G_00670.1	RO3G_05373.1	171	95	81.05
1685798	1686784	3040122	3041439	1	-1	RO3G_00668.1	RO3G_05374.1	498	328	80.18
1681663	1682304	3042781	3043734	-1	1	RO3G_00666.1	RO3G_05375.1	301	217	76.5
1678789	1679370	3046924	3047825	-1	1	RO3G_00665.1	RO3G_05377.1	244	176	80.11
1674847	1675966	3051868	3052787	-1	1	RO3G_00664.1	RO3G_05379.1	226	192	70.83
1654328	1655871	3067883	3069039	-1	1	RO3G_00656.1	RO3G_05385.1	308	249	64.66
##Scaffold_1	Scaffold_3	1834274	1857190	3	3316695	3333906	1	6		
1834274	1835377	3316695	3317390	-1	-1	RO3G_00729.1	RO3G_05476.1	251	365	43.84
1838611	1839346	3318802	3319503	1	1	RO3G_00731.1	RO3G_05477.1	283	149	95.3
1839413	1840165	3319846	3320141	-1	-1	RO3G_00732.1	RO3G_05478.1	117	84	64.29
1849249	1849545	3324668	3324967	1	1	RO3G_00735.1	RO3G_05480.1	161	97	81.44
1849981	1851664	3325339	3326298	-1	-1	RO3G_00736.1	RO3G_05481.1	449	291	84.19
1855837	1857190	3332302	3333906	1	1	RO3G_00739.1	RO3G_05484.1	646	340	97.06
##Scaffold_1	Scaffold_3	2232959	2241323	3	2823434	2832525	-1	3		
2240573	2241323	2823434	2824187	1	-1	RO3G_00883.1	RO3G_05289.1	324	179	88.83
2236971	2238474	2825378	2826729	1	-1	RO3G_00881.1	RO3G_05291.1	733	403	87.1
2232959	2234484	2831007	2832525	1	-1	RO3G_00880.1	RO3G_05293.1	825	489	86.71
##Scaffold_1	Scaffold_3	2274523	2279729	3	2154560	2160229	-1	3		
2279002	2279729	2154560	2155278	-1	1	RO3G_00897.1	RO3G_05028.1	232	199	66.33
2277619	2278794	2155500	2156770	1	-1	RO3G_00896.1	RO3G_05029.1	379	360	63.61
2274523	2277180	2159090	2160229	-1	1	RO3G_00895.1	RO3G_05030.1	613	374	80.48
##Scaffold_1	Scaffold_3	2290435	2301669	3	2693474	2709438	1	3		
2290435	2291150	2693474	2694712	1	1	RO3G_00900.1	RO3G_05242.1	322	197	83.25
2293685	2295456	2697565	2699340	1	1	RO3G_00902.1	RO3G_05244.1	867	496	89.31
2301025	2301669	2708632	2709438	-1	-1	RO3G_00905.1	RO3G_05250.1	311	206	70.39
##Scaffold_1	Scaffold_3	4322746	4339475	3	1739965	1759639	1	4		
4322746	4325046	1739965	1741648	1	1	RO3G_01670.1	RO3G_04858.1	689	427	78.22
4328025	4329658	1752045	1753673	-1	-1	RO3G_01672.1	RO3G_04863.1	954	485	99.18
4335837	4337834	1755449	1757983	1	1	RO3G_01675.1	RO3G_04864.1	858	465	90.32
4339019	4339475	1758159	1759639	-1	-1	RO3G_01677.1	RO3G_04865.1	229	133	83.46
##Scaffold_1	Scaffold_3	4344355	4363763	3	2063883	2096295	-1	4		
4362564	4363763	2063883	2065638	1	-1	RO3G_01686.1	RO3G_04991.1	457	325	73.23
4358023	4358445	2076248	2076673	1	-1	RO3G_01685.1	RO3G_04997.1	150	139	79.14
4348205	4350856	2081332	2085311	-1	1	RO3G_01681.1	RO3G_04999.1	1070	816	70.47
4344355	4344792	2095197	2096295	-1	1	RO3G_01679.1	RO3G_05005.1	211	132	81.06

##Scaffold_1	Scaffold_3	4373012	4384804	3	2029046	2051071	-1	3		
4383902	4384804	2029046	2029743	-1	1	RO3G_01694.1	RO3G_04976.1	216	185	61.08
4377771	4380355	2041508	2044323	-1	1	RO3G_01693.1	RO3G_04980.1	1079	752	80.72
4373012	4374778	2049740	2051071	-1	1	RO3G_01691.1	RO3G_04984.1	241	264	48.48
##Scaffold_1	Scaffold_4	62010	84064	4	1497289	1516902	1	4		
62010	63496	1497289	1498647	-1	-1	RO3G_00024.1	RO3G_06049.1	637	338	93.2
63914	65025	1498815	1501111	-1	-1	RO3G_00025.1	RO3G_06050.1	560	348	82.47
78434	79817	1510755	1512183	1	1	RO3G_00031.1	RO3G_06055.1	542	439	59
83378	84064	1515966	1516902	1	1	RO3G_00034.1	RO3G_06056.1	182	145	62.76
##Scaffold_1	Scaffold_4	99170	105388	4	1535524	1540887	-1	3		
104076	105388	1535524	1538325	1	-1	RO3G_00045.1	RO3G_06063.1	411	375	58.13
100036	100341	1539669	1539997	-1	1	RO3G_00043.1	RO3G_06064.1	108	106	55.66
99170	99753	1540296	1540887	1	-1	RO3G_00042.1	RO3G_06065.1	261	161	91.93
##Scaffold_1	Scaffold_4	1070409	1088933	4	1833814	1854876	1	4		
1070409	1070876	1833814	1834281	1	1	RO3G_00430.1	RO3G_06177.1	246	155	96.77
1081320	1082875	1847158	1848769	1	1	RO3G_00434.1	RO3G_06181.1	748	472	77.33
1083195	1083873	1849807	1850490	1	1	RO3G_00435.1	RO3G_06182.1	189	187	68.45
1087478	1088933	1853380	1854876	-1	-1	RO3G_00437.1	RO3G_06184.1	516	348	70.69
##Scaffold_1	Scaffold_4	1168571	1176522	4	2138900	2151664	1	3		
1168571	1169602	2138900	2140152	1	1	RO3G_00461.1	RO3G_06287.1	294	301	56.81
1170026	1172275	2142082	2144478	-1	-1	RO3G_00462.1	RO3G_06289.1	1031	758	69.66
1174907	1176522	2150054	2151664	1	1	RO3G_00463.1	RO3G_06292.1	991	520	91.54
##Scaffold_1	Scaffold_4	2972202	3006213	4	164669	209951	1	7		
2972202	2972976	164669	165459	-1	-1	RO3G_01173.1	RO3G_05587.1	135	98	72.45
2973788	2977012	168658	172910	1	1	RO3G_01174.1	RO3G_05589.1	870	693	66.96
2977942	2979738	173228	175047	-1	-1	RO3G_01175.1	RO3G_05590.1	659	518	74.9
2980190	2987338	177360	185781	-1	-1	RO3G_01176.1	RO3G_05593.1	1911	1562	68.63
2994842	2995714	191623	193127	1	1	RO3G_01181.1	RO3G_05595.1	332	226	74.78
3001057	3002940	205978	207794	-1	-1	RO3G_01184.1	RO3G_05600.1	605	522	60.73
3004847	3006213	208364	209951	-1	-1	RO3G_01185.1	RO3G_05601.1	329	509	52.85
##Scaffold_1	Scaffold_5	1890578	1912849	5	1176735	1208769	-1	3		
1912091	1912849	1176735	1177506	1	-1	RO3G_00757.1	RO3G_07197.1	352	217	78.8
1898080	1900752	1196136	1199115	1	-1	RO3G_00753.1	RO3G_07203.1	944	840	69.05
1890578	1892979	1206610	1208769	-1	1	RO3G_00751.1	RO3G_07207.1	993	618	78.96
##Scaffold_1	Scaffold_5	2397795	2406846	5	1102946	1129110	-1	3		
2405151	2406846	1102946	1105346	-1	1	RO3G_00950.1	RO3G_07163.1	893	505	87.33
2401358	2404385	1105862	1109546	-1	1	RO3G_00949.1	RO3G_07164.1	216	136	80.15
2397795	2398214	1128552	1129110	-1	1	RO3G_00946.1	RO3G_07174.1	153	83	83.13
##Scaffold_1	Scaffold_5	2424381	2431836	5	1163181	1168487	-1	3		
2430998	2431836	1163181	1164072	-1	1	RO3G_00960.1	RO3G_07189.1	235	292	47.6
2425616	2426098	1166410	1167443	1	-1	RO3G_00957.1	RO3G_07192.1	114	85	67.06
2424381	2424943	1168072	1168487	1	-1	RO3G_00955.1	RO3G_07193.1	175	90	88.89
##Scaffold_1	Scaffold_5	2614313	2623817	5	1241736	1247214	-1	3		
2623035	2623817	1241736	1242539	1	-1	RO3G_01036.1	RO3G_07222.1	237	117	98.29
2620445	2620915	1244986	1245436	-1	1	RO3G_01034.1	RO3G_07224.1	226	111	97.3
2614313	2614637	1246586	1247214	1	-1	RO3G_01033.1	RO3G_07225.1	157	88	86.36
##Scaffold_1	Scaffold_5	4846119	4857658	5	284891	294155	1	3		
4846119	4853721	284891	291199	1	1	RO3G_01878.1	RO3G_06842.1	2300	2107	61.32
4853981	4854441	292625	293021	1	1	RO3G_01879.1	RO3G_06844.1	135	108	62.04
4856675	4857658	293315	294155	1	1	RO3G_01881.1	RO3G_06845.1	519	264	94.32
##Scaffold_1	Scaffold_6	394357	414176	6	399814	429722	1	4		
394357	395425	399814	400037	1	1	RO3G_00161.1	RO3G_08130.1	103	58	77.59
400698	401562	406132	406827	1	1	RO3G_00163.1	RO3G_08133.1	202	183	60.66
410089	410917	421528	422334	1	1	RO3G_00166.1	RO3G_08137.1	236	160	82.5
411492	414176	426908	429722	1	1	RO3G_00167.1	RO3G_08138.1	1179	702	86.47
##Scaffold_1	Scaffold_6	486036	533978	6	847739	882999	1	6		
486036	486775	847739	848993	-1	-1	RO3G_00194.1	RO3G_08318.1	389	211	93.36
494115	495180	850808	851873	1	1	RO3G_00198.1	RO3G_08319.1	652	337	99.41
509648	511095	859296	860906	-1	-1	RO3G_00204.1	RO3G_08323.1	818	417	92.09
517085	517463	863283	863649	-1	-1	RO3G_00209.1	RO3G_08326.1	135	69	98.55
525489	526277	871977	872666	1	1	RO3G_00212.1	RO3G_08331.1	351	264	64.77
533256	533978	881722	882999	-1	-1	RO3G_00214.1	RO3G_08336.1	303	242	67.36
##Scaffold_1	Scaffold_6	654546	678588	6	955647	981199	1	6		

654546	656393	955647	957410	1	1	RO3G_00266.1	RO3G_08359.1	649	579	67.7
658739	661879	959039	962179	1	1	RO3G_00267.1	RO3G_08360.1	1818	1006	92.84
666450	667721	964238	965584	-1	-1	RO3G_00269.1	RO3G_08362.1	585	449	74.83
668103	668718	965816	966444	-1	-1	RO3G_00270.1	RO3G_08363.1	142	110	100
669230	669983	968865	969749	-1	-1	RO3G_00271.1	RO3G_08365.1	187	203	52.71
672180	678588	974037	981199	1	1	RO3G_00272.1	RO3G_08367.1	2977	2012	77.34
##Scaffold_1	Scaffold_6	785073	807634	6	1153773	1173157	1	4		
785073	789371	1153773	1157135	1	1	RO3G_00315.1	RO3G_08439.1	1686	929	92.9
789436	790032	1157426	1158028	-1	-1	RO3G_00316.1	RO3G_08440.1	266	171	87.13
804010	805187	1168075	1169404	1	1	RO3G_00319.1	RO3G_08444.1	313	226	64.6
805880	807634	1171385	1173157	1	1	RO3G_00320.1	RO3G_08446.1	486	524	56.87
##Scaffold_1	Scaffold_6	829553	846791	6	1073857	1091838	-1	6		
845284	846791	1073857	1075341	1	-1	RO3G_00340.1	RO3G_08406.1	800	456	87.28
843437	845082	1075501	1077035	1	-1	RO3G_00339.1	RO3G_08407.1	373	246	81.71
841490	842916	1077739	1079358	-1	1	RO3G_00338.1	RO3G_08408.1	427	399	56.39
837684	838528	1080867	1081686	1	-1	RO3G_00336.1	RO3G_08410.1	452	255	99.61
832002	832837	1086848	1087787	1	-1	RO3G_00334.1	RO3G_08413.1	253	267	61.8
829553	830746	1090596	1091838	-1	1	RO3G_00333.1	RO3G_08414.1	397	301	79.73
##Scaffold_1	Scaffold_6	810392	817733	6	1102415	1118141	-1	3		
816074	817733	1102415	1104643	-1	1	RO3G_00326.1	RO3G_08421.1	547	515	58.25
811849	813439	1113209	1113858	1	-1	RO3G_00324.1	RO3G_08424.1	166	135	57.78
810392	811004	1117521	1118141	1	-1	RO3G_00323.1	RO3G_08426.1	386	187	98.93
##Scaffold_1	Scaffold_6	935161	969261	6	1198522	1217859	1	5		
935161	935913	1198522	1199378	-1	-1	RO3G_00379.1	RO3G_08459.1	255	201	68.16
942750	943529	1204967	1206097	1	1	RO3G_00383.1	RO3G_08461.1	385	242	72.31
955533	957527	1207551	1209769	-1	-1	RO3G_00389.1	RO3G_08462.1	496	434	59.91
958010	959884	1210170	1212004	-1	-1	RO3G_00390.1	RO3G_08463.1	594	513	66.86
968827	969261	1216779	1217859	-1	-1	RO3G_00393.1	RO3G_08466.1	185	94	95.74
##Scaffold_1	Scaffold_7	1988610	2001432	7	693558	704247	1	4		
1988610	1989401	693558	694330	-1	-1	RO3G_00782.1	RO3G_09466.1	262	139	97.12
1990975	1992436	696908	697807	1	1	RO3G_00784.1	RO3G_09467.1	348	241	71.78
1996455	1998262	700677	702201	-1	-1	RO3G_00787.1	RO3G_09469.1	806	459	89.11
1999999	2001432	702821	704247	-1	-1	RO3G_00788.1	RO3G_09470.1	908	458	94.32
##Scaffold_1	Scaffold_7	2357046	2395646	7	616962	648491	1	7		
2357046	2358601	616962	618451	-1	-1	RO3G_00928.1	RO3G_09434.1	313	335	51.04
2362777	2363787	621022	622099	1	1	RO3G_00930.1	RO3G_09435.1	146	176	50.57
2377959	2378477	625083	625598	-1	-1	RO3G_00935.1	RO3G_09437.1	182	172	66.86
2381629	2382382	632491	633266	-1	-1	RO3G_00937.1	RO3G_09441.1	329	194	84.54
2383673	2384366	638102	638791	1	1	RO3G_00938.1	RO3G_09443.1	227	129	100
2386729	2388117	642076	643556	1	1	RO3G_00940.1	RO3G_09445.1	225	159	78.62
2394231	2395646	647547	648491	1	1	RO3G_00944.1	RO3G_09448.1	127	301	33.55
##Scaffold_1	Scaffold_7	2829063	2837823	7	709382	719654	1	3		
2829063	2830103	709382	710410	1	1	RO3G_01119.1	RO3G_09474.1	427	357	68.63
2830284	2831463	711026	712238	1	1	RO3G_01120.1	RO3G_09475.1	506	355	72.39
2835635	2837823	717503	719654	1	1	RO3G_01122.1	RO3G_09477.1	939	546	87
##Scaffold_1	Scaffold_8	4065932	4085664	8	1292334	1312917	1	4		
4065932	4066982	1292334	1293342	-1	-1	RO3G_01567.1	RO3G_10690.1	315	321	52.96
4069170	4070147	1293689	1295221	-1	-1	RO3G_01568.1	RO3G_10691.1	173	239	47.7
4080081	4082695	1308108	1310699	1	1	RO3G_01572.1	RO3G_10698.1	1269	729	89.85
4084840	4085664	1312075	1312917	-1	-1	RO3G_01575.1	RO3G_10700.1	375	233	78.97
##Scaffold_1	Scaffold_9	3850424	3897497	9	720021	733759	-1	3		
3850424	3852096	720021	721521	1	-1	RO3G_01479.1	RO3G_11353.1	514	444	64.64
3896219	3897497	725339	726915	1	-1	RO3G_01499.1	RO3G_11355.1	715	373	97.32
3892367	3893677	732951	733759	1	-1	RO3G_01501.1	RO3G_11359.1	190	215	56.28
##Scaffold_10	Scaffold_10	1080258	1103313	10	1734757	1756923	1	3		
1080258	1081360	1734757	1735857	1	1	RO3G_12371.1	RO3G_12626.1	472	255	93.33
1086319	1086831	1747000	1747530	1	1	RO3G_12374.1	RO3G_12632.1	139	83	87.95
1102298	1103313	1755625	1756923	-1	-1	RO3G_12378.1	RO3G_12635.1	373	311	63.34
##Scaffold_10	Scaffold_12	683317	704568	12	204914	223550	-1	5		
701979	704568	204914	207685	-1	1	RO3G_12218.1	RO3G_13371.1	1310	798	89.22
695324	696896	208449	210072	1	-1	RO3G_12215.1	RO3G_13372.1	725	490	77.96
693559	695058	210400	211851	1	-1	RO3G_12214.1	RO3G_13373.1	518	344	79.36
691409	691672	217374	217971	-1	1	RO3G_12212.1	RO3G_13375.1	137	87	78.16
683317	685229	221585	223550	-1	1	RO3G_12210.1	RO3G_13377.1	1088	565	99.47
##Scaffold_10	Scaffold_13	1559584	1587356	13	370923	409571	-1	5		
1586151	1587356	370923	372211	1	-1	RO3G_12562.1	RO3G_13996.1	408	354	66.1
1580956	1582178	375414	376617	1	-1	RO3G_12560.1	RO3G_13999.1	398	368	71.74

1573558	1574905	381164	382504	1	-1	RO3G_12558.1	RO3G_14002.1	754	375	100
1565236	1567832	398112	400596	-1	1	RO3G_12555.1	RO3G_14007.1	823	779	61.23
1559584	1560042	409135	409571	-1	1	RO3G_12552.1	RO3G_14011.1	227	120	95
##Scaffold_10	Scaffold_2	853442	861327	2	650185	656949	1	3		
853442	855061	650185	651794	-1	-1	RO3G_12286.1	RO3G_02460.1	904	502	91.63
855373	858361	652381	655406	-1	-1	RO3G_12287.1	RO3G_02461.1	988	806	65.01
860803	861327	656433	656949	-1	-1	RO3G_12289.1	RO3G_02462.1	309	154	99.35
##Scaffold_10	Scaffold_5	387243	405997	5	2760005	2778261	-1	4		
405241	405997	2760005	2760738	-1	1	RO3G_12100.1	RO3G_07776.1	175	105	77.14
393939	396779	2767509	2769452	-1	1	RO3G_12097.1	RO3G_07777.1	373	639	40.06
388049	389359	2776151	2777449	-1	1	RO3G_12094.1	RO3G_07780.1	455	437	68.65
387243	387683	2777569	2778261	-1	1	RO3G_12093.1	RO3G_07781.1	124	144	48.61
##Scaffold_10	Scaffold_5	1655515	1693883	5	3069201	3100696	-1	5		
1693497	1693883	3069201	3069577	-1	1	RO3G_12604.1	RO3G_07891.1	175	86	100
1691835	1692419	3073085	3073411	1	-1	RO3G_12603.1	RO3G_07893.1	114	91	61.54
1687967	1690641	3076010	3078755	-1	1	RO3G_12602.1	RO3G_07895.1	1468	813	98.52
1668749	1671536	3084734	3086882	1	-1	RO3G_12596.1	RO3G_07899.1	755	546	79.49
1655515	1656142	3100069	3100696	-1	1	RO3G_12591.1	RO3G_07906.1	336	193	94.82
##Scaffold_10	Scaffold_7	1409123	1424946	7	1614670	1640631	-1	4		
1423894	1424946	1614670	1615725	1	-1	RO3G_12503.1	RO3G_09808.1	130	325	34.15
1419154	1419905	1626622	1627394	-1	1	RO3G_12500.1	RO3G_09814.1	200	190	53.68
1414910	1415732	1632073	1632916	1	-1	RO3G_12499.1	RO3G_09817.1	377	190	95.26
1409123	1410140	1639892	1640631	1	-1	RO3G_12497.1	RO3G_09820.1	222	232	48.28
##Scaffold_11	Scaffold_10	1371433	1423042	10	1362372	1376242	-1	3		
1422038	1423042	1362372	1363564	-1	1	RO3G_13213.1	RO3G_12477.1	270	393	45.8
1377993	1380222	1368339	1370237	-1	1	RO3G_13197.1	RO3G_12481.1	878	476	97.27
1371433	1375364	1372203	1376242	1	-1	RO3G_13195.1	RO3G_12484.1	1566	1297	70.24
##Scaffold_11	Scaffold_13	1522316	1555103	13	71703	97727	1	4		
1522316	1523426	71703	73094	1	1	RO3G_13239.1	RO3G_13885.1	285	302	53.97
1526851	1528704	80562	82458	1	1	RO3G_13242.1	RO3G_13888.1	1063	548	99.27
1531811	1536534	85756	90382	-1	-1	RO3G_13244.1	RO3G_13890.1	2151	1476	79.61
1553598	1555103	96430	97727	-1	-1	RO3G_13253.1	RO3G_13893.1	529	385	72.47
##Scaffold_11	Scaffold_2	1319791	1326508	2	3312999	3317753	1	3		
1319791	1321792	3312999	3314956	1	1	RO3G_13175.1	RO3G_03536.1	1102	626	88.02
1325085	1325680	3315374	3316657	1	1	RO3G_13177.1	RO3G_03537.1	193	101	86.14
1326012	1326508	3317204	3317753	1	1	RO3G_13178.1	RO3G_03538.1	283	147	96.6
##Scaffold_11	Scaffold_7	357498	408416	7	1279187	1307505	-1	5		
365459	366081	1279187	1280407	-1	1	RO3G_12813.1	RO3G_09688.1	186	115	80
357498	359891	1284203	1284685	-1	1	RO3G_12810.1	RO3G_09690.1	201	120	84.17
407240	408416	1303245	1304608	1	-1	RO3G_12831.1	RO3G_09697.1	497	338	84.91
396375	396666	1304945	1305240	-1	1	RO3G_12827.1	RO3G_09698.1	128	58	100
395597	396202	1305340	1307505	1	-1	RO3G_12826.1	RO3G_09699.1	351	202	89.11
##Scaffold_11	Scaffold_7	455270	467845	7	968739	983700	1	4		
455270	457630	968739	971172	-1	-1	RO3G_12847.1	RO3G_09565.1	597	716	54.47
460827	461661	975546	976061	1	1	RO3G_12849.1	RO3G_09567.1	282	146	93.84
463814	465216	979475	980824	1	1	RO3G_12852.1	RO3G_09569.1	464	323	76.47
466206	467845	982544	983700	-1	-1	RO3G_12853.1	RO3G_09570.1	274	211	70.14
##Scaffold_11	Scaffold_7	793284	825051	7	2012829	2040537	1	5		
793284	794731	2012829	2014011	-1	-1	RO3G_12983.1	RO3G_09971.1	332	251	69.32
798438	800350	2017609	2018681	-1	-1	RO3G_12984.1	RO3G_09974.1	347	299	66.56
810030	811428	2021520	2022797	1	1	RO3G_12988.1	RO3G_09976.1	351	344	51.74
812979	813735	2024581	2025308	1	1	RO3G_12989.1	RO3G_09978.1	329	233	81.55
824237	825051	2039276	2040537	1	1	RO3G_12994.1	RO3G_09984.1	330	196	80.1
##Scaffold_11	Scaffold_7	919304	956342	7	2454580	2477967	-1	5		
954473	956342	2454580	2456418	-1	1	RO3G_13040.1	RO3G_10147.1	882	515	82.52
945093	946170	2459195	2460075	1	-1	RO3G_13037.1	RO3G_10149.1	125	95	68.42
926036	930545	2464669	2470275	1	-1	RO3G_13033.1	RO3G_10151.1	2404	1422	84.46
920889	923845	2471185	2475124	-1	1	RO3G_13032.1	RO3G_10152.1	976	834	63.07
919304	920398	2476717	2477967	1	-1	RO3G_13031.1	RO3G_10153.1	625	329	92.1
##Scaffold_11	Scaffold_7	1051541	1077070	7	2118455	2140017	1	7		
1051541	1053088	2118455	2119687	-1	-1	RO3G_13078.1	RO3G_10020.1	287	242	64.46
1055117	1055825	2122455	2124043	1	1	RO3G_13080.1	RO3G_10022.1	224	165	70.3
1057973	1059248	2124465	2125731	1	1	RO3G_13082.1	RO3G_10023.1	559	333	97.3
1059818	1060344	2126301	2126825	1	1	RO3G_13083.1	RO3G_10024.1	221	139	98.56
1067739	1068272	2135249	2135774	1	1	RO3G_13087.1	RO3G_10028.1	300	155	92.9
1070052	1070958	2136755	2137687	-1	-1	RO3G_13088.1	RO3G_10030.1	186	287	42.86
1074801	1077070	2138601	2140017	-1	-1	RO3G_13089.1	RO3G_10031.1	470	335	71.34

##Scaffold_11	Scaffold_7	1099966	1144592	7	2210606	2234153	1	5		
1099966	1101185	2210606	2211915	-1	-1	RO3G_13097.1	RO3G_10055.1	568	336	92.86
1109666	1113621	2213341	2216911	-1	-1	RO3G_13100.1	RO3G_10056.1	1696	1072	76.87
1129970	1131967	2223968	2225736	1	1	RO3G_13109.1	RO3G_10059.1	730	561	72.37
1137472	1139778	2229823	2232101	1	1	RO3G_13112.1	RO3G_10063.1	1252	687	91.7
1143698	1144592	2233074	2234153	1	1	RO3G_13115.1	RO3G_10064.1	124	173	59.54

##Scaffold_11	Scaffold_8	70488	105426	8	564463	589638	-1	5		
104534	105426	564463	565240	-1	1	RO3G_12705.1	RO3G_10439.1	237	202	60.4
99754	101281	567051	568798	-1	1	RO3G_12703.1	RO3G_10440.1	710	539	72.91
95501	97828	574702	577051	1	-1	RO3G_12701.1	RO3G_10443.1	1173	659	86.34
83661	86114	580470	581483	1	-1	RO3G_12696.1	RO3G_10445.1	278	326	50.31
70488	71437	588659	589638	-1	1	RO3G_12690.1	RO3G_10448.1	200	272	45.59

##Scaffold_11	Scaffold_8	531478	577924	8	418629	488958	-1	8		
577171	577924	418629	419373	-1	1	RO3G_12899.1	RO3G_10390.1	187	152	63.82
575026	577107	419537	421758	1	-1	RO3G_12898.1	RO3G_10391.1	801	619	66.07
568351	570212	431233	433002	-1	1	RO3G_12896.1	RO3G_10395.1	786	526	72.05
553556	559848	451193	457439	-1	1	RO3G_12891.1	RO3G_10401.1	2891	1959	78.76
547449	549439	458934	460909	-1	1	RO3G_12888.1	RO3G_10403.1	1092	610	97.21
535833	538644	474745	477184	-1	1	RO3G_12884.1	RO3G_10406.1	886	661	77.31
532841	534853	483336	485261	-1	1	RO3G_12883.1	RO3G_10408.1	1023	536	95.9
531478	532477	487977	488958	-1	1	RO3G_12882.1	RO3G_10410.1	449	248	97.58

##Scaffold_11	Scaffold_8	691772	697662	8	387225	397690	1	3		
691772	692352	387225	387815	1	1	RO3G_12944.1	RO3G_10377.1	323	159	98.11
695570	696612	395750	396437	1	1	RO3G_12946.1	RO3G_10382.1	273	187	71.12
696945	697662	396790	397690	-1	-1	RO3G_12947.1	RO3G_10383.1	375	219	86.3

##Scaffold_12	Scaffold_10	200188	217971	10	691409	706852	-1	5		
217374	217971	691409	691672	1	-1	RO3G_13375.1	RO3G_12212.1	137	87	78.16
210400	211851	693559	695058	-1	1	RO3G_13373.1	RO3G_12214.1	518	344	79.36
208449	210072	695324	696896	-1	1	RO3G_13372.1	RO3G_12215.1	725	490	77.96
204914	207685	701979	704568	1	-1	RO3G_13371.1	RO3G_12218.1	1310	798	89.22
200188	200659	706144	706852	-1	1	RO3G_13369.1	RO3G_12219.1	95.5	89	52.81

##Scaffold_12	5Scaffold_12	66318	96738	5	1366150	1406956	-1	8		
95427	96738	1366150	1367799	1	-1	RO3G_13328.1	RO3G_07277.1	533	349	74.21
90231	92170	1369429	1371353	-1	1	RO3G_13326.1	RO3G_07278.1	265	172	76.74
87817	89540	1372611	1377228	-1	1	RO3G_13325.1	RO3G_07279.1	148	291	45.7
86079	87765	1377537	1379356	1	-1	RO3G_13324.1	RO3G_07280.1	344	321	63.24
81014	83061	1389965	1391077	-1	1	RO3G_13322.1	RO3G_07285.1	398	259	78.76
78173	80461	1396284	1398384	-1	1	RO3G_13321.1	RO3G_07288.1	1219	627	98.88
74784	76481	1401574	1403203	-1	1	RO3G_13320.1	RO3G_07291.1	620	545	62.39
66318	67259	1406205	1406956	1	-1	RO3G_13317.1	RO3G_07292.1	237	188	73.4

##Scaffold_12	Scaffold_5	594006	618878	5	1311107	1332861	-1	4		
618054	618878	1311107	1312559	1	-1	RO3G_13516.1	RO3G_07251.1	335	205	80.49
610830	613264	1316448	1319343	-1	1	RO3G_13513.1	RO3G_07254.1	865	750	70.53
602159	603096	1325734	1328585	-1	1	RO3G_13509.1	RO3G_07259.1	367	241	75.1
594006	596466	1330441	1332861	-1	1	RO3G_13506.1	RO3G_07261.1	1231	812	81.65

##Scaffold_12	Scaffold_9	910657	923587	9	335729	347289	-1	3		
921387	923587	335729	337927	-1	1	RO3G_13636.1	RO3G_11230.1	1148	673	99.85
917035	919225	339394	341466	1	-1	RO3G_13634.1	RO3G_11231.1	753	594	64.31
910657	913652	344142	347289	-1	1	RO3G_13632.1	RO3G_11233.1	1439	850	87.88

##Scaffold_13	Scaffold_10	367553	400596	10	1565236	1598400	-1	5		
398112	400596	1565236	1567832	1	-1	RO3G_14007.1	RO3G_12555.1	823	779	61.23
381164	382504	1573558	1574905	-1	1	RO3G_14002.1	RO3G_12558.1	754	375	100
375414	376617	1580956	1582178	-1	1	RO3G_13999.1	RO3G_12560.1	398	368	71.74
370923	372211	1586151	1587356	-1	1	RO3G_13996.1	RO3G_12562.1	408	354	66.1
367553	368071	1597881	1598400	-1	1	RO3G_13994.1	RO3G_12565.1	129	117	57.26

##Scaffold_13	Scaffold_11	71703	97727	11	1522316	1555103	1	4		
71703	73094	1522316	1523426	1	1	RO3G_13885.1	RO3G_13239.1	285	302	53.97
80562	82458	1526851	1528704	1	1	RO3G_13888.1	RO3G_13242.1	1063	548	99.27
85756	90382	1531811	1536534	-1	-1	RO3G_13890.1	RO3G_13244.1	2151	1476	79.61
96430	97727	1553598	1555103	-1	-1	RO3G_13893.1	RO3G_13253.1	529	385	72.47

##Scaffold_13	Scaffold_2	261856	287461	2	383892	414392	1	7		
261856	263303	383892	384846	-1	-1	RO3G_13946.1	RO3G_02363.1	457	242	97.11
266466	268547	396963	399220	1	1	RO3G_13948.1	RO3G_02368.1	409	636	42.45
268962	269339	400906	401273	1	1	RO3G_13949.1	RO3G_02369.1	189	106	83.02
269551	270911	401607	402046	1	1	RO3G_13950.1	RO3G_02370.1	116	93	73.12
271109	273334	402819	404638	-1	-1	RO3G_13951.1	RO3G_02371.1	672	501	72.85
278964	283340	406190	409908	-1	-1	RO3G_13953.1	RO3G_02372.1	1279	921	74.27
287065	287461	413976	414392	-1	-1	RO3G_13955.1	RO3G_02374.1	114	80	95

##Scaffold_13	Scaffold_2	702376	707130	2	127370	137055	1	3		
702376	703115	127370	128699	-1	-1	RO3G_14129.1	RO3G_02268.1	193	192	52.08
705623	706087	134786	135262	-1	-1	RO3G_14131.1	RO3G_02272.1	182	159	69.81
706662	707130	136198	137055	-1	-1	RO3G_14132.1	RO3G_02273.1	168	106	73.58
##Scaffold_13	Scaffold_2	1086083	1099872	2	595920	605377	1	3		
1086083	1088146	595920	597442	-1	-1	RO3G_14281.1	RO3G_02439.1	110	206	32.52
1092537	1095309	599997	603267	1	1	RO3G_14283.1	RO3G_02440.1	907	742	66.85
1099053	1099872	603498	605377	-1	-1	RO3G_14285.1	RO3G_02441.1	147	142	55.63
##Scaffold_13	Scaffold_2	1037443	1064434	2	916178	948158	1	8		
1037443	1038447	916178	917068	1	1	RO3G_14263.1	RO3G_02572.1	207	162	69.14
1040414	1041202	919271	920101	1	1	RO3G_14264.1	RO3G_02574.1	199	246	44.31
1044156	1044915	921543	922288	1	1	RO3G_14265.1	RO3G_02576.1	336	181	99.45
1045895	1047799	931037	931730	-1	-1	RO3G_14266.1	RO3G_02581.1	279	189	71.96
1050505	1051980	936831	938150	1	1	RO3G_14267.1	RO3G_02582.1	379	377	54.11
1054827	1055321	938733	939756	-1	-1	RO3G_14268.1	RO3G_02583.1	171	163	63.8
1060011	1061489	944465	945928	-1	-1	RO3G_14271.1	RO3G_02587.1	723	473	76.74
1062479	1064434	947654	948158	1	1	RO3G_14272.1	RO3G_02588.1	186	109	81.65
##Scaffold_13	Scaffold_2	1181444	1187667	2	307284	316977	1	3		
1181444	1182692	307284	308280	-1	-1	RO3G_14314.1	RO3G_02335.1	343	300	62
1183213	1185778	308814	311450	-1	-1	RO3G_14315.1	RO3G_02336.1	927	811	63.26
1186894	1187667	316239	316977	-1	-1	RO3G_14316.1	RO3G_02338.1	394	192	100
##Scaffold_13	Scaffold_6	502896	509705	6	1916606	1922587	1	3		
502896	504383	1916606	1917925	1	1	RO3G_14049.1	RO3G_08757.1	412	449	53.67
504726	506688	1918295	1920542	-1	-1	RO3G_14050.1	RO3G_08758.1	608	594	56.9
508176	509705	1921163	1922587	-1	-1	RO3G_14051.1	RO3G_08759.1	649	409	82.89
##Scaffold_14	Scaffold_4	831030	839224	4	83188	91387	1	3		
831030	832154	83188	83927	-1	-1	RO3G_14663.1	RO3G_05554.1	309	229	69.87
834742	836461	87075	88827	1	1	RO3G_14665.1	RO3G_05555.1	630	553	70.89
836610	839224	89038	91387	-1	-1	RO3G_14666.1	RO3G_05556.1	741	671	59.91
##Scaffold_14	Scaffold_6	357930	375162	6	2399857	2409943	-1	3		
374033	375162	2399857	2400995	-1	1	RO3G_14483.1	RO3G_08936.1	642	317	96.85
365178	366589	2406783	2408188	-1	1	RO3G_14478.1	RO3G_08940.1	795	435	95.86
357930	359547	2408713	2409943	1	-1	RO3G_14476.1	RO3G_08941.1	338	325	54.15
##Scaffold_15	Scaffold_4	869465	880759	4	1673661	1680776	1	3		
869465	871449	1673661	1675564	-1	-1	RO3G_15121.1	RO3G_06113.1	462	586	48.81
876095	876726	1679229	1679767	1	1	RO3G_15122.1	RO3G_06117.1	147	121	66.12
880271	880759	1680331	1680776	-1	-1	RO3G_15124.1	RO3G_06118.1	210	113	89.38
##Scaffold_15	Scaffold_6	794944	824904	6	49667	75799	1	7		
794944	795784	49667	50470	1	1	RO3G_15091.1	RO3G_08000.1	258	166	95.78
796148	797733	50670	52247	-1	-1	RO3G_15092.1	RO3G_08001.1	542	396	77.53
807256	807904	58323	58986	-1	-1	RO3G_15095.1	RO3G_08003.1	300	146	98.63
810800	814260	59618	61789	-1	-1	RO3G_15097.1	RO3G_08004.1	369	486	45.88
818653	819508	66007	67073	-1	-1	RO3G_15098.1	RO3G_08006.1	124	135	47.41
820188	820857	67963	69054	-1	-1	RO3G_15100.1	RO3G_08007.1	235	168	70.24
822675	824904	73434	75799	1	1	RO3G_15101.1	RO3G_08009.1	748	690	61.16
##Scaffold_15	Scaffold_6	1063985	1076231	6	136087	145311	1	3		
1063985	1065205	136087	137209	1	1	RO3G_15197.1	RO3G_08033.1	538	327	84.4
1065520	1066497	139777	142178	-1	-1	RO3G_15198.1	RO3G_08035.1	336	235	78.3
1074433	1076231	144350	145311	-1	-1	RO3G_15202.1	RO3G_08037.1	412	249	85.14
##Scaffold_16	Scaffold_12	752010	779865	12	1060783	1068451	1	3		
752010	753567	1060783	1062320	-1	-1	RO3G_15559.1	RO3G_13691.1	819	463	96.76
753778	755058	1062657	1063472	1	1	RO3G_15560.1	RO3G_13692.1	307	236	64.83
778800	779865	1067983	1068451	-1	-1	RO3G_15570.1	RO3G_13694.1	189	92	94.57
##Scaffold_16	Scaffold_21	270762	310599	21	60739	76616	1	3		
270762	273116	60739	63091	-1	-1	RO3G_15385.1	RO3G_16583.1	1344	768	85.03
289462	292463	67664	69856	-1	-1	RO3G_15392.1	RO3G_16585.1	722	513	74.07
309842	310599	75959	76616	-1	-1	RO3G_15398.1	RO3G_16588.1	179	95	91.58
##Scaffold_16	Scaffold_7	67254	79832	7	1818096	1833900	-1	3		
79058	79832	1818096	1818892	-1	1	RO3G_15303.1	RO3G_09898.1	327	181	96.13
73424	74818	1821281	1822141	1	-1	RO3G_15299.1	RO3G_09900.1	172	184	50
67254	68109	1832996	1833900	-1	1	RO3G_15294.1	RO3G_09904.1	431	268	85.07
##Scaffold_16	Scaffold_7	434523	446394	7	2594894	2621842	-1	4		
445524	446394	2594894	2595745	1	-1	RO3G_15452.1	RO3G_10200.1	400	220	93.18
443523	445351	2603304	2604495	-1	1	RO3G_15451.1	RO3G_10202.1	312	371	65.77

441913	443381	2605138	2606585	1	-1	RO3G_15450.1	RO3G_10204.1	697	442	80.77
434523	435730	2620715	2621842	1	-1	RO3G_15447.1	RO3G_10209.1	392	312	58.65
##Scaffold_17	Scaffold_18	77211	86539	18	160292	169370	1	3		
77211	78413	160292	162794	1	1	RO3G_15610.1	RO3G_15932.1	416	318	75.16
80176	82034	164164	166047	-1	-1	RO3G_15613.1	RO3G_15934.1	885	562	76.87
85567	86539	168590	169370	-1	-1	RO3G_15615.1	RO3G_15936.1	231	163	61.35
##Scaffold_17	Scaffold_18	487645	524778	18	417045	447388	-1	8		
524316	524778	417045	417496	-1	1	RO3G_15777.1	RO3G_16038.1	263	131	100
516877	517384	419871	420401	-1	1	RO3G_15775.1	RO3G_16041.1	234	133	91.73
512751	513912	428951	429932	1	-1	RO3G_15773.1	RO3G_16045.1	240	229	56.77
511676	512276	430288	431238	1	-1	RO3G_15772.1	RO3G_16046.1	201	152	63.16
509109	509594	431583	432104	1	-1	RO3G_15770.1	RO3G_16047.1	130	158	40.51
501097	502327	436624	437871	-1	1	RO3G_15767.1	RO3G_16049.1	693	373	87.13
498688	500768	438234	440160	1	-1	RO3G_15766.1	RO3G_16050.1	777	564	73.4
487645	490989	444055	447388	1	-1	RO3G_15763.1	RO3G_16052.1	1323	981	68.71
##Scaffold_17	Scaffold_2	411287	426135	2	4563272	4590613	1	4		
411287	412658	4563272	4564620	-1	-1	RO3G_15735.1	RO3G_04008.1	672	412	81.31
415638	417005	4568948	4570365	-1	-1	RO3G_15737.1	RO3G_04010.1	436	297	77.44
421519	423961	4579354	4581531	1	1	RO3G_15739.1	RO3G_04015.1	1236	702	89.6
424775	426135	4589233	4590613	1	1	RO3G_15740.1	RO3G_04020.1	768	432	97.22
##Scaffold_17	Scaffold_2	676907	702573	2	4423461	4462153	1	5		
676907	686778	4423461	4433793	1	1	RO3G_15840.1	RO3G_03964.1	2857	2613	58.52
690198	691550	4438019	4439535	1	1	RO3G_15843.1	RO3G_03967.1	399	451	51.88
692954	694627	4440568	4442797	1	1	RO3G_15845.1	RO3G_03968.1	595	513	57.7
699752	700291	4457720	4458414	-1	-1	RO3G_15848.1	RO3G_03975.1	229	137	82.48
701488	702573	4461116	4462153	1	1	RO3G_15849.1	RO3G_03977.1	149	351	33.33
##Scaffold_17	Scaffold_7	550793	574377	7	1179905	1197756	-1	6		
573253	574377	1179905	1180999	1	-1	RO3G_15797.1	RO3G_09645.1	402	241	100
571382	573097	1181276	1181920	1	-1	RO3G_15796.1	RO3G_09646.1	128	149	55.03
562193	563638	1185097	1187100	-1	1	RO3G_15792.1	RO3G_09649.1	343	294	65.65
558764	561513	1187256	1190005	1	-1	RO3G_15791.1	RO3G_09650.1	1667	843	100
557639	558541	1190228	1190977	1	-1	RO3G_15790.1	RO3G_09651.1	390	250	90.4
550793	551422	1197121	1197756	-1	1	RO3G_15787.1	RO3G_09653.1	362	192	97.92
##Scaffold_18	Scaffold_17	160292	169370	17	77211	86539	1	3		
160292	162794	77211	78413	1	1	RO3G_15932.1	RO3G_15610.1	416	318	75.16
164164	166047	80176	82034	-1	-1	RO3G_15934.1	RO3G_15613.1	885	562	76.87
168590	169370	85567	86539	-1	-1	RO3G_15936.1	RO3G_15615.1	231	163	61.35
##Scaffold_18	Scaffold_17	416307	440160	17	498688	525515	-1	8		
438234	440160	498688	500768	-1	1	RO3G_16050.1	RO3G_15766.1	777	564	73.4
436624	437871	501097	502327	1	-1	RO3G_16049.1	RO3G_15767.1	693	373	87.13
431583	432104	509109	509594	-1	1	RO3G_16047.1	RO3G_15770.1	130	158	40.51
430288	431238	511676	512276	-1	1	RO3G_16046.1	RO3G_15772.1	201	152	63.16
428951	429932	512751	513912	-1	1	RO3G_16045.1	RO3G_15773.1	240	229	56.77
419871	420401	516877	517384	1	-1	RO3G_16041.1	RO3G_15775.1	234	133	91.73
417045	417496	524316	524778	1	-1	RO3G_16038.1	RO3G_15777.1	263	131	100
416307	416549	525280	525515	-1	1	RO3G_16037.1	RO3G_15778.1	102	45	100
##Scaffold_18	Scaffold_4	317169	329021	4	2037373	2044821	1	4		
317169	317942	2037373	2038148	1	1	RO3G_15996.1	RO3G_06245.1	416	211	99.53
319599	320748	2041940	2042879	-1	-1	RO3G_15998.1	RO3G_06248.1	290	149	96.64
322370	323696	2043092	2044120	1	1	RO3G_15999.1	RO3G_06249.1	207	281	47.69
328425	329021	2044204	2044821	-1	-1	RO3G_16001.1	RO3G_06250.1	171	138	57.97
##Scaffold_18	Scaffold_4	518040	535479	4	1939885	1957699	-1	5		
534063	535479	1939885	1940415	1	-1	RO3G_16087.1	RO3G_06215.1	183	176	53.98
523622	524556	1944715	1945661	1	-1	RO3G_16083.1	RO3G_06217.1	408	230	89.13
521551	522709	1950113	1951221	-1	1	RO3G_16081.1	RO3G_06220.1	140	294	35.71
520221	521333	1951527	1952623	-1	1	RO3G_16080.1	RO3G_06221.1	398	260	92.31
518040	518567	1956857	1957699	1	-1	RO3G_16079.1	RO3G_06223.1	270	175	77.14
##Scaffold_18	Scaffold_8	273379	292995	8	319361	341149	-1	3		
290919	292995	319361	322163	-1	1	RO3G_15987.1	RO3G_10355.1	856	535	82.24
274725	275378	338202	338714	-1	1	RO3G_15979.1	RO3G_10362.1	221	172	70.35
273379	274464	339389	341149	1	-1	RO3G_15978.1	RO3G_10363.1	662	345	91.59
##Scaffold_19	1Scaffold_19	237962	246785	1	1347735	1359260	-1	3		
245057	246785	1347735	1349468	-1	1	RO3G_16215.1	RO3G_00533.1	876	458	97.6
239215	242170	1349741	1352787	-1	1	RO3G_16214.1	RO3G_00534.1	1506	938	79.42
237962	239061	1358172	1359260	1	-1	RO3G_16213.1	RO3G_00537.1	369	273	69.6
##Scaffold_19	1Scaffold_19	150947	160018	1	2475188	2485463	-1	4		
157429	160018	2475188	2476387	-1	1	RO3G_16180.1	RO3G_00978.1	332	375	58.4

155903	156742	2477159	2479279	-1	1	RO3G_16179.1	RO3G_00979.1	297	210	72.38
154590	155123	2479301	2479793	1	-1	RO3G_16178.1	RO3G_00980.1	133	165	63.64
150947	152996	2483746	2485463	1	-1	RO3G_16177.1	RO3G_00983.1	229	240	57.08
##Scaffold_19	Scaffold_1	169955	183434	1	2587587	2611792	-1	4		
182690	183434	2587587	2588491	-1	1	RO3G_16189.1	RO3G_01022.1	139	210	47.62
180637	181650	2589972	2590631	1	-1	RO3G_16188.1	RO3G_01024.1	157	173	66.47
179473	180287	2591063	2591886	1	-1	RO3G_16187.1	RO3G_01025.1	119	73	82.19
169955	174990	2608354	2611792	1	-1	RO3G_16184.1	RO3G_01031.1	1313	998	72.85
##Scaffold_19	Scaffold_1	101574	129254	1	3482785	3512047	-1	4		
117402	129254	3482785	3484068	-1	1	RO3G_16171.1	RO3G_01332.1	796	427	98.59
115207	116988	3487398	3489073	1	-1	RO3G_16170.1	RO3G_01334.1	665	373	82.84
104727	105591	3507656	3508159	-1	1	RO3G_16167.1	RO3G_01342.1	290	167	85.03
101574	104556	3509424	3512047	1	-1	RO3G_16166.1	RO3G_01343.1	1098	756	78.97
##Scaffold_19	Scaffold_1	145786	291783	1	4132265	4163483	1	3		
145786	147878	4132265	4136270	-1	-1	RO3G_16175.1	RO3G_01597.1	665	619	64.46
290348	291783	4144790	4146196	1	1	RO3G_16238.1	RO3G_01601.1	192	390	39.23
216238	217066	4162847	4163483	1	1	RO3G_16202.1	RO3G_01608.1	332	189	82.54
##Scaffold_19	Scaffold_10	443679	512628	10	213997	219958	1	3		
443679	444917	213997	215066	1	1	RO3G_16296.1	RO3G_12037.1	270	261	81.61
448167	448842	217292	217724	1	1	RO3G_16298.1	RO3G_12039.1	184	122	82.79
511843	512628	219169	219958	-1	-1	RO3G_16321.1	RO3G_12040.1	364	209	83.25
##Scaffold_19	Scaffold_2	323073	334511	2	3741192	3751537	-1	3		
333140	334511	3741192	3742604	1	-1	RO3G_16253.1	RO3G_03698.1	704	417	83.45
328440	329703	3745360	3746638	-1	1	RO3G_16251.1	RO3G_03700.1	407	399	55.14
323073	327373	3747181	3751537	-1	1	RO3G_16249.1	RO3G_03701.1	1984	1275	81.41
##Scaffold_19	Scaffold_2	393268	400900	2	3722926	3729285	1	3		
393268	394781	3722926	3724762	-1	-1	RO3G_16279.1	RO3G_03690.1	592	450	68.89
395579	397028	3725591	3726793	-1	-1	RO3G_16280.1	RO3G_03691.1	486	395	61.52
399432	400900	3728203	3729285	1	1	RO3G_16281.1	RO3G_03693.1	518	360	72.22
##Scaffold_2	Scaffold_10	650185	656949	10	853442	861327	1	3		
650185	651794	853442	855061	-1	-1	RO3G_02460.1	RO3G_12286.1	904	502	91.63
652381	655406	855373	858361	-1	-1	RO3G_02461.1	RO3G_12287.1	988	806	65.01
656433	656949	860803	861327	-1	-1	RO3G_02462.1	RO3G_12289.1	309	154	99.35
##Scaffold_2	Scaffold_11	3312999	3317753	11	1319791	1326508	1	3		
3312999	3314956	1319791	1321792	1	1	RO3G_03536.1	RO3G_13175.1	1102	626	88.02
3315374	3316657	1325085	1325680	1	1	RO3G_03537.1	RO3G_13177.1	193	101	86.14
3317204	3317753	1326012	1326508	1	1	RO3G_03538.1	RO3G_13178.1	283	147	96.6
##Scaffold_2	Scaffold_13	127370	137055	13	702376	707130	1	3		
127370	128699	702376	703115	-1	-1	RO3G_02268.1	RO3G_14129.1	193	192	52.08
134786	135262	705623	706087	-1	-1	RO3G_02272.1	RO3G_14131.1	182	159	69.81
136198	137055	706662	707130	-1	-1	RO3G_02273.1	RO3G_14132.1	168	106	73.58
##Scaffold_2	Scaffold_13	307284	316977	13	1181444	1187667	1	3		
307284	308280	1181444	1182692	-1	-1	RO3G_02335.1	RO3G_14314.1	343	300	62
308814	311450	1183213	1185778	-1	-1	RO3G_02336.1	RO3G_14315.1	927	811	63.26
316239	316977	1186894	1187667	-1	-1	RO3G_02338.1	RO3G_14316.1	394	192	100
##Scaffold_2	Scaffold_13	383892	414392	13	261856	287461	1	7		
383892	384846	261856	263303	-1	-1	RO3G_02363.1	RO3G_13946.1	457	242	97.11
396963	399220	266466	268547	1	1	RO3G_02368.1	RO3G_13948.1	409	636	42.45
400906	401273	268962	269339	1	1	RO3G_02369.1	RO3G_13949.1	189	106	83.02
401607	402046	269551	270911	1	1	RO3G_02370.1	RO3G_13950.1	116	93	73.12
402819	404638	271109	273334	-1	-1	RO3G_02371.1	RO3G_13951.1	672	501	72.85
406190	409908	278964	283340	-1	-1	RO3G_02372.1	RO3G_13953.1	1279	921	74.27
413976	414392	287065	287461	-1	-1	RO3G_02374.1	RO3G_13955.1	114	80	95
##Scaffold_2	Scaffold_13	595920	605377	13	1086083	1099872	1	3		
595920	597442	1086083	1088146	-1	-1	RO3G_02439.1	RO3G_14281.1	110	206	32.52
599997	603267	1092537	1095309	1	1	RO3G_02440.1	RO3G_14283.1	907	742	66.85
603498	605377	1099053	1099872	-1	-1	RO3G_02441.1	RO3G_14285.1	147	142	55.63
##Scaffold_2	Scaffold_13	916178	948158	13	1037443	1064434	1	8		
916178	917068	1037443	1038447	1	1	RO3G_02572.1	RO3G_14263.1	207	162	69.14
919271	920101	1040414	1041202	1	1	RO3G_02574.1	RO3G_14264.1	199	246	44.31
921543	922288	1044156	1044915	1	1	RO3G_02576.1	RO3G_14265.1	336	181	99.45
931037	931730	1045895	1047799	-1	-1	RO3G_02581.1	RO3G_14266.1	279	189	71.96
936831	938150	1050505	1051980	1	1	RO3G_02582.1	RO3G_14267.1	379	377	54.11
938733	939756	1054827	1055321	-1	-1	RO3G_02583.1	RO3G_14268.1	171	163	63.8
944465	945928	1060011	1061489	-1	-1	RO3G_02587.1	RO3G_14271.1	723	473	76.74

947654	948158	1062479	1064434	1	1	RO3G_02588.1	RO3G_14272.1	186	109	81.65
##Scaffold_2	Scaffold_17	4423461	4462153	17	676907	702573	1	5		
4423461	4433793	676907	686778	1	1	RO3G_03964.1	RO3G_15840.1	2857	2613	58.52
4438019	4439535	690198	691550	1	1	RO3G_03967.1	RO3G_15843.1	399	451	51.88
4440568	4442797	692954	694627	1	1	RO3G_03968.1	RO3G_15845.1	595	513	57.7
4457720	4458414	699752	700291	-1	-1	RO3G_03975.1	RO3G_15848.1	229	137	82.48
4461116	4462153	701488	702573	1	1	RO3G_03977.1	RO3G_15849.1	149	351	33.33
##Scaffold_2	Scaffold_17	4563272	4590613	17	411287	426135	1	4		
4563272	4564620	411287	412658	-1	-1	RO3G_04008.1	RO3G_15735.1	672	412	81.31
4568948	4570365	415638	417005	-1	-1	RO3G_04010.1	RO3G_15737.1	436	297	77.44
4579354	4581531	421519	423961	1	1	RO3G_04015.1	RO3G_15739.1	1236	702	89.6
4589233	4590613	424775	426135	1	1	RO3G_04020.1	RO3G_15740.1	768	432	97.22
##Scaffold_2	Scaffold_19	3737037	3746638	19	328440	339967	-1	3		
3745360	3746638	328440	329703	1	-1	RO3G_03700.1	RO3G_16251.1	407	399	55.14
3741192	3742604	333140	334511	-1	1	RO3G_03698.1	RO3G_16253.1	704	417	83.45
3737037	3740867	336054	339967	1	-1	RO3G_03697.1	RO3G_16255.1	1794	1055	88.63
##Scaffold_2	Scaffold_19	3722926	3729285	19	393268	400900	1	3		
3722926	3724762	393268	394781	-1	-1	RO3G_03690.1	RO3G_16279.1	592	450	68.89
3725591	3726793	395579	397028	-1	-1	RO3G_03691.1	RO3G_16280.1	486	395	61.52
3728203	3729285	399432	400900	1	1	RO3G_03693.1	RO3G_16281.1	518	360	72.22
##Scaffold_2	Scaffold_4	4960914	4971340	4	968018	976308	-1	3		
4969965	4971340	968018	968855	-1	1	RO3G_04164.1	RO3G_05872.1	346	236	71.19
4966411	4967519	973385	975018	-1	1	RO3G_04162.1	RO3G_05875.1	471	305	89.51
4960914	4961607	975610	976308	1	-1	RO3G_04159.1	RO3G_05876.1	270	150	96.67
##Scaffold_2	Scaffold_7	4745194	4755888	7	1579272	1589905	1	4		
4745194	4748425	1579272	1582504	-1	-1	RO3G_04076.1	RO3G_09793.1	1281	836	80.5
4750392	4751310	1582997	1583916	1	1	RO3G_04077.1	RO3G_09794.1	485	268	86.94
4753157	4753915	1584589	1586505	1	1	RO3G_04078.1	RO3G_09795.1	404	247	80.16
4754622	4755888	1588627	1589905	-1	-1	RO3G_04079.1	RO3G_09797.1	757	388	91.49
##Scaffold_2	Scaffold_8	2117189	2135611	8	987413	999965	1	3		
2130574	2131077	987413	987919	1	1	RO3G_03044.1	RO3G_10592.1	219	168	67.26
2134936	2135611	989674	990263	-1	-1	RO3G_03047.1	RO3G_10593.1	166	195	53.33
2117189	2118668	998449	999965	1	1	RO3G_03038.1	RO3G_10597.1	588	371	76.28
##Scaffold_2	Scaffold_8	2047323	2110144	8	1083214	1112456	1	7		
2104655	2107445	1083214	1084378	1	1	RO3G_03032.1	RO3G_10618.1	384	406	59.11
2107483	2108428	1085112	1085893	-1	-1	RO3G_03033.1	RO3G_10619.1	315	190	85.26
2108740	2110144	1086016	1088365	-1	-1	RO3G_03034.1	RO3G_10620.1	653	403	79.9
2047323	2048629	1095078	1096337	-1	-1	RO3G_03009.1	RO3G_10624.1	624	385	79.74
2050725	2051884	1100145	1101283	1	1	RO3G_03011.1	RO3G_10625.1	380	347	71.76
2056112	2058511	1105497	1108957	-1	-1	RO3G_03013.1	RO3G_10627.1	659	867	50.75
2065297	2066473	1111005	1112456	1	1	RO3G_03017.1	RO3G_10629.1	137	323	33.13
##Scaffold_2	Scaffold_8	2484375	2489033	8	1147362	1151616	-1	3		
2487753	2489033	1147362	1148644	-1	1	RO3G_03192.1	RO3G_10646.1	493	409	80.93
2486017	2487542	1149051	1150210	1	-1	RO3G_03191.1	RO3G_10647.1	578	314	93.63
2484375	2485570	1150397	1151616	-1	1	RO3G_03190.1	RO3G_10648.1	462	284	93.31
##Scaffold_2	Scaffold_8	4540444	4551445	8	705107	712714	1	3		
4540444	4541082	705107	705734	-1	-1	RO3G_04000.1	RO3G_10491.1	365	191	99.48
4547685	4548535	709779	710185	1	1	RO3G_04002.1	RO3G_10493.1	128	121	59.5
4548933	4551445	711103	712714	1	1	RO3G_04003.1	RO3G_10494.1	189	170	57.65
##Scaffold_2	Scaffold_9	1044713	1072209	9	1553436	1580121	1	3		
1044713	1046360	1553436	1555542	-1	-1	RO3G_02628.1	RO3G_11676.1	489	433	59.12
1052387	1053606	1561774	1564047	-1	-1	RO3G_02631.1	RO3G_11678.1	346	262	65.65
1070753	1072209	1578675	1580121	-1	-1	RO3G_02639.1	RO3G_11684.1	630	378	81.22
##Scaffold_2	Scaffold_9	1091132	1104633	9	1629355	1650133	1	3		
1091132	1094874	1629355	1633760	1	1	RO3G_02645.1	RO3G_11701.1	845	637	69.86
1100227	1101585	1642598	1643959	1	1	RO3G_02649.1	RO3G_11703.1	780	412	96.84
1102144	1104633	1647678	1650133	1	1	RO3G_02650.1	RO3G_11706.1	928	668	68.41
##Scaffold_2	Scaffold_9	1312519	1349373	9	1068482	1105756	1	7		
1312519	1314406	1068482	1070398	1	1	RO3G_02730.1	RO3G_11488.1	796	497	78.07
1314824	1318560	1070869	1074757	-1	-1	RO3G_02731.1	RO3G_11489.1	1204	1057	68.78
1320574	1322356	1079139	1080890	1	1	RO3G_02733.1	RO3G_11492.1	401	513	43.27
1323684	1326296	1089509	1090698	1	1	RO3G_02735.1	RO3G_11494.1	566	298	99.66
1327681	1330056	1092360	1094721	-1	-1	RO3G_02737.1	RO3G_11496.1	1203	771	83.53
1339712	1340081	1096771	1097133	1	1	RO3G_02740.1	RO3G_11497.1	200	99	96.97
1348339	1349373	1105154	1105756	-1	-1	RO3G_02743.1	RO3G_11500.1	234	194	55.67

##Scaffold_2	Scaffold_9	1450893	1471461	9	947923	960644	1	4			
1450893	1452411	947923	949409	1	1	RO3G_02786.1	RO3G_11439.1	704	472	73.52	
1456930	1458009	950998	952018	1	1	RO3G_02789.1	RO3G_11441.1	269	206	69.9	
1467356	1468496	953644	954771	-1	-1	RO3G_02794.1	RO3G_11443.1	590	340	84.12	
1469358	1471461	959092	960644	-1	-1	RO3G_02795.1	RO3G_11445.1	723	398	94.22	

##Scaffold_2	Scaffold_9	1505807	1515521	9	1493379	1504471	1	3		
1505807	1510416	1493379	1497629	-1	-1	RO3G_02810.1	RO3G_11651.1	1299	1168	65.41
1511284	1513230	1498598	1500973	-1	-1	RO3G_02811.1	RO3G_11652.1	817	658	73.56
1514433	1515521	1503407	1504471	1	1	RO3G_02812.1	RO3G_11653.1	446	292	73.97

##Scaffold_2	Scaffold_9	1794551	1805114	9	1338628	1352465	1	4		
1794551	1794901	1338628	1339020	-1	-1	RO3G_02913.1	RO3G_11593.1	155	124	69.35
1796110	1797180	1343750	1344831	1	1	RO3G_02914.1	RO3G_11596.1	562	322	86.96
1798502	1801178	1345680	1348345	-1	-1	RO3G_02915.1	RO3G_11597.1	1009	748	73.53
1803770	1805114	1350959	1352465	-1	-1	RO3G_02916.1	RO3G_11598.1	97.1	102	48.04

##Scaffold_2	Scaffold_9	1733597	1767308	9	1691613	1713832	1	5		
1733597	1737215	1691613	1694302	-1	-1	RO3G_02895.1	RO3G_11725.1	1144	823	74
1747510	1749054	1697511	1699271	-1	-1	RO3G_02898.1	RO3G_11728.1	520	360	76.39
1754198	1755912	1701007	1701804	-1	-1	RO3G_02899.1	RO3G_11729.1	283	248	63.71
1759268	1760651	1705933	1707249	-1	-1	RO3G_02901.1	RO3G_11731.1	342	275	58.91
1765914	1767308	1710465	1713832	1	1	RO3G_02903.1	RO3G_11732.1	633	496	70.56

##Scaffold_20	Scaffold_6	103035	136510	6	700869	729100	-1	7		
135696	136510	700869	701742	-1	1	RO3G_16411.1	RO3G_08253.1	281	263	69.58
133426	134692	706380	707642	-1	1	RO3G_16410.1	RO3G_08255.1	703	383	89.3
117450	118175	712898	714374	-1	1	RO3G_16403.1	RO3G_08257.1	393	198	95.96
113910	114352	715288	715727	-1	1	RO3G_16401.1	RO3G_08258.1	119	108	96.3
107283	108021	721416	722149	1	-1	RO3G_16397.1	RO3G_08261.1	292	163	93.25
105145	106833	725182	726870	-1	1	RO3G_16396.1	RO3G_08262.1	949	501	99.6
103035	104755	727392	729100	-1	1	RO3G_16395.1	RO3G_08263.1	723	467	71.73

##Scaffold_20	Scaffold_6	191612	206009	6	675360	689896	-1	4		
204120	206009	675360	676376	-1	1	RO3G_16439.1	RO3G_08241.1	213	154	85.71
202056	203600	676705	677720	1	-1	RO3G_16438.1	RO3G_08242.1	409	245	80.82
198253	199127	684246	685109	1	-1	RO3G_16436.1	RO3G_08245.1	262	195	76.92
191612	192786	688733	689896	1	-1	RO3G_16433.1	RO3G_08247.1	471	255	95.69

##Scaffold_20	Scaffold_6	254783	313526	6	602444	614096	1	3		
311843	313526	602444	603986	-1	-1	RO3G_16488.1	RO3G_08204.1	644	420	74.05
276665	277336	606140	606936	1	1	RO3G_16473.1	RO3G_08205.1	407	221	90.05
254783	256201	612705	614096	1	1	RO3G_16463.1	RO3G_08208.1	649	402	78.86

##Scaffold_21	Scaffold_16	60739	76616	16	270762	310599	1	3		
60739	63091	270762	273116	-1	-1	RO3G_16583.1	RO3G_15385.1	1344	768	85.03
67664	69856	289462	292463	-1	-1	RO3G_16585.1	RO3G_15392.1	722	513	74.07
75959	76616	309842	310599	-1	-1	RO3G_16588.1	RO3G_15398.1	179	95	91.58

##Scaffold_21	Scaffold_21	103863	111881	21	118413	126431	-1	3		
110491	111881	118413	119803	-1	1	RO3G_16604.1	RO3G_16608.1	697	389	100
107232	107714	122580	123062	-1	1	RO3G_16602.1	RO3G_16610.1	336	160	100
103863	104423	125871	126431	1	-1	RO3G_16601.1	RO3G_16611.1	351	186	100

##Scaffold_24	Scaffold_3	43683	51269	3	1872937	1887741	-1	3		
49502	51269	1872937	1874731	1	-1	RO3G_16941.1	RO3G_04913.1	969	512	94.92
46532	48841	1882543	1884852	1	-1	RO3G_16940.1	RO3G_04919.1	1425	769	97.53
43683	45789	1885522	1887741	1	-1	RO3G_16939.1	RO3G_04920.1	468	593	43.17

##Scaffold_24	Scaffold_3	76984	82209	3	1846411	1853447	-1	3		
80180	82209	1846411	1848217	1	-1	RO3G_16954.1	RO3G_04903.1	717	492	74.39
78736	79483	1850836	1851624	1	-1	RO3G_16953.1	RO3G_04904.1	209	156	67.95
76984	78443	1852515	1853447	-1	1	RO3G_16952.1	RO3G_04905.1	300	320	63.44

##Scaffold_24	Scaffold_6	175747	214194	6	2626427	2677335	-1	9		
213247	214194	2626427	2627000	1	-1	RO3G_17002.1	RO3G_09016.1	174	138	57.25
197028	202786	2641179	2646492	-1	1	RO3G_16999.1	RO3G_09020.1	887	1510	41.99
194278	196446	2649300	2650923	-1	1	RO3G_16998.1	RO3G_09022.1	471	372	65.59
192743	193672	2651250	2652179	1	-1	RO3G_16997.1	RO3G_09023.1	476	309	99.03
190476	190780	2656466	2656765	1	-1	RO3G_16996.1	RO3G_09025.1	150	82	90.24
186161	188668	2658432	2660888	-1	1	RO3G_16995.1	RO3G_09026.1	1105	702	75.07
182117	185302	2664468	2667379	-1	1	RO3G_16994.1	RO3G_09028.1	722	450	80.67
178834	181803	2667679	2670621	-1	1	RO3G_16993.1	RO3G_09029.1	695	629	65.98
175747	177517	2674064	2677335	-1	1	RO3G_16992.1	RO3G_09031.1	832	493	88.84

##Scaffold_25	Scaffold_4	87950	119952	4	379423	420222	-1	8		
118092	119952	379423	381287	-1	1	RO3G_17064.1	RO3G_05654.1	1129	571	99.82

112736	113519	385831	386375	-1	1	RO3G_17062.1	RO3G_05657.1	128	162	54.94
100159	101487	398878	399852	-1	1	RO3G_17056.1	RO3G_05663.1	232	157	70.06
97591	99843	400049	402301	1	-1	RO3G_17055.1	RO3G_05664.1	1289	751	86.82
93388	93997	411404	412648	-1	1	RO3G_17054.1	RO3G_05668.1	195	136	81.62
92285	92658	413653	414073	1	-1	RO3G_17053.1	RO3G_05669.1	141	98	68.37
89482	90126	418105	418758	-1	1	RO3G_17051.1	RO3G_05672.1	207	202	59.9
87950	88694	419235	420222	1	-1	RO3G_17050.1	RO3G_05673.1	159	166	57.83
##Scaffold_27	Scaffold_1	45872	51617	1	5640603	5647018	-1	3		
50723	51617	5640603	5641416	1	-1	RO3G_17175.1	RO3G_02193.1	266	180	70.56
49535	50416	5641498	5642580	1	-1	RO3G_17174.1	RO3G_02194.1	384	197	98.98
45872	47620	5645329	5647018	1	-1	RO3G_17173.1	RO3G_02197.1	797	428	92.99
##Scaffold_27	Scaffold_3	96256	103242	3	2232091	2238936	1	3		
96256	98218	2232091	2233287	1	1	RO3G_17193.1	RO3G_05062.1	348	284	63.38
98470	99020	2233954	2234468	-1	-1	RO3G_17194.1	RO3G_05063.1	125	56	98.21
101602	103242	2237283	2238936	1	1	RO3G_17196.1	RO3G_05065.1	855	453	99.34
##Scaffold_3	Scaffold_1	1739965	1759639	1	4322746	4339475	1	4		
1739965	1741648	4322746	4325046	1	1	RO3G_04858.1	RO3G_01670.1	689	427	78.22
1752045	1753673	4328025	4329658	-1	-1	RO3G_04863.1	RO3G_01672.1	954	485	99.18
1755449	1757983	4335837	4337834	1	1	RO3G_04864.1	RO3G_01675.1	858	465	90.32
1758159	1759639	4339019	4339475	-1	-1	RO3G_04865.1	RO3G_01677.1	229	133	83.46
##Scaffold_3	Scaffold_1	2148974	2156770	1	2277619	2284235	-1	3		
2155500	2156770	2277619	2278794	-1	1	RO3G_05029.1	RO3G_00896.1	379	360	63.61
2154560	2155278	2279002	2279729	1	-1	RO3G_05028.1	RO3G_00897.1	232	199	66.33
2148974	2150148	2282830	2284235	1	-1	RO3G_05025.1	RO3G_00898.1	236	217	52.07
##Scaffold_3	Scaffold_1	2058879	2085311	1	4348205	4365434	-1	4		
2081332	2085311	4348205	4350856	1	-1	RO3G_04999.1	RO3G_01681.1	1070	816	70.47
2076248	2076673	4358023	4358445	-1	1	RO3G_04997.1	RO3G_01685.1	150	139	79.14
2063883	2065638	4362564	4363763	-1	1	RO3G_04991.1	RO3G_01686.1	457	325	73.23
2058879	2059938	4364361	4365434	-1	1	RO3G_04988.1	RO3G_01687.1	622	320	98.75
##Scaffold_3	Scaffold_1	2023564	2044323	1	4377771	4387964	-1	3		
2041508	2044323	4377771	4380355	1	-1	RO3G_04980.1	RO3G_01693.1	1079	752	80.72
2029046	2029743	4383902	4384804	1	-1	RO3G_04976.1	RO3G_01694.1	216	185	61.08
2023564	2024574	4386984	4387964	1	-1	RO3G_04973.1	RO3G_01695.1	488	337	78.34
##Scaffold_3	Scaffold_1	2693474	2709438	1	2290435	2301669	1	3		
2693474	2694712	2290435	2291150	1	1	RO3G_05242.1	RO3G_00900.1	322	197	83.25
2697565	2699340	2293685	2295456	1	1	RO3G_05244.1	RO3G_00902.1	867	496	89.31
2708632	2709438	2301025	2301669	-1	-1	RO3G_05250.1	RO3G_00905.1	311	206	70.39
##Scaffold_3	1Scaffold_3	2822294	2826729	1	2236971	2244622	-1	3		
2825378	2826729	2236971	2238474	-1	1	RO3G_05291.1	RO3G_00881.1	733	403	87.1
2823434	2824187	2240573	2241323	-1	1	RO3G_05289.1	RO3G_00883.1	324	179	88.83
2822294	2823007	2243780	2244622	-1	1	RO3G_05288.1	RO3G_00885.1	387	202	97.03
##Scaffold_3	Scaffold_1	3027091	3052787	1	1674847	1699180	-1	7		
3051868	3052787	1674847	1675966	1	-1	RO3G_05379.1	RO3G_00664.1	226	192	70.83
3046924	3047825	1678789	1679370	1	-1	RO3G_05377.1	RO3G_00665.1	244	176	80.11
3042781	3043734	1681663	1682304	1	-1	RO3G_05375.1	RO3G_00666.1	301	217	76.5
3040122	3041439	1685798	1686784	-1	1	RO3G_05374.1	RO3G_00668.1	498	328	80.18
3038913	3039469	1692207	1693682	1	-1	RO3G_05373.1	RO3G_00670.1	171	95	81.05
3028752	3029256	1697065	1697462	-1	1	RO3G_05370.1	RO3G_00673.1	158	88	90.91
3027091	3028390	1697768	1699180	1	-1	RO3G_05369.1	RO3G_00674.1	279	424	54.01
##Scaffold_3	Scaffold_1	3316695	3333906	1	1834274	1857190	1	6		
3316695	3317390	1834274	1835377	-1	-1	RO3G_05476.1	RO3G_00729.1	251	365	43.84
3318802	3319503	1838611	1839346	1	1	RO3G_05477.1	RO3G_00731.1	283	149	95.3
3319846	3320141	1839413	1840165	-1	-1	RO3G_05478.1	RO3G_00732.1	117	84	64.29
3324668	3324967	1849249	1849545	1	1	RO3G_05480.1	RO3G_00735.1	161	97	81.44
3325339	3326298	1849981	1851664	-1	-1	RO3G_05481.1	RO3G_00736.1	449	291	84.19
3332302	3333906	1855837	1857190	1	1	RO3G_05484.1	RO3G_00739.1	646	340	97.06
##Scaffold_3	Scaffold_24	1871369	1884852	24	46532	55681	-1	3		
1882543	1884852	46532	48841	-1	1	RO3G_04919.1	RO3G_16940.1	1425	769	97.53
1872937	1874731	49502	51269	-1	1	RO3G_04913.1	RO3G_16941.1	969	512	94.92
1871369	1872855	54225	55681	1	-1	RO3G_04912.1	RO3G_16944.1	654	380	93.95
##Scaffold_3	Scaffold_24	1844345	1851624	24	78736	84074	-1	3		
1850836	1851624	78736	79483	-1	1	RO3G_04904.1	RO3G_16953.1	209	156	67.95
1846411	1848217	80180	82209	-1	1	RO3G_04903.1	RO3G_16954.1	717	492	74.39
1844345	1845596	83195	84074	1	-1	RO3G_04902.1	RO3G_16955.1	435	258	98.45
##Scaffold_3	Scaffold_27	2232091	2238936	27	96256	103242	1	3		

2232091	2233287	96256	98218	1	1	RO3G_05062.1	RO3G_17193.1	348	284	63.38
2233954	2234468	98470	99020	-1	-1	RO3G_05063.1	RO3G_17194.1	125	56	98.21
2237283	2238936	101602	103242	1	1	RO3G_05065.1	RO3G_17196.1	855	453	99.34
##Scaffold_3	Scaffold_3	1772380	1801297	3	173163	217959	1	5		
1772380	1774089	173163	175421	1	1	RO3G_04871.1	RO3G_04278.1	479	371	67.92
1783122	1784198	184486	185597	1	1	RO3G_04876.1	RO3G_04284.1	464	309	88.35
1786718	1788800	191162	193738	-1	-1	RO3G_04878.1	RO3G_04287.1	663	669	58.45
1793650	1795462	198961	200758	-1	-1	RO3G_04880.1	RO3G_04289.1	953	524	93.32
1800112	1801297	216550	217959	-1	-1	RO3G_04882.1	RO3G_04295.1	412	426	56.34
##Scaffold_3	Scaffold_4	496272	510132	4	140017	155891	-1	3		
507429	510132	140017	142228	1	-1	RO3G_04406.1	RO3G_05579.1	721	662	56.65
502610	504508	147466	149132	1	-1	RO3G_04404.1	RO3G_05582.1	378	392	65.82
496272	499237	152557	155891	1	-1	RO3G_04403.1	RO3G_05583.1	1318	864	85.76
##Scaffold_3	Scaffold_5	1453173	1489060	5	167202	199282	-1	5		
1486022	1489060	167202	169268	1	-1	RO3G_04754.1	RO3G_06796.1	1080	634	89.75
1483474	1484143	172607	173171	-1	1	RO3G_04753.1	RO3G_06798.1	167	193	48.7
1475629	1481888	174028	178182	1	-1	RO3G_04752.1	RO3G_06799.1	1533	1249	68.94
1471933	1473400	179697	181911	1	-1	RO3G_04751.1	RO3G_06801.1	343	250	78.8
1453173	1456259	196028	199282	1	-1	RO3G_04747.1	RO3G_06809.1	988	968	63.43
##Scaffold_3	Scaffold_5	1642932	1671669	5	59583	90938	-1	5		
1670747	1671669	59583	60505	-1	1	RO3G_04826.1	RO3G_06756.1	347	286	72.38
1669296	1669861	61504	61873	-1	1	RO3G_04825.1	RO3G_06757.1	185	101	92.08
1662733	1665134	65462	67809	-1	1	RO3G_04824.1	RO3G_06759.1	1145	771	76.01
1645111	1649276	85065	88872	1	-1	RO3G_04817.1	RO3G_06767.1	1065	1067	61.2
1642932	1643825	90132	90938	1	-1	RO3G_04815.1	RO3G_06768.1	421	264	71.59
##Scaffold_3	Scaffold_5	2512567	2550158	5	578830	606122	1	5		
2512567	2512809	578830	579135	-1	-1	RO3G_05179.1	RO3G_06969.1	127	98	65.31
2526212	2527643	581693	583043	1	1	RO3G_05185.1	RO3G_06971.1	389	355	55.77
2532956	2533752	598251	598826	1	1	RO3G_05188.1	RO3G_06978.1	228	122	95.08
2543864	2544585	602966	603688	-1	-1	RO3G_05193.1	RO3G_06981.1	391	203	98.52
2548933	2550158	604885	606122	1	1	RO3G_05195.1	RO3G_06982.1	659	352	92.05
##Scaffold_3	Scaffold_5	2630483	2640732	5	677654	685720	1	3		
2630483	2632611	677654	679063	1	1	RO3G_05223.1	RO3G_07014.1	365	340	56.18
2633034	2633807	679981	680844	-1	-1	RO3G_05224.1	RO3G_07015.1	160	145	60
2640115	2640732	685118	685720	-1	-1	RO3G_05225.1	RO3G_07016.1	169	224	46.88
##Scaffold_3	Scaffold_7	285085	301470	7	292377	307378	-1	5		
300900	301470	292377	292950	-1	1	RO3G_04330.1	RO3G_09303.1	125	156	65.38
296040	296389	297152	297511	1	-1	RO3G_04326.1	RO3G_09306.1	141	101	91.09
288974	291845	300702	303036	-1	1	RO3G_04324.1	RO3G_09308.1	821	678	78.91
286376	288723	303766	306127	-1	1	RO3G_04323.1	RO3G_09309.1	1295	723	92.53
285085	286054	306458	307378	1	-1	RO3G_04322.1	RO3G_09310.1	204	208	68.27
##Scaffold_3	Scaffold_8	1943152	1958910	8	2206482	2225014	1	4		
1943152	1943856	2206482	2207186	-1	-1	RO3G_04941.1	RO3G_11031.1	328	234	82.91
1951832	1953437	2213607	2215084	-1	-1	RO3G_04947.1	RO3G_11034.1	521	495	61.01
1957186	1957739	2223782	2224338	-1	-1	RO3G_04949.1	RO3G_11037.1	301	148	97.3
1957907	1958910	2224654	2225014	1	1	RO3G_04950.1	RO3G_11038.1	152	68	97.06
##Scaffold_3	Scaffold_8	2305595	2320106	8	2060028	2091501	-1	5		
2318215	2320106	2060028	2065387	-1	1	RO3G_05103.1	RO3G_10973.1	493	335	88.06
2315079	2317361	2066247	2068511	1	-1	RO3G_05101.1	RO3G_10974.1	1061	703	73.83
2313367	2314656	2072849	2074143	1	-1	RO3G_05100.1	RO3G_10977.1	513	330	79.39
2311510	2312828	2074644	2075970	-1	1	RO3G_05099.1	RO3G_10978.1	383	269	75.46
2305595	2305883	2091215	2091501	-1	1	RO3G_05096.1	RO3G_10984.1	96.7	76	55.26
##Scaffold_3	Scaffold_8	2278642	2298135	8	2118018	2138198	-1	7		
2297561	2298135	2118018	2118708	1	-1	RO3G_05092.1	RO3G_10994.1	172	126	61.9
2288139	2289094	2122676	2123600	1	-1	RO3G_05089.1	RO3G_10997.1	445	220	97.27
2287057	2287896	2124201	2124653	-1	1	RO3G_05088.1	RO3G_10998.1	265	150	98
2283244	2284621	2127129	2128647	1	-1	RO3G_05087.1	RO3G_11000.1	566	345	81.74
2281486	2282583	2132669	2133769	-1	1	RO3G_05086.1	RO3G_11003.1	566	330	91.52
2280030	2281131	2135288	2136388	-1	1	RO3G_05085.1	RO3G_11004.1	601	310	96.77
2278642	2279184	2137494	2138198	-1	1	RO3G_05084.1	RO3G_11005.1	113	164	45.73
##Scaffold_3	Scaffold_8	2447901	2488189	8	1979675	2005287	-1	5		
2487332	2488189	1979675	1980622	-1	1	RO3G_05165.1	RO3G_10940.1	139	143	60.84
2474494	2475747	1986310	1987236	-1	1	RO3G_05159.1	RO3G_10942.1	327	317	59.94
2462479	2463537	1987581	1988653	-1	1	RO3G_05156.1	RO3G_10943.1	476	245	95.1
2452849	2453207	1997333	1997672	1	-1	RO3G_05152.1	RO3G_10949.1	150	75	98.67
2447901	2449025	2004163	2005287	-1	1	RO3G_05150.1	RO3G_10952.1	610	374	77.54
##Scaffold_3	Scaffold_8	2405983	2414111	8	2274238	2281520	-1	3		

2413581	2414111	2274238	2274798	1	-1	RO3G_05140.1	RO3G_11057.1	231	142	88.73
2407668	2408866	2279269	2279958	1	-1	RO3G_05137.1	RO3G_11061.1	192	145	73.79
2405983	2407257	2280266	2281520	1	-1	RO3G_05136.1	RO3G_11062.1	531	300	84.67
##Scaffold_3	Scaffold_9	622808	639056	9	128981	150483	-1	4		
638340	639056	128981	129689	-1	1	RO3G_04456.1	RO3G_11156.1	360	180	98.89
629178	631452	135927	138289	-1	1	RO3G_04453.1	RO3G_11158.1	820	644	64.29
625467	627019	145804	147370	1	-1	RO3G_04451.1	RO3G_11162.1	832	429	99.53
622808	623856	149092	150483	1	-1	RO3G_04450.1	RO3G_11163.1	401	346	60.98
##Scaffold_4	Scaffold_1	164669	209951	1	2972202	3006213	1	7		
164669	165459	2972202	2972976	-1	-1	RO3G_05587.1	RO3G_01173.1	135	98	72.45
168658	172910	2973788	2977012	1	1	RO3G_05589.1	RO3G_01174.1	870	693	66.96
173228	175047	2977942	2979738	-1	-1	RO3G_05590.1	RO3G_01175.1	659	518	74.9
177360	185781	2980190	2987338	-1	-1	RO3G_05593.1	RO3G_01176.1	1911	1562	68.63
191623	193127	2994842	2995714	1	1	RO3G_05595.1	RO3G_01181.1	332	226	74.78
205978	207794	3001057	3002940	-1	-1	RO3G_05600.1	RO3G_01184.1	605	522	60.73
208364	209951	3004847	3006213	-1	-1	RO3G_05601.1	RO3G_01185.1	329	509	52.85
##Scaffold_4	Scaffold_1	1497289	1516902	1	62010	84064	1	4		
1497289	1498647	62010	63496	-1	-1	RO3G_06049.1	RO3G_00024.1	637	338	93.2
1498815	1501111	63914	65025	-1	-1	RO3G_06050.1	RO3G_00025.1	560	348	82.47
1510755	1512183	78434	79817	1	1	RO3G_06055.1	RO3G_00031.1	542	439	59
1515966	1516902	83378	84064	1	1	RO3G_06056.1	RO3G_00034.1	182	145	62.76
##Scaffold_4	Scaffold_1	1522902	1539997	1	100036	112457	-1	3		
1539669	1539997	100036	100341	1	-1	RO3G_06064.1	RO3G_00043.1	108	106	55.66
1535524	1538325	104076	105388	-1	1	RO3G_06063.1	RO3G_00045.1	411	375	58.13
1522902	1524173	111357	112457	1	-1	RO3G_06058.1	RO3G_00049.1	300	183	76.5
##Scaffold_4	Scaffold_1	1833814	1854876	1	1070409	1088933	1	4		
1833814	1834281	1070409	1070876	1	1	RO3G_06177.1	RO3G_00430.1	246	155	96.77
1847158	1848769	1081320	1082875	1	1	RO3G_06181.1	RO3G_00434.1	748	472	77.33
1849807	1850490	1083195	1083873	1	1	RO3G_06182.1	RO3G_00435.1	189	187	68.45
1853380	1854876	1087478	1088933	-1	-1	RO3G_06184.1	RO3G_00437.1	516	348	70.69
##Scaffold_4	Scaffold_1	2115360	2151664	1	1168571	1179342	1	4		
2138900	2140152	1168571	1169602	1	1	RO3G_06287.1	RO3G_00461.1	294	301	56.81
2142082	2144478	1170026	1172275	-1	-1	RO3G_06289.1	RO3G_00462.1	1031	758	69.66
2150054	2151664	1174907	1176522	1	1	RO3G_06292.1	RO3G_00463.1	991	520	91.54
2115360	2118009	1176908	1179342	-1	-1	RO3G_06279.1	RO3G_00464.1	869	787	72.05
##Scaffold_4	Scaffold_14	83188	91387	14	831030	839224	1	3		
83188	83927	831030	832154	-1	-1	RO3G_05554.1	RO3G_14663.1	309	229	69.87
87075	88827	834742	836461	1	1	RO3G_05555.1	RO3G_14665.1	630	553	70.89
89038	91387	836610	839224	-1	-1	RO3G_05556.1	RO3G_14666.1	741	671	59.91
##Scaffold_4	Scaffold_15	1673661	1680776	15	869465	880759	1	3		
1673661	1675564	869465	871449	-1	-1	RO3G_06113.1	RO3G_15121.1	462	586	48.81
1679229	1679767	876095	876726	1	1	RO3G_06117.1	RO3G_15122.1	147	121	66.12
1680331	1680776	880271	880759	-1	-1	RO3G_06118.1	RO3G_15124.1	210	113	89.38
##Scaffold_4	Scaffold_18	1935966	1952623	18	520221	541847	-1	5		
1951527	1952623	520221	521333	1	-1	RO3G_06221.1	RO3G_16080.1	398	260	92.31
1950113	1951221	521551	522709	1	-1	RO3G_06220.1	RO3G_16081.1	140	294	35.71
1944715	1945661	523622	524556	-1	1	RO3G_06217.1	RO3G_16083.1	408	230	89.13
1939885	1940415	534063	535479	-1	1	RO3G_06215.1	RO3G_16087.1	183	176	53.98
1935966	1936822	540990	541847	1	-1	RO3G_06213.1	RO3G_16089.1	401	267	77.9
##Scaffold_4	Scaffold_18	2037373	2044821	18	317169	329021	1	4		
2037373	2038148	317169	317942	1	1	RO3G_06245.1	RO3G_15996.1	416	211	99.53
2041940	2042879	319599	320748	-1	-1	RO3G_06248.1	RO3G_15998.1	290	149	96.64
2043092	2044120	322370	323696	1	1	RO3G_06249.1	RO3G_15999.1	207	281	47.69
2044204	2044821	328425	329021	-1	-1	RO3G_06250.1	RO3G_16001.1	171	138	57.97
##Scaffold_4	Scaffold_2	966238	975018	2	4966411	4972744	-1	3		
973385	975018	4966411	4967519	1	-1	RO3G_05875.1	RO3G_04162.1	471	305	89.51
968018	968855	4969965	4971340	1	-1	RO3G_05872.1	RO3G_04164.1	346	236	71.19
966238	967488	4971818	4972744	1	-1	RO3G_05871.1	RO3G_04165.1	284	315	48.89
##Scaffold_4	Scaffold_25	372363	418758	25	89482	127371	-1	8		
418105	418758	89482	90126	1	-1	RO3G_05672.1	RO3G_17051.1	207	202	59.9
413653	414073	92285	92658	-1	1	RO3G_05669.1	RO3G_17053.1	141	98	68.37
411404	412648	93388	93997	1	-1	RO3G_05668.1	RO3G_17054.1	195	136	81.62
400049	402301	97591	99843	-1	1	RO3G_05664.1	RO3G_17055.1	1289	751	86.82
398878	399852	100159	101487	1	-1	RO3G_05663.1	RO3G_17056.1	232	157	70.06
385831	386375	112736	113519	1	-1	RO3G_05657.1	RO3G_17062.1	128	162	54.94
379423	381287	118092	119952	1	-1	RO3G_05654.1	RO3G_17064.1	1129	571	99.82
372363	373773	125958	127371	1	-1	RO3G_05651.1	RO3G_17067.1	668	429	74.83

##Scaffold_4	Scaffold_3	134947	149132	3	502610	512992	-1	3		
147466	149132	502610	504508	-1	1	RO3G_05582.1	RO3G_04404.1	378	392	65.82
140017	142228	507429	510132	-1	1	RO3G_05579.1	RO3G_04406.1	721	662	56.65
134947	135595	512756	512992	1	-1	RO3G_05576.1	RO3G_04408.1	139	77	87.01

##Scaffold_4	Scaffold_5	2848725	2856699	5	2293793	2300988	-1	3		
2856197	2856699	2293793	2294825	-1	1	RO3G_06559.1	RO3G_07641.1	141	153	56.86
2854244	2855878	2294990	2296932	-1	1	RO3G_06558.1	RO3G_07642.1	523	374	68.45
2848725	2850579	2299015	2300988	-1	1	RO3G_06556.1	RO3G_07643.1	324	211	76.78

##Scaffold_4	Scaffold_7	2237170	2259379	7	2087308	2113149	1	5		
2237170	2238048	2087308	2088384	-1	-1	RO3G_06324.1	RO3G_10005.1	437	258	80.62
2248149	2250337	2092357	2093959	-1	-1	RO3G_06329.1	RO3G_10007.1	542	382	78.8
2251485	2252431	2098919	2099682	-1	-1	RO3G_06330.1	RO3G_10009.1	239	167	73.65
2255946	2256559	2106809	2107377	-1	-1	RO3G_06332.1	RO3G_10014.1	214	106	100
2258902	2259379	2112741	2113149	1	1	RO3G_06334.1	RO3G_10017.1	150	138	68.84

##Scaffold_4	Scaffold_7	2203419	2225136	7	2243731	2269518	-1	5		
2222477	2225136	2243731	2245458	1	-1	RO3G_06320.1	RO3G_10068.1	423	459	55.77
2213649	2214453	2253998	2254792	-1	1	RO3G_06318.1	RO3G_10072.1	320	176	83.52
2210621	2213295	2255156	2257614	1	-1	RO3G_06317.1	RO3G_10073.1	576	700	51.43
2206181	2206836	2264274	2265050	1	-1	RO3G_06314.1	RO3G_10076.1	197	126	79.37
2203419	2204318	2268500	2269518	1	-1	RO3G_06313.1	RO3G_10078.1	281	211	61.61

##Scaffold_5	Scaffold_1	284891	294155	1	4846119	4857658	1	3		
284891	291199	4846119	4853721	1	1	RO3G_06842.1	RO3G_01878.1	2300	2107	61.32
292625	293021	4853981	4854441	1	1	RO3G_06844.1	RO3G_01879.1	135	108	62.04
293315	294155	4856675	4857658	1	1	RO3G_06845.1	RO3G_01881.1	519	264	94.32

##Scaffold_5	Scaffold_1	1130584	1199115	1	1898080	1930471	-1	5		
1196136	1199115	1898080	1900752	-1	1	RO3G_07203.1	RO3G_00753.1	944	840	69.05
1176735	1177506	1912091	1912849	-1	1	RO3G_07197.1	RO3G_00757.1	352	217	78.8
1174817	1176298	1918641	1919072	-1	1	RO3G_07196.1	RO3G_00759.1	144	101	67.33
1133390	1136327	1922615	1926049	-1	1	RO3G_07178.1	RO3G_00761.1	862	968	55.17
1130584	1132462	1928298	1930471	1	-1	RO3G_07176.1	RO3G_00763.1	362	498	51

##Scaffold_5	Scaffold_1	1095290	1109546	1	2401358	2416134	-1	3		
1105862	1109546	2401358	2404385	1	-1	RO3G_07164.1	RO3G_00949.1	216	136	80.15
1102946	1105346	2405151	2406846	1	-1	RO3G_07163.1	RO3G_00950.1	893	505	87.33
1095290	1099356	2411949	2416134	-1	1	RO3G_07161.1	RO3G_00951.1	1709	1242	76.25

##Scaffold_5	Scaffold_1	1154303	1167443	1	2425616	2437262	-1	3		
1166410	1167443	2425616	2426098	-1	1	RO3G_07192.1	RO3G_00957.1	114	85	67.06
1163181	1164072	2430998	2431836	1	-1	RO3G_07189.1	RO3G_00960.1	235	292	47.6
1154303	1155938	2435372	2437262	-1	1	RO3G_07187.1	RO3G_00961.1	612	436	69.04

##Scaffold_5	Scaffold_1	1236817	1245436	1	2620445	2637336	-1	3		
1244986	1245436	2620445	2620915	1	-1	RO3G_07224.1	RO3G_01034.1	226	111	97.3
1241736	1242539	2623035	2623817	-1	1	RO3G_07222.1	RO3G_01036.1	237	117	98.29
1236817	1238379	2636110	2637336	1	-1	RO3G_07219.1	RO3G_01042.1	677	371	95.96

##Scaffold_5	Scaffold_10	2751927	2777449	10	388049	417146	-1	4		
2776151	2777449	388049	389359	1	-1	RO3G_07780.1	RO3G_12094.1	455	437	68.65
2767509	2769452	393939	396779	1	-1	RO3G_07777.1	RO3G_12097.1	373	639	40.06
2760005	2760738	405241	405997	1	-1	RO3G_07776.1	RO3G_12100.1	175	105	77.14
2751927	2753802	415277	417146	1	-1	RO3G_07774.1	RO3G_12103.1	673	513	67.06

##Scaffold_5	Scaffold_10	3064920	3086882	10	1668749	1699510	-1	5		
3084734	3086882	1668749	1671536	-1	1	RO3G_07899.1	RO3G_12596.1	755	546	79.49
3076010	3078755	1687967	1690641	1	-1	RO3G_07895.1	RO3G_12602.1	1468	813	98.52
3073085	3073411	1691835	1692419	-1	1	RO3G_07893.1	RO3G_12603.1	114	91	61.54
3069201	3069577	1693497	1693883	1	-1	RO3G_07891.1	RO3G_12604.1	175	86	100
3064920	3067220	1698411	1699510	-1	1	RO3G_07890.1	RO3G_12607.1	394	268	74.25

##Scaffold_5	Scaffold_12	1363652	1403203	12	74784	104819	-1	8		
1401574	1403203	74784	76481	1	-1	RO3G_07291.1	RO3G_13320.1	620	545	62.39
1396284	1398384	78173	80461	1	-1	RO3G_07288.1	RO3G_13321.1	1219	627	98.88
1389965	1391077	81014	83061	1	-1	RO3G_07285.1	RO3G_13322.1	398	259	78.76
1377537	1379356	86079	87765	-1	1	RO3G_07280.1	RO3G_13324.1	344	321	63.24
1372611	1377228	87817	89540	1	-1	RO3G_07279.1	RO3G_13325.1	148	291	45.7
1369429	1371353	90231	92170	1	-1	RO3G_07278.1	RO3G_13326.1	265	172	76.74
1366150	1367799	95427	96738	-1	1	RO3G_07277.1	RO3G_13328.1	533	349	74.21
1363652	1364640	103860	104819	-1	1	RO3G_07276.1	RO3G_13332.1	313	173	87.86

##Scaffold_5	Scaffold_12	1307272	1328585	12	602159	626285	-1	4		
1325734	1328585	602159	603096	1	-1	RO3G_07259.1	RO3G_13509.1	367	241	75.1
1316448	1319343	610830	613264	1	-1	RO3G_07254.1	RO3G_13513.1	865	750	70.53

1311107	1312559	618054	618878	-1	1	RO3G_07251.1	RO3G_13516.1	335	205	80.49
1307272	1307896	625658	626285	-1	1	RO3G_07249.1	RO3G_13520.1	262	136	100
##Scaffold_5	Scaffold_12	1513665	1578057	12	319112	345455	-1	3		
1516153	1517438	319112	320339	1	-1	RO3G_07341.1	RO3G_13412.1	536	414	69.08
1513665	1515173	333881	335580	1	-1	RO3G_07340.1	RO3G_13420.1	533	495	58.99
1577638	1578057	344769	345455	-1	1	RO3G_07365.1	RO3G_13424.1	149	144	46.53
##Scaffold_5	Scaffold_3	57324	88872	3	1645111	1674000	-1	5		
85065	88872	1645111	1649276	-1	1	RO3G_06767.1	RO3G_04817.1	1065	1067	61.2
65462	67809	1662733	1665134	1	-1	RO3G_06759.1	RO3G_04824.1	1145	771	76.01
61504	61873	1669296	1669861	1	-1	RO3G_06757.1	RO3G_04825.1	185	101	92.08
59583	60505	1670747	1671669	1	-1	RO3G_06756.1	RO3G_04826.1	347	286	72.38
57324	58831	1672120	1674000	-1	1	RO3G_06755.1	RO3G_04827.1	507	492	54.88
##Scaffold_5	Scaffold_3	166685	181911	3	1471933	1490365	-1	5		
179697	181911	1471933	1473400	-1	1	RO3G_06801.1	RO3G_04751.1	343	250	78.8
174028	178182	1475629	1481888	-1	1	RO3G_06799.1	RO3G_04752.1	1533	1249	68.94
172607	173171	1483474	1484143	1	-1	RO3G_06798.1	RO3G_04753.1	167	193	48.7
167202	169268	1486022	1489060	-1	1	RO3G_06796.1	RO3G_04754.1	1080	634	89.75
166685	167107	1489946	1490365	1	-1	RO3G_06795.1	RO3G_04755.1	171	142	61.27
##Scaffold_5	Scaffold_3	578830	606122	3	2512567	2550158	1	5		
578830	579135	2512567	2512809	-1	-1	RO3G_06969.1	RO3G_05179.1	127	98	65.31
581693	583043	2526212	2527643	1	1	RO3G_06971.1	RO3G_05185.1	389	355	55.77
598251	598826	2532956	2533752	1	1	RO3G_06978.1	RO3G_05188.1	228	122	95.08
602966	603688	2543864	2544585	-1	-1	RO3G_06981.1	RO3G_05193.1	391	203	98.52
604885	606122	2548933	2550158	1	1	RO3G_06982.1	RO3G_05195.1	659	352	92.05
##Scaffold_5	Scaffold_3	677654	685720	3	2630483	2640732	1	3		
677654	679063	2630483	2632611	1	1	RO3G_07014.1	RO3G_05223.1	365	340	56.18
679981	680844	2633034	2633807	-1	-1	RO3G_07015.1	RO3G_05224.1	160	145	60
685118	685720	2640115	2640732	-1	-1	RO3G_07016.1	RO3G_05225.1	169	224	46.88
##Scaffold_5	Scaffold_4	2290174	2296932	4	2854244	2864235	-1	3		
2294990	2296932	2854244	2855878	1	-1	RO3G_07642.1	RO3G_06558.1	523	374	68.45
2293793	2294825	2856197	2856699	1	-1	RO3G_07641.1	RO3G_06559.1	141	153	56.86
2290174	2291690	2862851	2864235	-1	1	RO3G_07640.1	RO3G_06561.1	657	387	85.53
##Scaffold_5	Scaffold_5	2846188	2870410	5	3161820	3185263	1	4		
2846188	2848262	3161820	3163734	-1	-1	RO3G_07806.1	RO3G_07933.1	405	287	72.82
2854996	2856716	3164982	3166077	-1	-1	RO3G_07809.1	RO3G_07934.1	152	213	48.83
2867737	2869493	3178747	3180738	1	1	RO3G_07815.1	RO3G_07939.1	470	568	55.99
2869806	2870410	3184697	3185263	1	1	RO3G_07816.1	RO3G_07941.1	286	152	87.5
##Scaffold_5	Scaffold_5	3109185	3116837	5	2825913	2834321	-1	3		
3116268	3116837	2825913	2826503	1	-1	RO3G_07915.1	RO3G_07799.1	320	197	85.28
3113736	3114513	2829041	2829930	1	-1	RO3G_07914.1	RO3G_07801.1	151	210	45.71
3109185	3110802	2832624	2834321	1	-1	RO3G_07912.1	RO3G_07802.1	655	377	86.21
##Scaffold_5	Scaffold_5	3186839	3190713	5	2872054	2875890	1	3		
3186839	3187645	2872054	2872880	-1	-1	RO3G_07942.1	RO3G_07818.1	379	249	71.49
3188057	3189097	2873218	2874275	-1	-1	RO3G_07943.1	RO3G_07819.1	325	342	69.59
3189675	3190713	2874852	2875890	-1	-1	RO3G_07944.1	RO3G_07820.1	620	313	96.49
##Scaffold_6	Scaffold_1	399814	429722	1	394357	414176	1	4		
399814	400037	394357	395425	1	1	RO3G_08130.1	RO3G_00161.1	103	58	77.59
406132	406827	400698	401562	1	1	RO3G_08133.1	RO3G_00163.1	202	183	60.66
421528	422334	410089	410917	1	1	RO3G_08137.1	RO3G_00166.1	236	160	82.5
426908	429722	411492	414176	1	1	RO3G_08138.1	RO3G_00167.1	1179	702	86.47
##Scaffold_6	Scaffold_1	800185	810928	1	236038	251646	1	3		
800185	802365	236038	237457	1	1	RO3G_08294.1	RO3G_00092.1	323	335	55.22
805567	807665	241153	243327	-1	-1	RO3G_08297.1	RO3G_00095.1	729	453	78.59
809030	810928	249883	251646	1	1	RO3G_08298.1	RO3G_00099.1	783	504	78.17
##Scaffold_6	Scaffold_1	847739	916675	1	486036	563774	1	9		
847739	848993	486036	486775	-1	-1	RO3G_08318.1	RO3G_00194.1	389	211	93.36
850808	851873	494115	495180	1	1	RO3G_08319.1	RO3G_00198.1	652	337	99.41
859296	860906	509648	511095	-1	-1	RO3G_08323.1	RO3G_00204.1	818	417	92.09
863283	863649	517085	517463	-1	-1	RO3G_08326.1	RO3G_00209.1	135	69	98.55
871977	872666	525489	526277	1	1	RO3G_08331.1	RO3G_00212.1	351	264	64.77
881722	882999	533256	533978	-1	-1	RO3G_08336.1	RO3G_00214.1	303	242	67.36
903371	904395	543813	545009	1	1	RO3G_08344.1	RO3G_00216.1	207	342	45.32
914268	914816	559522	560457	1	1	RO3G_08347.1	RO3G_00223.1	274	182	71.98
916073	916675	563051	563774	-1	-1	RO3G_08348.1	RO3G_00226.1	182	187	63.1
##Scaffold_6	Scaffold_1	955647	981199	1	654546	678588	1	6		

955647	957410	654546	656393	1	1	RO3G_08359.1	RO3G_00266.1	649	579	67.7
959039	962179	658739	661879	1	1	RO3G_08360.1	RO3G_00267.1	1818	1006	92.84
964238	965584	666450	667721	-1	-1	RO3G_08362.1	RO3G_00269.1	585	449	74.83
965816	966444	668103	668718	-1	-1	RO3G_08363.1	RO3G_00270.1	142	110	100
968865	969749	669230	669983	-1	-1	RO3G_08365.1	RO3G_00271.1	187	203	52.71
974037	981199	672180	678588	1	1	RO3G_08367.1	RO3G_00272.1	2977	2012	77.34
##Scaffold_6	Scaffold_1	1071160	1087787	1	832002	848590	-1	6		
1086848	1087787	832002	832837	-1	1	RO3G_08413.1	RO3G_00334.1	253	267	61.8
1080867	1081686	837684	838528	-1	1	RO3G_08410.1	RO3G_00336.1	452	255	99.61
1077739	1079358	841490	842916	1	-1	RO3G_08408.1	RO3G_00338.1	427	399	56.39
1075501	1077035	843437	845082	-1	1	RO3G_08407.1	RO3G_00339.1	373	246	81.71
1073857	1075341	845284	846791	-1	1	RO3G_08406.1	RO3G_00340.1	800	456	87.28
1071160	1071521	848217	848590	1	-1	RO3G_08404.1	RO3G_00341.1	105	103	70.87
##Scaffold_6	Scaffold_1	1153773	1173157	1	785073	807634	1	4		
1153773	1157135	785073	789371	1	1	RO3G_08439.1	RO3G_00315.1	1686	929	92.9
1157426	1158028	789436	790032	-1	-1	RO3G_08440.1	RO3G_00316.1	266	171	87.13
1168075	1169404	804010	805187	1	1	RO3G_08444.1	RO3G_00319.1	313	226	64.6
1171385	1173157	805880	807634	1	1	RO3G_08446.1	RO3G_00320.1	486	524	56.87
##Scaffold_6	Scaffold_1	1097154	1113858	1	811849	822129	-1	3		
1113209	1113858	811849	813439	-1	1	RO3G_08424.1	RO3G_00324.1	166	135	57.78
1102415	1104643	816074	817733	1	-1	RO3G_08421.1	RO3G_00326.1	547	515	58.25
1097154	1098805	820465	822129	1	-1	RO3G_08419.1	RO3G_00329.1	735	422	91.94
##Scaffold_6	Scaffold_1	1198522	1217859	1	935161	969261	1	5		
1198522	1199378	935161	935913	-1	-1	RO3G_08459.1	RO3G_00379.1	255	201	68.16
1204967	1206097	942750	943529	1	1	RO3G_08461.1	RO3G_00383.1	385	242	72.31
1207551	1209769	955533	957527	-1	-1	RO3G_08462.1	RO3G_00389.1	496	434	59.91
1210170	1212004	958010	959884	-1	-1	RO3G_08463.1	RO3G_00390.1	594	513	66.86
1216779	1217859	968827	969261	-1	-1	RO3G_08466.1	RO3G_00393.1	185	94	95.74
##Scaffold_6	Scaffold_13	1916606	1922587	13	502896	509705	1	3		
1916606	1917925	502896	504383	1	1	RO3G_08757.1	RO3G_14049.1	412	449	53.67
1918295	1920542	504726	506688	-1	-1	RO3G_08758.1	RO3G_14050.1	608	594	56.9
1921163	1922587	508176	509705	-1	-1	RO3G_08759.1	RO3G_14051.1	649	409	82.89
##Scaffold_6	Scaffold_14	2383810	2408188	14	365178	380263	-1	3		
2406783	2408188	365178	366589	1	-1	RO3G_08940.1	RO3G_14478.1	795	435	95.86
2399857	2400995	374033	375162	1	-1	RO3G_08936.1	RO3G_14483.1	642	317	96.85
2383810	2384468	379649	380263	-1	1	RO3G_08929.1	RO3G_14486.1	191	194	56.19
##Scaffold_6	Scaffold_15	49667	75799	15	794944	824904	1	7		
49667	50470	794944	795784	1	1	RO3G_08000.1	RO3G_15091.1	258	166	95.78
50670	52247	796148	797733	-1	-1	RO3G_08001.1	RO3G_15092.1	542	396	77.53
58323	58986	807256	807904	-1	-1	RO3G_08003.1	RO3G_15095.1	300	146	98.63
59618	61789	810800	814260	-1	-1	RO3G_08004.1	RO3G_15097.1	369	486	45.88
66007	67073	818653	819508	-1	-1	RO3G_08006.1	RO3G_15098.1	124	135	47.41
67963	69054	820188	820857	-1	-1	RO3G_08007.1	RO3G_15100.1	235	168	70.24
73434	75799	822675	824904	1	1	RO3G_08009.1	RO3G_15101.1	748	690	61.16
##Scaffold_6	Scaffold_15	136087	145311	15	1063985	1076231	1	3		
136087	137209	1063985	1065205	1	1	RO3G_08033.1	RO3G_15197.1	538	327	84.4
139777	142178	1065520	1066497	-1	-1	RO3G_08035.1	RO3G_15198.1	336	235	78.3
144350	145311	1074433	1076231	-1	-1	RO3G_08037.1	RO3G_15202.1	412	249	85.14
##Scaffold_6	Scaffold_20	691599	726870	20	105145	146536	-1	7		
725182	726870	105145	106833	1	-1	RO3G_08262.1	RO3G_16396.1	949	501	99.6
721416	722149	107283	108021	-1	1	RO3G_08261.1	RO3G_16397.1	292	163	93.25
715288	715727	113910	114352	1	-1	RO3G_08258.1	RO3G_16401.1	119	108	96.3
712898	714374	117450	118175	1	-1	RO3G_08257.1	RO3G_16403.1	393	198	95.96
706380	707642	133426	134692	1	-1	RO3G_08255.1	RO3G_16410.1	703	383	89.3
700869	701742	135696	136510	1	-1	RO3G_08253.1	RO3G_16411.1	281	263	69.58
691599	693184	145063	146536	-1	1	RO3G_08248.1	RO3G_16415.1	545	426	68.08
##Scaffold_6	Scaffold_20	634044	685109	20	198253	215758	-1	6		
684246	685109	198253	199127	-1	1	RO3G_08245.1	RO3G_16436.1	262	195	76.92
676705	677720	202056	203600	-1	1	RO3G_08242.1	RO3G_16438.1	409	245	80.82
675360	676376	204120	206009	1	-1	RO3G_08241.1	RO3G_16439.1	213	154	85.71
642879	643879	208447	209587	-1	1	RO3G_08225.1	RO3G_16440.1	389	241	79.25
634994	635305	214452	214763	1	-1	RO3G_08219.1	RO3G_16444.1	162	82	100
634044	634454	215348	215758	-1	1	RO3G_08218.1	RO3G_16445.1	262	136	100
##Scaffold_6	Scaffold_24	2619607	2670621	24	178834	222655	-1	9		
2667679	2670621	178834	181803	1	-1	RO3G_09029.1	RO3G_16993.1	695	629	65.98
2664468	2667379	182117	185302	1	-1	RO3G_09028.1	RO3G_16994.1	722	450	80.67
2658432	2660888	186161	188668	1	-1	RO3G_09026.1	RO3G_16995.1	1105	702	75.07
2656466	2656765	190476	190780	-1	1	RO3G_09025.1	RO3G_16996.1	150	82	90.24
2651250	2652179	192743	193672	-1	1	RO3G_09023.1	RO3G_16997.1	476	309	99.03

2649300	2650923	194278	196446	1	-1	RO3G_09022.1	RO3G_16998.1	471	372	65.59
2641179	2646492	197028	202786	1	-1	RO3G_09020.1	RO3G_16999.1	887	1510	41.99
2626427	2627000	213247	214194	-1	1	RO3G_09016.1	RO3G_17002.1	174	138	57.25
2619607	2620427	221828	222655	-1	1	RO3G_09012.1	RO3G_17006.1	451	224	100
##Scaffold_6	Scaffold_9	247663	272049	9	2196634	2211154	1	4		
247663	248670	2196634	2197692	-1	-1	RO3G_08083.1	RO3G_11915.1	392	346	73.7
257653	258457	2202388	2202852	-1	-1	RO3G_08086.1	RO3G_11918.1	115	109	48.62
265599	267128	2207027	2209342	1	1	RO3G_08089.1	RO3G_11920.1	535	348	72.99
270284	272049	2209539	2211154	-1	-1	RO3G_08092.1	RO3G_11921.1	630	418	78.95
##Scaffold_6	Scaffold_9	1314169	1355311	9	1882142	1915557	1	9		
1314169	1315047	1882142	1883447	-1	-1	RO3G_08506.1	RO3G_11792.1	268	190	67.37
1318701	1319636	1883982	1884886	1	1	RO3G_08508.1	RO3G_11793.1	126	171	46.2
1320005	1321434	1889291	1889969	-1	-1	RO3G_08509.1	RO3G_11795.1	193	139	74.1
1325177	1326111	1900102	1901037	1	1	RO3G_08513.1	RO3G_11799.1	442	274	100
1327643	1328242	1903469	1904016	1	1	RO3G_08515.1	RO3G_11801.1	182	103	88.35
1332049	1333081	1904969	1905874	1	1	RO3G_08517.1	RO3G_11802.1	522	260	98.85
1349167	1351583	1909499	1911669	-1	-1	RO3G_08525.1	RO3G_11804.1	579	388	78.87
1352064	1353258	1912809	1914068	-1	-1	RO3G_08526.1	RO3G_11805.1	688	358	92.18
1354061	1355311	1914650	1915557	-1	-1	RO3G_08527.1	RO3G_11806.1	259	157	80.89
##Scaffold_6	Scaffold_9	1445802	1502760	9	1951535	1982241	1	5		
1445802	1446401	1951535	1952176	1	1	RO3G_08564.1	RO3G_11819.1	220	213	60.56
1447309	1448271	1957828	1959492	-1	-1	RO3G_08565.1	RO3G_11822.1	387	367	59.95
1449895	1450815	1960504	1961491	-1	-1	RO3G_08566.1	RO3G_11823.1	229	283	58.66
1468461	1470351	1967429	1969116	1	1	RO3G_08575.1	RO3G_11826.1	595	450	67.78
1499908	1502760	1979475	1982241	1	1	RO3G_08587.1	RO3G_11831.1	852	823	62.45
##Scaffold_6	Scaffold_9	1390817	1421557	9	1995584	2007251	-1	6		
1420628	1421557	1995584	1996512	1	-1	RO3G_08555.1	RO3G_11836.1	341	234	82.48
1411759	1412190	1999153	1999588	1	-1	RO3G_08551.1	RO3G_11838.1	134	83	84.34
1401921	1402850	2000727	2002522	1	-1	RO3G_08547.1	RO3G_11839.1	100	131	42.75
1395891	1396934	2003060	2004089	1	-1	RO3G_08543.1	RO3G_11840.1	425	269	89.96
1393678	1394387	2005182	2005977	-1	1	RO3G_08541.1	RO3G_11841.1	185	137	61.31
1390817	1391851	2006522	2007251	1	-1	RO3G_08540.1	RO3G_11842.1	101	154	57.79
##Scaffold_6	Scaffold_9	1475025	1494594	9	2066991	2085975	-1	3		
1492129	1494594	2066991	2069446	-1	1	RO3G_08585.1	RO3G_11870.1	1286	787	85.39
1485431	1489226	2071898	2075695	1	-1	RO3G_08583.1	RO3G_11872.1	1624	1235	65.18
1475025	1475585	2085415	2085975	1	-1	RO3G_08578.1	RO3G_11878.1	363	186	100
##Scaffold_6	Scaffold_9	1760063	1771191	9	1763038	1768992	-1	3		
1769935	1771191	1763038	1764265	1	-1	RO3G_08703.1	RO3G_11748.1	527	337	88.13
1766419	1769488	1764690	1767481	1	-1	RO3G_08702.1	RO3G_11749.1	722	740	55.27
1760063	1760308	1768747	1768992	-1	1	RO3G_08699.1	RO3G_11750.1	134	80	76.25
##Scaffold_7	Scaffold_1	616962	648491	1	2357046	2395646	1	7		
616962	618451	2357046	2358601	-1	-1	RO3G_09434.1	RO3G_00928.1	313	335	51.04
621022	622099	2362777	2363787	1	1	RO3G_09435.1	RO3G_00930.1	146	176	50.57
625083	625598	2377959	2378477	-1	-1	RO3G_09437.1	RO3G_00935.1	182	172	66.86
632491	633266	2381629	2382382	-1	-1	RO3G_09441.1	RO3G_00937.1	329	194	84.54
638102	638791	2383673	2384366	1	1	RO3G_09443.1	RO3G_00938.1	227	129	100
642076	643556	2386729	2388117	1	1	RO3G_09445.1	RO3G_00940.1	225	159	78.62
647547	648491	2394231	2395646	1	1	RO3G_09448.1	RO3G_00944.1	127	301	33.55
##Scaffold_7	Scaffold_1	693558	704247	1	1988610	2001432	1	4		
693558	694330	1988610	1989401	-1	-1	RO3G_09466.1	RO3G_00782.1	262	139	97.12
696908	697807	1990975	1992436	1	1	RO3G_09467.1	RO3G_00784.1	348	241	71.78
700677	702201	1996455	1998262	-1	-1	RO3G_09469.1	RO3G_00787.1	806	459	89.11
702821	704247	1999999	2001432	-1	-1	RO3G_09470.1	RO3G_00788.1	908	458	94.32
##Scaffold_7	Scaffold_1	709382	719654	1	2829063	2837823	1	3		
709382	710410	2829063	2830103	1	1	RO3G_09474.1	RO3G_01119.1	427	357	68.63
711026	712238	2830284	2831463	1	1	RO3G_09475.1	RO3G_01120.1	506	355	72.39
717503	719654	2835635	2837823	1	1	RO3G_09477.1	RO3G_01122.1	939	546	87
##Scaffold_7	Scaffold_10	1612814	1632916	10	1414910	1426497	-1	4		
1632073	1632916	1414910	1415732	-1	1	RO3G_09817.1	RO3G_12499.1	377	190	95.26
1626622	1627394	1419154	1419905	1	-1	RO3G_09814.1	RO3G_12500.1	200	190	53.68
1614670	1615725	1423894	1424946	-1	1	RO3G_09808.1	RO3G_12503.1	130	325	34.15
1612814	1613998	1425290	1426497	-1	1	RO3G_09807.1	RO3G_12504.1	326	262	64.5
##Scaffold_7	Scaffold_11	968739	983700	11	455270	467845	1	4		
968739	971172	455270	457630	-1	-1	RO3G_09565.1	RO3G_12847.1	597	716	54.47
975546	976061	460827	461661	1	1	RO3G_09567.1	RO3G_12849.1	282	146	93.84
979475	980824	463814	465216	1	1	RO3G_09569.1	RO3G_12852.1	464	323	76.47
982544	983700	466206	467845	-1	-1	RO3G_09570.1	RO3G_12853.1	274	211	70.14

##Scaffold_7	Scaffold_11	2012829	2040537	11	793284	825051	1	5		
2012829	2014011	793284	794731	-1	-1	RO3G_09971.1	RO3G_12983.1	332	251	69.32
2017609	2018681	798438	800350	-1	-1	RO3G_09974.1	RO3G_12984.1	347	299	66.56
2021520	2022797	810030	811428	1	1	RO3G_09976.1	RO3G_12988.1	351	344	51.74
2024581	2025308	812979	813735	1	1	RO3G_09978.1	RO3G_12989.1	329	233	81.55
2039276	2040537	824237	825051	1	1	RO3G_09984.1	RO3G_12994.1	330	196	80.1
##Scaffold_7	Scaffold_11	2118455	2140017	11	1051541	1077070	1	7		
2118455	2119687	1051541	1053088	-1	-1	RO3G_10020.1	RO3G_13078.1	287	242	64.46
2122455	2124043	1055117	1055825	1	1	RO3G_10022.1	RO3G_13080.1	224	165	70.3
2124465	2125731	1057973	1059248	1	1	RO3G_10023.1	RO3G_13082.1	559	333	97.3
2126301	2126825	1059818	1060344	1	1	RO3G_10024.1	RO3G_13083.1	221	139	98.56
2135249	2135774	1067739	1068272	1	1	RO3G_10028.1	RO3G_13087.1	300	155	92.9
2136755	2137687	1070052	1070958	-1	-1	RO3G_10030.1	RO3G_13088.1	186	287	42.86
2138601	2140017	1074801	1077070	-1	-1	RO3G_10031.1	RO3G_13089.1	470	335	71.34
##Scaffold_7	Scaffold_11	2210606	2234153	11	1099966	1144592	1	5		
2210606	2211915	1099966	1101185	-1	-1	RO3G_10055.1	RO3G_13097.1	568	336	92.86
2213341	2216911	1109666	1113621	-1	-1	RO3G_10056.1	RO3G_13100.1	1696	1072	76.87
2223968	2225736	1129970	1131967	1	1	RO3G_10059.1	RO3G_13109.1	730	561	72.37
2229823	2232101	1137472	1139778	1	1	RO3G_10063.1	RO3G_13112.1	1252	687	91.7
2233074	2234153	1143698	1144592	1	1	RO3G_10064.1	RO3G_13115.1	124	173	59.54
##Scaffold_7	Scaffold_11	2448038	2475124	11	920889	965346	-1	5		
2471185	2475124	920889	923845	1	-1	RO3G_10152.1	RO3G_13032.1	976	834	63.07
2464669	2470275	926036	930545	-1	1	RO3G_10151.1	RO3G_13033.1	2404	1422	84.46
2459195	2460075	945093	946170	-1	1	RO3G_10149.1	RO3G_13037.1	125	95	68.42
2454580	2456418	954473	956342	1	-1	RO3G_10147.1	RO3G_13040.1	882	515	82.52
2448038	2450884	962538	965346	-1	1	RO3G_10145.1	RO3G_13043.1	933	754	65.52
##Scaffold_7	Scaffold_16	1816719	1822141	16	73424	81438	-1	3		
1821281	1822141	73424	74818	-1	1	RO3G_09900.1	RO3G_15299.1	172	184	50
1818096	1818892	79058	79832	1	-1	RO3G_09898.1	RO3G_15303.1	327	181	96.13
1816719	1817559	80070	81438	-1	1	RO3G_09897.1	RO3G_15304.1	140	147	48.98
##Scaffold_7	Scaffold_16	2294371	2335446	16	172916	195670	-1	4		
2333558	2335446	172916	174785	-1	1	RO3G_10106.1	RO3G_15341.1	912	608	76.97
2330152	2331229	177558	178611	1	-1	RO3G_10104.1	RO3G_15344.1	358	283	63.96
2301045	2301635	190722	191304	-1	1	RO3G_10092.1	RO3G_15350.1	252	156	99.36
2294371	2295525	194535	195670	1	-1	RO3G_10088.1	RO3G_15353.1	566	357	84.87
##Scaffold_7	Scaffold_16	2592608	2606585	16	441913	448830	-1	4		
2605138	2606585	441913	443381	-1	1	RO3G_10204.1	RO3G_15450.1	697	442	80.77
2603304	2604495	443523	445351	1	-1	RO3G_10202.1	RO3G_15451.1	312	371	65.77
2594894	2595745	445524	446394	-1	1	RO3G_10200.1	RO3G_15452.1	400	220	93.18
2592608	2593733	446699	448830	-1	1	RO3G_10199.1	RO3G_15453.1	451	372	75.54
##Scaffold_7	Scaffold_17	1176114	1190977	17	557639	581860	-1	6		
1190228	1190977	557639	558541	-1	1	RO3G_09651.1	RO3G_15790.1	390	250	90.4
1187256	1190005	558764	561513	-1	1	RO3G_09650.1	RO3G_15791.1	1667	843	100
1185097	1187100	562193	563638	1	-1	RO3G_09649.1	RO3G_15792.1	343	294	65.65
1181276	1181920	571382	573097	-1	1	RO3G_09646.1	RO3G_15796.1	128	149	55.03
1179905	1180999	573253	574377	-1	1	RO3G_09645.1	RO3G_15797.1	402	241	100
1176114	1177740	580229	581860	-1	1	RO3G_09643.1	RO3G_15800.1	750	543	81.4
##Scaffold_7	Scaffold_2	1332331	1365677	2	4260288	4281878	1	3		
1332331	1334962	4260288	4265748	-1	-1	RO3G_09709.1	RO3G_03905.1	1227	833	73.47
1340520	1341984	4267813	4269291	-1	-1	RO3G_09711.1	RO3G_03906.1	663	370	91.89
1364389	1365677	4280954	4281878	1	1	RO3G_09721.1	RO3G_03909.1	253	341	63.05
##Scaffold_7	Scaffold_2	1579272	1589905	2	4745194	4755888	1	4		
1579272	1582504	4745194	4748425	-1	-1	RO3G_09793.1	RO3G_04076.1	1281	836	80.5
1582997	1583916	4750392	4751310	1	1	RO3G_09794.1	RO3G_04077.1	485	268	86.94
1584589	1586505	4753157	4753915	1	1	RO3G_09795.1	RO3G_04078.1	404	247	80.16
1588627	1589905	4754622	4755888	-1	-1	RO3G_09797.1	RO3G_04079.1	757	388	91.49
##Scaffold_7	Scaffold_3	282766	306127	3	286376	305024	-1	5		
303766	306127	286376	288723	1	-1	RO3G_09309.1	RO3G_04323.1	1295	723	92.53
300702	303036	288974	291845	1	-1	RO3G_09308.1	RO3G_04324.1	821	678	78.91
297152	297511	296040	296389	-1	1	RO3G_09306.1	RO3G_04326.1	141	101	91.09
292377	292950	300900	301470	1	-1	RO3G_09303.1	RO3G_04330.1	125	156	65.38
282766	285909	302197	305024	-1	1	RO3G_09301.1	RO3G_04331.1	847	990	53.03
##Scaffold_7	Scaffold_4	2087308	2113149	4	2237170	2259379	1	5		
2087308	2088384	2237170	2238048	-1	-1	RO3G_10005.1	RO3G_06324.1	437	258	80.62
2092357	2093959	2248149	2250337	-1	-1	RO3G_10007.1	RO3G_06329.1	542	382	78.8
2098919	2099682	2251485	2252431	-1	-1	RO3G_10009.1	RO3G_06330.1	239	167	73.65
2106809	2107377	2255946	2256559	-1	-1	RO3G_10014.1	RO3G_06332.1	214	106	100
2112741	2113149	2258902	2259379	1	1	RO3G_10017.1	RO3G_06334.1	150	138	68.84

##Scaffold_7	Scaffold_4	2238609	2265050	4	2206181	2226869	-1	5		
2264274	2265050	2206181	2206836	-1	1	RO3G_10076.1	RO3G_06314.1	197	126	79.37
2255156	2257614	2210621	2213295	-1	1	RO3G_10073.1	RO3G_06317.1	576	700	51.43
2253998	2254792	2213649	2214453	1	-1	RO3G_10072.1	RO3G_06318.1	320	176	83.52
2243731	2245458	2222477	2225136	-1	1	RO3G_10068.1	RO3G_06320.1	423	459	55.77
2238609	2239784	2225710	2226869	-1	1	RO3G_10066.1	RO3G_06321.1	520	326	84.05
##Scaffold_8	Scaffold_1	1292334	1312917	1	4065932	4085664	1	4		
1292334	1293342	4065932	4066982	-1	-1	RO3G_10690.1	RO3G_01567.1	315	321	52.96
1293689	1295221	4069170	4070147	-1	-1	RO3G_10691.1	RO3G_01568.1	173	239	47.7
1308108	1310699	4080081	4082695	1	1	RO3G_10698.1	RO3G_01572.1	1269	729	89.85
1312075	1312917	4084840	4085664	-1	-1	RO3G_10700.1	RO3G_01575.1	375	233	78.97
##Scaffold_8	Scaffold_11	403706	485261	11	532841	596893	-1	8		
483336	485261	532841	534853	1	-1	RO3G_10408.1	RO3G_12883.1	1023	536	95.9
474745	477184	535833	538644	1	-1	RO3G_10406.1	RO3G_12884.1	886	661	77.31
458934	460909	547449	549439	1	-1	RO3G_10403.1	RO3G_12888.1	1092	610	97.21
451193	457439	553556	559848	1	-1	RO3G_10401.1	RO3G_12891.1	2891	1959	78.76
431233	433002	568351	570212	1	-1	RO3G_10395.1	RO3G_12896.1	786	526	72.05
419537	421758	575026	577107	-1	1	RO3G_10391.1	RO3G_12898.1	801	619	66.07
418629	419373	577171	577924	1	-1	RO3G_10390.1	RO3G_12899.1	187	152	63.82
403706	404966	595611	596893	-1	1	RO3G_10385.1	RO3G_12908.1	522	314	80.57
##Scaffold_8	Scaffold_11	387225	397690	11	691772	697662	1	3		
387225	387815	691772	692352	1	1	RO3G_10377.1	RO3G_12944.1	323	159	98.11
395750	396437	695570	696612	1	1	RO3G_10382.1	RO3G_12946.1	273	187	71.12
396790	397690	696945	697662	-1	-1	RO3G_10383.1	RO3G_12947.1	375	219	86.3
##Scaffold_8	Scaffold_11	556378	581483	11	83661	120048	-1	5		
580470	581483	83661	86114	-1	1	RO3G_10445.1	RO3G_12696.1	278	326	50.31
574702	577051	95501	97828	-1	1	RO3G_10443.1	RO3G_12701.1	1173	659	86.34
567051	568798	99754	101281	1	-1	RO3G_10440.1	RO3G_12703.1	710	539	72.91
564463	565240	104534	105426	1	-1	RO3G_10439.1	RO3G_12705.1	237	202	60.4
556378	557527	118901	120048	1	-1	RO3G_10435.1	RO3G_12710.1	519	294	85.71
##Scaffold_8	Scaffold_18	316233	338714	18	274725	301352	-1	3		
338202	338714	274725	275378	1	-1	RO3G_10362.1	RO3G_15979.1	221	172	70.35
319361	322163	290919	292995	1	-1	RO3G_10355.1	RO3G_15987.1	856	535	82.24
316233	317748	299948	301352	1	-1	RO3G_10354.1	RO3G_15990.1	546	449	67.26
##Scaffold_8	Scaffold_2	705107	712714	2	4540444	4551445	1	3		
705107	705734	4540444	4541082	-1	-1	RO3G_10491.1	RO3G_04000.1	365	191	99.48
709779	710185	4547685	4548535	1	1	RO3G_10493.1	RO3G_04002.1	128	121	59.5
711103	712714	4548933	4551445	1	1	RO3G_10494.1	RO3G_04003.1	189	170	57.65
##Scaffold_8	Scaffold_2	985593	990263	2	2129349	2135611	1	3		
985593	986662	2129349	2130444	1	1	RO3G_10591.1	RO3G_03043.1	430	281	71.89
987413	987919	2130574	2131077	1	1	RO3G_10592.1	RO3G_03044.1	219	168	67.26
989674	990263	2134936	2135611	-1	-1	RO3G_10593.1	RO3G_03047.1	166	195	53.33
##Scaffold_8	Scaffold_2	1100145	1112456	2	2050725	2066473	1	3		
1100145	1101283	2050725	2051884	1	1	RO3G_10625.1	RO3G_03011.1	380	347	71.76
1105497	1108957	2056112	2058511	-1	-1	RO3G_10627.1	RO3G_03013.1	659	867	50.75
1111005	1112456	2065297	2066473	1	1	RO3G_10629.1	RO3G_03017.1	137	323	33.13
##Scaffold_8	Scaffold_2	1083214	1088365	2	2104655	2110144	1	3		
1083214	1084378	2104655	2107445	1	1	RO3G_10618.1	RO3G_03032.1	384	406	59.11
1085112	1085893	2107483	2108428	-1	-1	RO3G_10619.1	RO3G_03033.1	315	190	85.26
1086016	1088365	2108740	2110144	-1	-1	RO3G_10620.1	RO3G_03034.1	653	403	79.9
##Scaffold_8	Scaffold_2	1144297	1150210	2	2486017	2492601	-1	3		
1149051	1150210	2486017	2487542	-1	1	RO3G_10647.1	RO3G_03191.1	578	314	93.63
1147362	1148644	2487753	2489033	1	-1	RO3G_10646.1	RO3G_03192.1	493	409	80.93
1144297	1146813	2489909	2492601	-1	1	RO3G_10645.1	RO3G_03193.1	1049	819	70.21
##Scaffold_8	Scaffold_3	2053797	2075970	3	2311510	2328729	-1	5		
2074644	2075970	2311510	2312828	1	-1	RO3G_10978.1	RO3G_05099.1	383	269	75.46
2072849	2074143	2313367	2314656	-1	1	RO3G_10977.1	RO3G_05100.1	513	330	79.39
2066247	2068511	2315079	2317361	-1	1	RO3G_10974.1	RO3G_05101.1	1061	703	73.83
2060028	2065387	2318215	2320106	1	-1	RO3G_10973.1	RO3G_05103.1	493	335	88.06
2053797	2058252	2324277	2328729	1	-1	RO3G_10971.1	RO3G_05106.1	2786	1461	93.5
##Scaffold_8	Scaffold_3	1973128	1997672	3	2452849	2490644	-1	5		
1997333	1997672	2452849	2453207	-1	1	RO3G_10949.1	RO3G_05152.1	150	75	98.67
1987581	1988653	2462479	2463537	1	-1	RO3G_10943.1	RO3G_05156.1	476	245	95.1
1986310	1987236	2474494	2475747	1	-1	RO3G_10942.1	RO3G_05159.1	327	317	59.94
1979675	1980622	2487332	2488189	1	-1	RO3G_10940.1	RO3G_05165.1	139	143	60.84
1973128	1975011	2488491	2490644	1	-1	RO3G_10937.1	RO3G_05166.1	955	594	75.08

##Scaffold_8	Scaffold_3	2115450	2136388	3	2280030	2301426	-1	7		
2135288	2136388	2280030	2281131	1	-1	RO3G_11004.1	RO3G_05085.1	601	310	96.77
2132669	2133769	2281486	2282583	1	-1	RO3G_11003.1	RO3G_05086.1	566	330	91.52
2127129	2128647	2283244	2284621	-1	1	RO3G_11000.1	RO3G_05087.1	566	345	81.74
2124201	2124653	2287057	2287896	1	-1	RO3G_10998.1	RO3G_05088.1	265	150	98
2122676	2123600	2288139	2289094	-1	1	RO3G_10997.1	RO3G_05089.1	445	220	97.27
2118018	2118708	2297561	2298135	-1	1	RO3G_10994.1	RO3G_05092.1	172	126	61.9
2115450	2116329	2300445	2301426	-1	1	RO3G_10992.1	RO3G_05094.1	249	287	61.32
##Scaffold_8	Scaffold_3	2206482	2225014	3	1943152	1958910	1	4		
2206482	2207186	1943152	1943856	-1	-1	RO3G_11031.1	RO3G_04941.1	328	234	82.91
2213607	2215084	1951832	1953437	-1	-1	RO3G_11034.1	RO3G_04947.1	521	495	61.01
2223782	2224338	1957186	1957739	-1	-1	RO3G_11037.1	RO3G_04949.1	301	148	97.3
2224654	2225014	1957907	1958910	1	1	RO3G_11038.1	RO3G_04950.1	152	68	97.06
##Scaffold_8	Scaffold_3	2268351	2279958	3	2407668	2424933	-1	3		
2279269	2279958	2407668	2408866	-1	1	RO3G_11061.1	RO3G_05137.1	192	145	73.79
2274238	2274798	2413581	2414111	-1	1	RO3G_11057.1	RO3G_05140.1	231	142	88.73
2268351	2271815	2416043	2424933	-1	1	RO3G_11055.1	RO3G_05142.1	772	516	78.49
##Scaffold_9	Scaffold_1	725339	826321	1	3888803	3897497	-1	3		
825673	826321	3888803	3889450	1	-1	RO3G_11396.1	RO3G_01495.1	323	171	97.66
732951	733759	3892367	3893677	-1	1	RO3G_11359.1	RO3G_01499.1	190	215	56.28
725339	726915	3896219	3897497	-1	1	RO3G_11355.1	RO3G_01501.1	715	373	97.32
##Scaffold_9	Scaffold_12	334531	341466	12	917035	926325	-1	3		
339394	341466	917035	919225	-1	1	RO3G_11231.1	RO3G_13634.1	753	594	64.31
335729	337927	921387	923587	1	-1	RO3G_11230.1	RO3G_13636.1	1148	673	99.85
334531	335178	925665	926325	1	-1	RO3G_11229.1	RO3G_13638.1	232	120	100
##Scaffold_9	Scaffold_2	921449	960644	2	1450893	1474522	1	5		
947923	949409	1450893	1452411	1	1	RO3G_11439.1	RO3G_02786.1	704	472	73.52
950998	952018	1456930	1458009	1	1	RO3G_11441.1	RO3G_02789.1	269	206	69.9
953644	954771	1467356	1468496	-1	-1	RO3G_11443.1	RO3G_02794.1	590	340	84.12
959092	960644	1469358	1471461	-1	-1	RO3G_11445.1	RO3G_02795.1	723	398	94.22
921449	923225	1473186	1474522	-1	-1	RO3G_11430.1	RO3G_02797.1	278	378	42.59
##Scaffold_9	Scaffold_2	1068482	1105756	2	1312519	1349373	1	7		
1068482	1070398	1312519	1314406	1	1	RO3G_11488.1	RO3G_02730.1	796	497	78.07
1070869	1074757	1314824	1318560	-1	-1	RO3G_11489.1	RO3G_02731.1	1204	1057	68.78
1079139	1080890	1320574	1322356	1	1	RO3G_11492.1	RO3G_02733.1	401	513	43.27
1089509	1090698	1323684	1326296	1	1	RO3G_11494.1	RO3G_02735.1	566	298	99.66
1092360	1094721	1327681	1330056	-1	-1	RO3G_11496.1	RO3G_02737.1	1203	771	83.53
1096771	1097133	1339712	1340081	1	1	RO3G_11497.1	RO3G_02740.1	200	99	96.97
1105154	1105756	1348339	1349373	-1	-1	RO3G_11500.1	RO3G_02743.1	234	194	55.67
##Scaffold_9	Scaffold_2	1343750	1352465	2	1796110	1805114	1	3		
1343750	1344831	1796110	1797180	1	1	RO3G_11596.1	RO3G_02914.1	562	322	86.96
1345680	1348345	1798502	1801178	-1	-1	RO3G_11597.1	RO3G_02915.1	1009	748	73.53
1350959	1352465	1803770	1805114	-1	-1	RO3G_11598.1	RO3G_02916.1	97.1	102	48.04
##Scaffold_9	Scaffold_2	1492234	1504471	2	1503940	1515521	1	4		
1492234	1493229	1503940	1505499	-1	-1	RO3G_11650.1	RO3G_02809.1	345	174	97.7
1493379	1497629	1505807	1510416	-1	-1	RO3G_11651.1	RO3G_02810.1	1299	1168	65.41
1498598	1500973	1511284	1513230	-1	-1	RO3G_11652.1	RO3G_02811.1	817	658	73.56
1503407	1504471	1514433	1515521	1	1	RO3G_11653.1	RO3G_02812.1	446	292	73.97
##Scaffold_9	Scaffold_2	1553436	1580121	2	1044713	1072209	1	3		
1553436	1555542	1044713	1046360	-1	-1	RO3G_11676.1	RO3G_02628.1	489	433	59.12
1561774	1564047	1052387	1053606	-1	-1	RO3G_11678.1	RO3G_02631.1	346	262	65.65
1578675	1580121	1070753	1072209	-1	-1	RO3G_11684.1	RO3G_02639.1	630	378	81.22
##Scaffold_9	Scaffold_2	1629355	1650133	2	1091132	1104633	1	3		
1629355	1633760	1091132	1094874	1	1	RO3G_11701.1	RO3G_02645.1	845	637	69.86
1642598	1643959	1100227	1101585	1	1	RO3G_11703.1	RO3G_02649.1	780	412	96.84
1647678	1650133	1102144	1104633	1	1	RO3G_11706.1	RO3G_02650.1	928	668	68.41
##Scaffold_9	Scaffold_2	1691613	1713832	2	1733597	1767308	1	5		
1691613	1694302	1733597	1737215	-1	-1	RO3G_11725.1	RO3G_02895.1	1144	823	74
1697511	1699271	1747510	1749054	-1	-1	RO3G_11728.1	RO3G_02898.1	520	360	76.39
1701007	1701804	1754198	1755912	-1	-1	RO3G_11729.1	RO3G_02899.1	283	248	63.71
1705933	1707249	1759268	1760651	-1	-1	RO3G_11731.1	RO3G_02901.1	342	275	58.91
1710465	1713832	1765914	1767308	1	1	RO3G_11732.1	RO3G_02903.1	633	496	70.56
##Scaffold_9	Scaffold_3	126263	147370	3	625467	641702	-1	4		
145804	147370	625467	627019	-1	1	RO3G_11162.1	RO3G_04451.1	832	429	99.53
135927	138289	629178	631452	1	-1	RO3G_11158.1	RO3G_04453.1	820	644	64.29

128981	129689	638340	639056	1	-1	RO3G_11156.1	RO3G_04456.1	360	180	98.89		
126263	127162	640566	641702	-1	1	RO3G_11155.1	RO3G_04458.1	319	302	60.93		
##Scaffold_9	Scaffold_6	1759289	1767481	6	1766419	1777959	-1	3				
1764690	1767481	1766419	1769488	-1	1	RO3G_11749.1	RO3G_08702.1	722	740	55.27		
1763038	1764265	1769935	1771191	-1	1	RO3G_11748.1	RO3G_08703.1	527	337	88.13		
1759289	1761269	1775944	1777959	-1	1	RO3G_11747.1	RO3G_08705.1	919	619	89.82		
##Scaffold_9	Scaffold_6	1882142	1915557	6	1314169	1355311	1	9				
1882142	1883447	1314169	1315047	-1	-1	RO3G_11792.1	RO3G_08506.1	268	190	67.37		
1883982	1884886	1318701	1319636	1	1	RO3G_11793.1	RO3G_08508.1	126	171	46.2		
1889291	1889969	1320005	1321434	-1	-1	RO3G_11795.1	RO3G_08509.1	193	139	74.1		
1900102	1901037	1325177	1326111	1	1	RO3G_11799.1	RO3G_08513.1	442	274	100		
1903469	1904016	1327643	1328242	1	1	RO3G_11801.1	RO3G_08515.1	182	103	88.35		
1904969	1905874	1332049	1333081	1	1	RO3G_11802.1	RO3G_08517.1	522	260	98.85		
1909499	1911669	1349167	1351583	-1	-1	RO3G_11804.1	RO3G_08525.1	579	388	78.87		
1912809	1914068	1352064	1353258	-1	-1	RO3G_11805.1	RO3G_08526.1	688	358	92.18		
1914650	1915557	1354061	1355311	-1	-1	RO3G_11806.1	RO3G_08527.1	259	157	80.89		
##Scaffold_9	Scaffold_6	1957828	1969116	6	1447309	1470351	1	3				
1957828	1959492	1447309	1448271	-1	-1	RO3G_11822.1	RO3G_08565.1	387	367	59.95		
1960504	1961491	1449895	1450815	-1	-1	RO3G_11823.1	RO3G_08566.1	229	283	58.66		
1967429	1969116	1468461	1470351	1	1	RO3G_11826.1	RO3G_08575.1	595	450	67.78		
##Scaffold_9	Scaffold_6	1992704	2005977	6	1393678	1426579	-1	6				
2005182	2005977	1393678	1394387	1	-1	RO3G_11841.1	RO3G_08541.1	185	137	61.31		
2003060	2004089	1395891	1396934	-1	1	RO3G_11840.1	RO3G_08543.1	425	269	89.96		
2000727	2002522	1401921	1402850	-1	1	RO3G_11839.1	RO3G_08547.1	100	131	42.75		
1999153	1999588	1411759	1412190	-1	1	RO3G_11838.1	RO3G_08551.1	134	83	84.34		
1995584	1996512	1420628	1421557	-1	1	RO3G_11836.1	RO3G_08555.1	341	234	82.48		
1992704	1994438	1424892	1426579	-1	1	RO3G_11835.1	RO3G_08556.1	734	394	97.46		
##Scaffold_9	Scaffold_6	2196634	2211154	6	247663	272049	1	4				
2196634	2197692	247663	248670	-1	-1	RO3G_11915.1	RO3G_08083.1	392	346	73.7		
2202388	2202852	257653	258457	-1	-1	RO3G_11918.1	RO3G_08086.1	115	109	48.62		
2207027	2209342	265599	267128	1	1	RO3G_11920.1	RO3G_08089.1	535	348	72.99		
2209539	2211154	270284	272049	-1	-1	RO3G_11921.1	RO3G_08092.1	630	418	78.95		
##Scaffold_9	Scaffold_6	2066038	2075695	6	1485431	1496527	-1	3				
2071898	2075695	1485431	1489226	-1	1	RO3G_11872.1	RO3G_08583.1	1624	1235	65.18		
2066991	2069446	1492129	1494594	1	-1	RO3G_11870.1	RO3G_08585.1	1286	787	85.39		
2066038	2066641	1495375	1496527	1	-1	RO3G_11869.1	RO3G_08586.1	332	182	81.32		

* the lines start with ## define each syntenic block, display the corresponding scaffolds in the genome, coordinates where each syntenic regions start and stop, orientation and number of genes that define each region. The lines within each block are detailed information of each gene pair including start/stop of first gene, start/stop of second genes orientation of first genes orientation of second genes, first genes, second gene, annotated gene length of first gene, annotation length of second gene and sequence identity of the pair.
