# Supplementary material for: Genomic Analysis of the Basal Lineage Fungus Rhizopus oryzae Reveals a Whole-Genome Duplication
Source: PLoS Genet. 2009 Jul 3;5(7):e1000549. doi: 10.1371/journal.pgen.1000549 (PMC2699053; doi:10.1371/journal.pgen.1000549)
Supplement: Table S7 — Best-blast hits between P. blakesleeanus and R. oryzae. (0.07 MB PDF) [file pgen.1000549.s014.pdf]

Table S7 Best-blast hits between *P. blakesleeanus* and *R. oryzae*

	P. blakesleeanus blast against *R. oryzae*	*R. oryzae* blast against *P. blakesleeanus*	*R. oryzae* duplicates blast against *P. blakesleeanus*
Total genes with blast hits[a]	6687	8048	1015
1-to-1 hits [b]	3579	3533	129
2-to-1 hits [c]	1692	2726	852

[a] The best hits are based on single linkage blast from query to the reference protein set (1e-20, 60% length cutoff);
[b] 1-to-1 hits: only one homologous in the other genome;
[c] 2 genes hit the same gene in the other as the best blast hit.
