# Supplementary material for: Genomic Analysis of the Basal Lineage Fungus Rhizopus oryzae Reveals a Whole-Genome Duplication
Source: PLoS Genet. 2009 Jul 3;5(7):e1000549. doi: 10.1371/journal.pgen.1000549 (PMC2699053; doi:10.1371/journal.pgen.1000549)
Supplement: Table S8 — GO term enrichment among the retained genes (Fisher's exact tests). (0.06 MB PDF) [file pgen.1000549.s015.pdf]

Table S8 GO term enrichment among the retained genes (Fisher's exact tests)

GO terms	Description	FDR	p-value	Duplicates	Total genes	#nonAnnot duplicates	#nonAnnot all genes
GO:0043234	protein complex	0.004912	4.30E-05	160	691	422	2759
GO:0043232	intracellular non-membrane-bound organelle	0.008036	1.66E-04	107	435	475	3015
GO:0006091	generation of precursor metabolites and energy	0.004912	5.15E-05	54	170	528	3280
GO:0009057	macromolecule catabolic process	0.004433	6.93E-06	43	111	539	3339
GO:0044265	cellular macromolecule catabolic process	0.004550	1.66E-05	39	100	543	3350
GO:0030163	protein catabolic process	0.004550	2.86E-05	26	54	556	3396
GO:0006511	ubiquitin-dependent protein catabolic process	0.004912	5.90E-05	22	43	560	3407
GO:0051603	proteolysis involved in cellular protein catabolic process	0.005906	7.68E-05	22	44	560	3406
GO:0046365	monosaccharide catabolic process	0.008036	1.27E-04	15	23	567	3427
GO:0016717	oxidoreductase activity	0.004912	5.43E-05	6	1	576	3449
GO:0009260	ribonucleotide biosynthetic process	0.008036	1.38E-04	19	36	563	3414
GO:0006006	glucose metabolic process	0.004550	2.11E-05	20	33	562	3417
GO:0031012	extracellular matrix	0.013976	9.79E-04	7	6	575	3444
