# Supplementary material for: Genomic Analysis of the Basal Lineage Fungus Rhizopus oryzae Reveals a Whole-Genome Duplication
Source: PLoS Genet. 2009 Jul 3;5(7):e1000549. doi: 10.1371/journal.pgen.1000549 (PMC2699053; doi:10.1371/journal.pgen.1000549)
Supplement: Table S9 — Duplication of oxidative phosphorylation protein complexes. (0.12 MB PDF) [file pgen.1000549.s016.pdf]

Table S9. Duplication of respiratory chain related protein complexes *

COMPLEX I (NADH-Ubiquinone Oxidoreductase)

Reference Gene +	Size/ Annotation	Gene Symbol	Rhizopus Homolog	Protein similarity (%)	E-Value	Blast score
NCU05008.3	9.6kD	acp-1	RO3G_16841.1	66.25	5.00E-23	101
			RO3G_07961.1	65.00	6.00E-23	101
			RO3G_12225.1	65.00	2.00E-22	99.8
NCU09002.3	10.6kD	nuo10.6	RO3G_14943.1	54.72	3.00E-14	71.6
			RO3G_14590.1	57.69	4.00E-14	71.2
NCU03093.3	12.3kD	nuo12.3	RO3G_14168.1	50.00	8.00E-10	57
			RO3G_02491.1	48.00	3.00E-09	55.1
NCU02534.3	49kD	nuo49	RO3G_07618.1	77.06	0	636
			RO3G_06555.1	77.06	0	636
NCU02472.3	20.8kD	nuo20.8	RO3G_06908.1	60.63	7.00E-41	161
			RO3G_05071.1	58.27	4.00E-39	155
NCU01765.3	78kD	nuo78	RO3G_12339.1	62.54	0	875
			RO3G_13384.1	61.67	0	867
NCU05009.3	21.3c kD	nuo21.3c	RO3G_10997.1	80.00	1.00E-75	277
			RO3G_05089.1	80.00	1.00E-75	277
NCU01859.3	20.9kD	nuo20.9	RO3G_15697.1	53.02	6.00E-38	151
			RO3G_16026.1	50.00	3.00E-36	146
NCU00418.3	14.8kD	nuo14.8	RO3G_04569.1	43.10	3.00E-26	112
			RO3G_02063.1	43.22	5.00E-26	111
NCU00484.3	18.4kD	nuo18.4	RO3G_08515.1	50.00	3.00E-29	122
			RO3G_11801.1	46.75	1.00E-15	77.8
NCU08930.3	21.3kD	nuo21.3a	RO3G_13586.1	38.27	8.00E-09	55.5
			RO3G_07408.1	38.27	5.00E-08	52.8
NCU11348.3			RO3G_07804.1	61.90	1.00E-21	96.3
			RO3G_07930.1	61.67	5.00E-21	94.4
NCU09299.3	14kD	nuo14	RO3G_08458.1	42.86	3.00E-19	88.6
			RO3G_00378.1	44.54	4.00E-19	88.2
NCU09460.3	20.1kD	nuo20.1	RO3G_14313.1	42.31	2.00E-15	77.4
			RO3G_02334.1	39.22	6.00E-15	75.5
NCU05221.3	21kD	nuo21	RO3G_13870.1	47.14	4.00E-33	136
			RO3G_13157.1	49.59	4.00E-30	126
NCU05299.3	29.9kD	nuo29.9	RO3G_06118.1	50.00	2.00E-09	105
			RO3G_15124.1	52.50	2.00E-08	101
NCU01169.3	24kD	nuo24	RO3G_09707.1	62.70	6.00E-83	302
			RO3G_03903.1	61.89	6.00E-83	302
NCU01142.3	13.4kD	nuo13.4	RO3G_04950.1	39.81	3.00E-17	82
			RO3G_11038.1	32.94	9.00E-10	57
NCU04044.3	chain F 1	nuo51	RO3G_00533.1	74.89	0	712
			RO3G_16215.1	74.34	0	708
NCU02280.3	21.3kD	nuo21.3b	RO3G_07616.1	31.19	9.00E-08	52
			RO3G_06554.1	31.19	9.00E-08	52
NCU04074.3	30.4kD	nuo30.4	RO3G_06497.1	62.38	4.00E-81	296

NCU00670.3	9.5kD	nuo9.5	RO3G_14398.1	42.31	2.00E-07	49.3
NCU03156.3	10.5kD	nuo10.5	RO3G_06650.1	46.43	7.00E-17	80.5
NCU02373.3	40kD	nuo40	RO3G_11657.1	45.30	2.00E-74	274
NCU03953.3	19.3kD	nuo19.3	RO3G_13183.1	85.81	3.00E-70	259
NCU01360.3	11.5kD	nuo11.5	RO3G_11564.1	39.29	1.00E-11	62.8
NCU00160.3	6.6kD	nuo6.6	RO3G_05662.1	35.31	3.84E-4	38.5
NCU04753.3	11.6kD	nuo11.6	RO3G_01216.1	31.36	2.67E-5	42.4

COMPLEX II (Succinate Dehydrogenase-CoQ Oxoreductase):

Reference Gene +	Gene Symbol	Rhizopus Homolog	Protein similarity (%)	E-value	Blast score
YKL148C	SDH1	RO3G_07288.1	77.18	0	921
		RO3G_13321.1	75.65	0	920
YLL041C	SDH2	RO3G_03816.1	76.07	6.00E-109	388
		RO3G_04752.1	73.09	2.00E-97	350
YKL141W	SDH3	RO3G_03148.1	50	2.92E-21	97
YDR178W	SDH4	RO3G_05559.1	50	1.13E-13	72
		RO3G_02004.1	53	4.43E-10	60

COMPLEX III (Cytochrome Reductase)

Reference Gene +	Annotation	Gene Symbol	Rhizopus Homolog	Protein similarity (%)	E-Value	Blast score
YBL045C	core	COR1	RO3G_01811.1	36.08	4.00E-68	254
			RO3G_00800.1	36.26	3.00E-66	248
YEL024W	Rieske	RIP1	RO3G_11123.1	66.85	7.00E-75	275
			RO3G_01417.1	67.93	7.00E-75	275
			RO3G_13836.1	66.85	3.00E-74	273
YOR065W	CYC1	CYT1	RO3G_00332.1	62.1	8.00E-103	368
			RO3G_08415.1	70.98	1.00E-102	367
YPR191W		QCR2	RO3G_04355.1	27.94	2.00E-23	105
			RO3G_03819.1	28.06	1.00E-13	72.4
			RO3G_09327.1	27.09	1.00E-12	69.3
			RO3G_04336.1	26.64	8.00E-12	66.6
YFR033C		QCR6	RO3G_13436.1	40.68	4.00E-06	45.8
			RO3G_07357.1	40.68	4.00E-06	45.8
YDR529C		QCR7	RO3G_03897.1	47.41	9.00E-23	100
			RO3G_09716.1	41.38	2.00E-16	79.7
YGR183C		QCR9	RO3G_15029.1	51.02	1.00E-10	60.1
			RO3G_06079.1	51.02	1.00E-10	59.7
YJL166W		QCR8	RO3G_11468.1	49.41	2.00E-20	92.8
			RO3G_02864.1	49.41	2.00E-20	92.8
YHR001W-A		QCR10	RO3G_02864.1	23.22	0.879	27.2

COMPLEX IV (CYTOCHROME OXIDASE)

Reference Gene	Size/ Annotation	Gene Symbol	Rhizopus Homolog	Protein similarity (%)	E-Value	Blast score
YGL187C	sub IV	COX4	RO3G_04949.1	51.33	4.00E-29	122
			RO3G_11037.1	50.44	1.00E-28	120
YNL052W	sub Va	COX5A	RO3G_11057.1	37.04	2.00E-23	103
			RO3G_05140.1	39.34	8.00E-23	101
YIL111W	sub Vb	COX5B	RO3G_11057.1	38.97	2.00E-19	90.1
			RO3G_05140.1	40.65	5.00E-19	88.6
YHR051W	sub VI	COX6	RO3G_15433.1	67.89	3.00E-36	145
			RO3G_10189.1	67.92	2.00E-35	143
YLR038C	sub Via	COX12	RO3G_08272.1	66.67	8.00E-23	100
			RO3G_00018.1	56.76	7.00E-23	100
YGL191W	sub Vib	COX13	RO3G_14410.1	36.9	3.00E-12	65.5
			RO3G_09053.1	40.48	2.00E-12	66.6
YDL067C	sub VII	COX9	RO3G_01074.1	35.19	2.00E-06	45.8
YMR256C	sub VIIa	COX7	RO3G_10165.1	28.11	0.0778	30.8
			RO3G_15364.1	28.23	0.1734	29.6
YLR395C	sub VIII	COX8	RO3G_13126.1	34.48	0.0025	35.8
			RO3G_07144.1	34.48	0.0055	34.6

COMPLEX V (ATP SYNTHASE)

Reference Gene	Size/ Annotation	Gene Symbol	Rhizopus Homolog	Protein similarity (%)	E-Value	Blast score
YBL099W	F1Alpha	ATP1	RO3G_05483.1	78.67	0	731
			RO3G_17086.1	78.47	0	728
			RO3G_00738.1	78.47	0	728
YJR121W	F1Beta	ATP2	RO3G_01285.1	77.34	0	743
			RO3G_08262.1	77.91	0	741
			RO3G_16396.1	77.91	0	740
YBR039W	StalkGamma	ATP3	RO3G_00424.1	44.64	3.00E-59	223
			RO3G_06170.1	45	9.00E-59	222
YDL004W	StalkDelta	ATP16	RO3G_16397.1	56.31	3.00E-28	119
			RO3G_08261.1	54.37	5.00E-28	118
YDR298C	OSCP	ATP5	RO3G_14729.1	39.57	4.00E-34	139
			RO3G_09398.1	40.11	4.00E-34	139
YPL078C	Subunitb	ATP4	RO3G_13208.1	39.9	1.00E-47	184
			RO3G_12242.1	39.5	2.00E-47	184
			RO3G_17073.1	37.6	9.00E-45	175
YKL016C	Subunitd	ATP7	RO3G_01495.1	44.2	1.00E-39	157
			RO3G_11396.1	44.2	3.00E-39	155
YDR377W	Subunitf	ATP17	RO3G_09471.1	48.0	7.00E-24	103
			RO3G_01118.1	46.1	8.00E-23	100

YNL315C	Chaperone		RO3G_04994.1	31.43	2.00E-29	124
YPR155C	Regulator	NCA2	RO3G_11375.1	35.5	1.00E-29	126

* The homologous genes were detected using BLASTP (1e-5) and additional manual checking for short proteins that have low e-value and missed from initial search.

+ Nuclear proteins from SGD are used to search for the homologous sequence in *R. oryzae* protein set, except for the Proteins of complex I, the NADH-ubiquinone oxidoreductase, of *Neurospora crassa* were used for the searches, since *Saccharomyces cerevisiae* does not have this protein complex.
