# Supplementary material for: Genomic Analysis of the Basal Lineage Fungus Rhizopus oryzae Reveals a Whole-Genome Duplication
Source: PLoS Genet. 2009 Jul 3;5(7):e1000549. doi: 10.1371/journal.pgen.1000549 (PMC2699053; doi:10.1371/journal.pgen.1000549)
Supplement: Table S10 — Duplication of V-ATPase. (0.07 MB PDF) [file pgen.1000549.s017.pdf]

Table S10 Duplication of V-ATPase*

Subunits	SC_gene_id	Subunit	SC_Gene Symbol	Rhizopus Homolog	Protein similarity (%)	E-Value	Blast score
	YDL185W	SubunitA	TFP1	RO3G_03665.1	75.49	2.00E-111	842
				RO3G_01898.1	61.8	3.00E-124	884
	YBR127C	SubunitB	VMA2	RO3G_09588.1	84.23	0	819
				RO3G_12684.1	88.59	0	673
	YKL080W	SubunitC	VMA5	RO3G_04912.1	40.47	6.00E-69	256
				RO3G_16944.1	37.33	1.00E-66	249
V_1	YEL051W	SubunitD	VMA8	RO3G_08247.1	63.95	3.00E-81	296
				RO3G_16433.1	64.34	9.00E-81	295
	YOR332W	SubunitE	VMA4	RO3G_17130.1	35.38	8.00E-35	142
				RO3G_15731.1	38.98	3.00E-29	123
	YGR020C	SubunitF	VMA7	RO3G_03185.1	60.17	3.00E-33	135
	YPR036W	SubunitH	VMA13	RO3G_02983.1	31.08	1.00E-28	123
	YHR039C-A	SubunitG	VMA10	RO3G_05370.1	36.28	3E-12	65.5
	YEL027W	c	VMA3	RO3G_06137.1	72.9	5.00E-44	171
				RO3G_00069.1	72.34	2.00E-36	146
V_0	YPL234C	c'	VMA11	RO3G_15091.1	69.03	3.00E-50	192
				RO3G_08000.1	69.28	1.00E-49	190
	YHR026W	C''	VMA16	RO3G_10009.1	61.58	3.00E-43	169
				RO3G_06330.1	46	2.00E-26	114
	YMR054W	a	STV1	RO3G_04819.1	41.41	2.00E-172	602
	YLR447C	d	VMA6	RO3G_07464.1	51.20	5E-94	343

* The homologous genes were detected using BLASTP (1e-5). Proteins from SGD are used to search for the homologous sequence in *R. oryzae* protein set.
