# Supplementary material for: Genomic Analysis of the Basal Lineage Fungus Rhizopus oryzae Reveals a Whole-Genome Duplication
Source: PLoS Genet. 2009 Jul 3;5(7):e1000549. doi: 10.1371/journal.pgen.1000549 (PMC2699053; doi:10.1371/journal.pgen.1000549)
Supplement: Table S11 — Duplication of ubiquitin-proteosome system. (0.08 MB PDF) [file pgen.1000549.s018.pdf]

Table S11 Duplication of ubiquitin-proteosome system *

Domain	SC_gene_id	Subunit	SC_Gene Symbol	Rhizopus Homolog	Protein similarity (%)	E-Value	Blast score
	YMR314W	alpha1	PRE5	RO3G_07009.1	51.06	1.00E-61	231
				RO3G_05229.1	46.39	7.00E-39	155
	YML092C	alpha2	PRE8	RO3G_00510.1	63.16	4.00E-83	302
	YOR362C	alpha3	PRE10	RO3G_02194.1	56.10	5.00E-71	263
				RO3G_17174.1	58.50	6.00E-60	226
ALPHA	YGR135W	alpha4	PRE9	RO3G_16334.1	62.81	9.00E-86	311
GO:0019773				RO3G_01442.1	62.40	6.00E-85	308
	YGR253C	alpha5	PUP2	RO3G_13946.1	72.29	4.00E-92	332
				RO3G_02363.1	71.43	1.00E-91	330
	YGL011C	alpha6	SCL1	RO3G_08543.1	53.5	3.00E-66	246
				RO3G_11840.1	49.25	4.00E-63	236
	YOL038W	alpha7	PRE6	RO3G_10410.1	63.98	7.00E-85	308
				RO3G_12882.1	63.14	1.00E-83	304
	YBL041W	beta1	PRE7	RO3G_02562.1	48.71	2.00E-60	227
				RO3G_12422.1	48.71	3.00E-60	226
	YER012W	beta2	PRE1	RO3G_02025.1	46.63	3.00E-51	196
				RO3G_00621.1	50.00	7.00E-48	185
	YER094C	beta3	PUP3	RO3G_16220.1	67.84	5.00E-72	265
				RO3G_00525.1	60.00	5.00E-54	205
BETA	YFR050C	beta4	PRE4	RO3G_17028.1	46.72	3.00E-64	239
GO:0019774				RO3G_05772.1	46.72	1.00E-63	238
	YPR103W	beta5	PRE2	RO3G_02563.1	72.73	8.00E-99	355
				RO3G_12421.1	75.69	2.00E-78	287
	YJL001W	beta6	PRE3	RO3G_17089.1	69.39	7.00E-78	285
				RO3G_05850.1	69.90	7.00E-78	285
	YOR157C	beta7	PUP1	RO3G_00008.1	61.26	8.00E-85	308
	YKL145W	ATPase	RPT1	RO3G_11342.1	74.73	0	649
				RO3G_01562.1	74.09	0	644
	YDL007W	ATPase	RPT2	RO3G_10320.1	77.57	8.00E-168	585
				RO3G_09510.1	77.57	8.00E-168	585
	YDR394W	ATPase	RPT3	RO3G_02649.1	78.51	8.00E-152	531
				RO3G_11703.1	78.24	1.00E-151	531

ATPase GO:0008540	YOR259C	ATPase	RPT4	RO3G_05070.1	72.05	1.00E-166	581
				RO3G_17184.1	72.05	4.00E-166	579
	YOR117W	ATPase	RPT5	RO3G_03248.1	73.48	2.00E-156	547
	YGL048C	ATPase	RPT6	RO3G_12232.1	78.72	4.00E-179	622
				RO3G_13191.1	74.87	2.00E-166	580
LID GO:0008541	YHR027C	lid	RPN1	RO3G_04180.1	46.59	4.00E-141	497
				RO3G_05728.1	47.02	8.00E-141	496
	YIL075C	lid	RPN2	RO3G_03530.1	44.97	0.00	739
				RO3G_13173.1	44.60	0.00	729
	YER021W	lid	RPN3	RO3G_08878.1	35.14	3.00E-81	297
				RO3G_13792.1	44.69	1.00E-60	229
	YDL147W	lid	RPN5	RO3G_11498.1	41.34	3.00E-89	324
	YDL097C	lid	RPN6	RO3G_03191.1	52.76	2.00E-98	354
				RO3G_10647.1	48.57	6.00E-62	233
	YPR108W	lid	RPN7	RO3G_03190.1	42.15	2.00E-80	294
				RO3G_10648.1	38.22	2.00E-65	245
	YOR261C	lid	RPN8	RO3G_05875.1	57.19	6.00E-85	309
				RO3G_04162.1	55.67	9.00E-79	288
	YDR427W	lid	RPN9	RO3G_14739.1	38.97	1.00E-62	235
				RO3G_09387.1	36.5	1.00E-60	229
	YHR200W	lid	RPN10	RO3G_03333.1	52.54	8.00E-61	228
	YFR004W	lid	RPN11	RO3G_06585.1	66.24	2.00E-108	387
				RO3G_07479.1	68.66	2.00E-99	357
	YFR052W	lid	RPN12	RO3G_11697.1	35.27	4.00E-21	97.1
	YLR421C	lid	RPN13	RO3G_15490.1	41.59	9.47-05	35.01
				RO3G_03740.1	37.00	0.00233	32.23
Ubi-Modi-fiers	YGR048W	E1	UFD1	RO3G_14097.1	41.56	1.00E-57	218
				RO3G_02537.1	40.77	7.00E-40	159
	YBR170C	E2	NPL4	RO3G_06628.1	40.48	5.00E-113	403
	YDL126C	E3	CDC48	RO3G_12602.1	75.48	0	1089
				RO3G_07895.1	74.94	0	1088

* The homologous genes were detected using BLASTP (1e-5); the homologous sequence for a short protein YLR421C (156 aa) was identified through manual inspection.
