# Supplementary material for: Genomic Analysis of the Basal Lineage Fungus Rhizopus oryzae Reveals a Whole-Genome Duplication
Source: PLoS Genet. 2009 Jul 3;5(7):e1000549. doi: 10.1371/journal.pgen.1000549 (PMC2699053; doi:10.1371/journal.pgen.1000549)
Supplement: Table S12 — Retained genes in both R. oryzae and S. cerevisiae. (0.09 MB PDF) [file pgen.1000549.s019.pdf]

Table S12. Retained genes in both *R. oryzae* and *S. cerevisiea**.

R. oryzae gene1	*R. oryzae* gene2	Yeast gene1	Yeast gene2	Slim GO Annotation
RO3G_07249	RO3G_13520	YBR010W	YNL031C	DNA binding
RO3G_08219	RO3G_16444	YBR009C	YNL030W	DNA binding
RO3G_10354	RO3G_15990	YIL131C	YNL068C	DNA binding
RO3G_12371	RO3G_12626	YDR099W	YER177W	DNA binding protein binding
RO3G_00320	RO3G_08446	YMR194W	YPL249C-A	RNA binding
RO3G_00782	RO3G_09466	YHL001W	YKL006W	RNA binding
RO3G_00938	RO3G_09443	YGR034W	YLR344W	RNA binding
RO3G_08705	RO3G_11747	YFR023W	YHR015W	RNA binding
RO3G_12214	RO3G_13373	YDL224C	YNL197C	RNA binding
RO3G_04904	RO3G_16953	YKL043W	YMR016C	transcription regulator activity
RO3G_05555	RO3G_14665	YDR146C	YLR131C	transcription regulator activity
RO3G_10056	RO3G_13100	YDR096W	YER169W	transcription regulator activity
RO3G_12555	RO3G_14007	YIR033W	YKL020C	transcription regulator activity
RO3G_10401	RO3G_12891	YCR093W	YMR164C	transcription regulator activity hydrolase activity
RO3G_10199	RO3G_15453	YHR206W	YJR147W	transcription regulator activity signal transducer activity
RO3G_02645	RO3G_11701	YGL049C	YGR162W	translation regulator activity
RO3G_04020	RO3G_15740	YJL138C	YKR059W	translation regulator activity
RO3G_05654	RO3G_17064	YLR249W	YNL014W	translation regulator activity
RO3G_09650	RO3G_15791	YDR385W	YOR133W	translation regulator activity
RO3G_10028	RO3G_13087	YEL034W	YJR047C	translation regulator activity protein binding
RO3G_01176	RO3G_05593	YCR091W	YNR047W	protein kinase activity
RO3G_01176	RO3G_05593	YJL164C	YKL166C	protein kinase activity
RO3G_01176	RO3G_05593	YKL126W	YMR104C	protein kinase activity
RO3G_04287	RO3G_04878	YCL024W	YDR507C	protein kinase activity
RO3G_04287	RO3G_04878	YDR122W	YLR096W	protein kinase activity
RO3G_04287	RO3G_04878	YFR014C	YOL016C	protein kinase activity
RO3G_04287	RO3G_04878	YOR233W	YPL141C	protein kinase activity
RO3G_04287	RO3G_04878	YAL017W	YOL045W	protein kinase activity
RO3G_04827	RO3G_06755	YHR135C	YNL154C	protein kinase activity
RO3G_08009	RO3G_15101	YDR490C	YOL100W	protein kinase activity
RO3G_08009	RO3G_15101	YNL298W	YOL113W	protein kinase activity
RO3G_08009	RO3G_15101	YOR231W	YPL140C	protein kinase activity
RO3G_08009	RO3G_15101	YCR073C	YNR031C	protein kinase activity signal transducer activity
RO3G_00880	RO3G_05293	YDL138W	YDL194W	signal transducer activity
RO3G_00951	RO3G_07161	YGR070W	YLR371W	signal transducer activity
RO3G_04824	RO3G_06759	YOR256C	YPL176C	signal transducer activity
RO3G_08587	RO3G_11831	YDR379W	YOR127W	signal transducer activity
RO3G_08936	RO3G_14483	YDL134C	YDL188C	phosphoprotein phosphatase activity
RO3G_08936	RO3G_14483	YDR436W	YML016C	phosphoprotein phosphatase activity
RO3G_08936	RO3G_14483	YLR433C	YML057W	phosphoprotein phosphatase activity
RO3G_00320	RO3G_08446	YMR192W	YPL249C	enzyme regulator activity
RO3G_02731	RO3G_11489	YEL022W	YJR031C	enzyme regulator activity
RO3G_03537	RO3G_13177	YNL293W	YOL112W	enzyme regulator activity
RO3G_15392	RO3G_16585	YLL016W	YLR310C	enzyme regulator activity
RO3G_01343	RO3G_16166	YOR204W	YPL119C	helicase activity

RO3G_00670 #	RO3G_05373	YJL134W	YKR053C	hydrolase activity
RO3G_00819	RO3G_01991	YLR300W	YOR190W	hydrolase activity
RO3G_02809	RO3G_11650	YLR293C	YOR185C	hydrolase activity
RO3G_04015	RO3G_15739	YBR001C	YDR001C	hydrolase activity
RO3G_06245	RO3G_15996	YER031C	YGL210W	hydrolase activity
RO3G_06248	RO3G_15998	YNL098C	YOR101W	hydrolase activity
RO3G_08555	RO3G_11836	YNL093W	YOR089C	hydrolase activity
RO3G_12040	RO3G_16321	YCR069W	YNR028W	isomerase activity
RO3G_12040	RO3G_16321	YDR304C	YHR057C	isomerase activity
RO3G_04903#	RO3G_16954	YKL132C	YMR113W	ligase activity
RO3G_10443	RO3G_12701	YMR246W	YOR317W	ligase activity
RO3G_15613	RO3G_15934	YGR124W	YPR145W	ligase activity
RO3G_00315	RO3G_08439	YAL029C	YOR326W	motor activity
RO3G_00315	RO3G_08439	YKL129C	YMR109W	motor activity
RO3G_00787	RO3G_09469	YDL085W	YMR145C	oxidoreductase activity
RO3G_01672	RO3G_04863	YGR256W	YHR183W	oxidoreductase activity
RO3G_05136	RO3G_11062	YDR368W	YOR120W	oxidoreductase activity
RO3G_07288	RO3G_13321	YJL045W	YKL148C	oxidoreductase activity
RO3G_08033	RO3G_15197	YGR192C	YJR009C	oxidoreductase activity
RO3G_02462	RO3G_12289	YDR453C	YML028W	oxidoreductase activity
RO3G_00045 #	RO3G_06063	YEL060C	YOR003W	peptidase activity
RO3G_00534	RO3G_16214	YHR047C	YKL157W	peptidase activity
RO3G_00042	RO3G_06065	YDR252W	YPL037C	protein binding
RO3G_04076	RO3G_09793	YIL109C	YNL049C	protein binding
RO3G_10403	RO3G_12888	YBL075C	YER103W	protein binding
RO3G_10403	RO3G_12888	YDL229W	YNL209W	protein binding
RO3G_11230	RO3G_13636	YMR186W	YPL240C	protein binding
RO3G_00270	RO3G_08363	YER131W	YGL189C	structural molecule activity
RO3G_04000	RO3G_10491	YNL096C	YOR096W	structural molecule activity
RO3G_04451	RO3G_11162	YML085C	YML124C	structural molecule activity
RO3G_05063	RO3G_17194	YDL061C	YLR388W	structural molecule activity
RO3G_06221	RO3G_16080	YGL076C	YPL198W	structural molecule activity
RO3G_08513	RO3G_11799	YGR214W	YLR048W	structural molecule activity
RO3G_01687	RO3G_04988	YBL068W	YER099C	transferase activity
RO3G_02914	RO3G_11596	YGR043C	YLR354C	transferase activity
RO3G_04008	RO3G_15735	YCL040W	YDR516C	transferase activity
RO3G_04008	RO3G_15735	YFR053C	YGL253W	transferase activity
RO3G_04284	RO3G_04876	YCR034W	YJL196C	transferase activity
RO3G_08020	RO3G_11887	YAL038W	YOR347C	transferase activity
RO3G_08940	RO3G_14478	YDL131W	YDL182W	transferase activity
RO3G_10063	RO3G_13112	YFR015C	YLR258W	transferase activity
RO3G_00880	RO3G_05293	YDR497C	YOL103W	transporter activity
RO3G_02737	RO3G_11496	YCR037C	YJL198W	transporter activity
RO3G_09643	RO3G_15800	YCL025C	YDR508C	transporter activity
RO3G_09643	RO3G_15800	YEL063C	YNL270C	transporter activity
RO3G_10435	RO3G_12710	YBR104W	YPR058W	transporter activity
RO3G_10435	RO3G_12710	YEL006W	YIL006W	transporter activity
RO3G_10435	RO3G_12710	YOR222W	YPL134C	transporter activity
RO3G_00883	RO3G_05289	YDR185C	YLR168C	

RO3G_05087	RO3G_11000	YBR214W	YGL056C
RO3G_08257	RO3G_16403	YHR001W	YKR003W
RO3G_08257	RO3G_16403	YOR237W	YPL145C
RO3G_08527	RO3G_11806	YBR082C	YDR059C
RO3G_08585	RO3G_11870	YBR284W	YJL070C
RO3G_09020	RO3G_16999	YIL149C	YKR095W
RO3G_10355#	RO3G_15987	YMR237W	YOR299W
RO3G_10403	RO3G_12888	YBR169C	YPL106C
RO3G_11231	RO3G_13634	YFL004W	YPL019C
RO3G_12215	RO3G_13372	YBR241C	YGL104C

* The homologous gene pairs were defined based on BLASTP (1e-5). The functional annotation of the genes was based on yeast gene ontology (GO)-slim function annotations downloaded from SGD (http://downloads.yeastgenome.org/literature_curation/go_slim_mapping.tab).
# *R. oryzae* duplicates with substantially different branch length presented in the unrooted tree as illustrated in Figure 3 (from the root to the tip, one branch is at least 50% longer than the other).
