# Supplementary material for: Genomic Analysis of the Basal Lineage Fungus Rhizopus oryzae Reveals a Whole-Genome Duplication
Source: PLoS Genet. 2009 Jul 3;5(7):e1000549. doi: 10.1371/journal.pgen.1000549 (PMC2699053; doi:10.1371/journal.pgen.1000549)
Supplement: Table S13 — Comparison of P-loop GTPases in R. oryzae and dikaryotic fungi. (0.21 MB PDF) [file pgen.1000549.s020.pdf]

Table S13. Comparison of P-loop GTPases in *R. oryzae* and dikaryotic fungi.

Name(s) of orthologs	No. of paralogs in Rhizopus	Gene ID	Assigned function	Phyletic pattern of orthologs in Dikarya								Notes
				Ncr	Afu	Yli	Sce	Spo	Uma	Cne	Cci	
Ras1	5	RO3G_07499.1<br>RO3G_13463.1*<br>RO3G_01916.1<br>RO3G_04548.1<br>Missing annotation on supercontig 3.2	Acts downstream of heterotrimeric G-protein signalling to activate adenylate cyclase, involved in conidial germination and cell proliferation	2	2	2	2	1	1	2	1	*S. cerevisiae* Ras2 also belongs here
Ras2 (as in *U. maydis*)	2	RO3G_05138.1<br>RO3G_11060.1*	Regulation of cell morphogenesis and hyphal growth	1	1	1	0	0	1	1	3	Not to be confused with *S. cerevisiae* Ras2
Rsr1	1	RO3G_01917.1	In fungi involved regulation of cell polarity, hyphal growth, and bud site selection	1	1	1	1	0	1	0	1	Rap in Metazoa
Rhb1	2	RO3G_00157.1*<br>RO3G_09734.1	Regulation of arginine uptake, cell growth and cell cycle progression	1	1	1	1	1	1	1	3	Rheb in Metazoa
Ral	1	RO3G_01344.1	Metazoan Ral involved in diverse signalling pathways regulating vesicle trafficking, neurite branching, or tumorigenesis	0	0	0	0	0	0	0	0	Absent from all genomes of Dikarya in GenBank/EMBL/DDBJ. RalA and RalB in human
Rho1	6	RO3G_02066.1<br>RO3G_04572.1<br>RO3G_13357.1<br>RO3G_15190.1<br>RO3G_02938.1<br>RO3G_11556.1	Regulates establishment of cell polarity, branching, and cell wall synthesis etc.	1	1	2	1	2	1	1	≥2	
Rho2	0	-	Regulates cell polarity, cell wall synthesis and microtubule assembly	1	1	1	1	1	1	1	1	Present in all Dikarya investigated as well as *Phycomyces blakesleeanus*, hence the absence from *Rhizopus* is suspicious
Rho3	2	RO3G_13515.1*<br>RO3G_02725.1*	Involved in exocytosis, cell polarisation, integrity and separation	1	1	1	1	1	1	1	1	
Rho4	2	RO3G_09420.1*<br>RO3G_15544.1*	Involved in cytokinesis, cell integrity, actin polarisation	1	1	1	1	1	1	1	1	
Rac1 (as in *U. maydis*)	3	RO3G_02338.1<br>RO3G_14316.1<br>RO3G_11983.1*	Involved in cell morphogenesis and hyphal growth	1	1	1	1	0	1	2	1	In *S. cerevisiae* called Rho5
Cdc42	2	RO3G_08857.1<br>RO3G_15801.1*	Directs cell polarisation, separation, and fusion	1	1	1	1	1	1	2	1	
Gem1	2	RO3G_13437.1*<br>RO3G_07349.1*	Associated with the outer mitochondrial membrane, regulates mitochondrial transport and morphology	1	1	1	1	1	1	1	1	In Metazoa called Miro
Ypt1	3	RO3G_00820.1*<br>RO3G_01990.1*<br>RO3G_12274.1	Involved in vesicle transport between the ER and Golgi	1	1	1	1	1	1	1	1	Rab1 in Metaoza
Rab2	3	RO3G_04163.1*<br>RO3G_11441.1*<br>RO3G_02789.1*	Metazoan orthologs involved in trafficking to and within Golgi	1	1	1	0	0	1	1	1	
Ypt4 (as in *S. pombe*)	1	Missing annotation on supercontig 3.12	Metazoan orthologs involved in endosomal recycling	1	1	1	0	1	1	1	1	Rab4 in Metazoa

Ypt51	4	RO3G_08555.1<br>RO3G_11836.1<br>RO3G_13349.1*<br>RO3G_14664.1	Involved in endosomal trafficking and sorting	1	1	1	2	0	1	1	1	Conserved fungal paralog of the RAB5 subfamily, Ypt51 and Ypt53 in *S. cerevisiae*
Ypt52	3	RO3G_08452.1<br>RO3G_14437.1*<br>RO3G_00179.1	Involved in endosomal trafficking and sorting	1	1	1	2	1	1	1	1	Conserved fungal paralog of the RAB5 subfamily, Ypt52 and Ypt10 in *S. cerevisiae*
Ypt6	6	RO3G_02207.1*<br>RO3G_04138.1*<br>RO3G_08514.1*<br>RO3G_14572.1*<br>RO3G_04837.1*<br>RO3G_06909.1*	Endosome-Golgi and intra-Golgi retrograde transport	1	1	1	1	1	1	1	1	Rab6 in Metazoa
Ypt7	4	RO3G_11650.1<br>RO3G_03032.1*<br>RO3G_02809.1<br>Missing annotation on supercontig 3.9	Involved in trafficking to the vacuole and homotypic vacuole fusion	1	1	1	1	2	2	1	3	Rab7 in Metazoa
Sec4	2	RO3G_06248.1*<br>RO3G_15998.1*	Regulates post-Golgi transport of secretory vesicles	1	1	1	1	1	1	1	1	
Ypt3 (as in *S.pombe*)	10<br>(some may be pseudogenes)	RO3G_06245.1<br>RO3G_15996.1<br>RO3G_01792.1<br>RO3G_08324.1*<br>RO3G_01249.1<br>RO3G_03332.1*<br>RO3G_16667.1*<br>RO3G_16638.1*<br>RO3G_16671.1*<br>RO3G_16674.1*	TGN-plasma membrane and/or endosome-plasma membrane transport	1	1	2	2	1	1	1	1	Ypt31 and Ypt32 in *S. cerevisiae*, Rab11 in Metazoa
Rab18	1	RO3G_02866.1	In Metazoa involved in ER-Golgi trafficking, associates with lipid droplets	1	0	0	0	0	1	0	1	
Rab32 (as in human)	1	RO3G_11660.1	In Metazoa involved in trafficking to lysosome-derived organelles (e.g. melanosomes)	0	0	0	0	0	0	0	0	Absent from all genomes of Dikarya in GenBank/EMBL/DDBJ. Orthologs: Rab32 and Rab38 in human, Glo-1 in *C. elegans*, Lightoid in *D. melanogaster*
Un-named RAB	2	RO3G_08045.1*<br>RO3G_15234.1*	Uncharacterised RAB GTPase	1	1	1	0	0	1	1	1	May be orthologous to *Drosophila* chrowded gene product
Tem1	1	RO3G_02554.1*	Regulates progression through the cell cycle	1	1	1	1	1	1	1	1	Spg1 in *S. pombe*
RAN (as in human)	2	RO3G_16626.1<br>RO3G_15528.1	Governs transport from and into the nucleus and spindle assembly	1	2	1	2	1	2	1	1	Gsp1 and Gsp2 in *S. cerevisiae*
Arf1/Arf2	3	RO3G_11156.1<br>RO3G_04456.1<br>RO3G_13794.1*	Regulate vesicle budding at the Golgi, recruit COP-I coat subunits	1	1	1	2	1	1	1	1	
Arf3	2	RO3G_16324.1*<br>Missing annotation on supercontig 3.2	Involved in endocytosis, cortical actin organisation, and polarised cell growth	1	1	1	1	1	1	1	1	
Arl1	1<br>(+ pseudogene)	RO3G_08018.1*<br>(RO3G_11890.1)	Golgi-associated protein, recruits GRIP domain protein and the GARP tethering complex to the Golgi	1	1	1	1	0	1	1	1	
Arl2 (as in human)	1	Missing annotation on supercontig 3.26	Regulates assembly of tubulin alpha/beta dimers	1	1	1	1	1	1	1	1	Cin4 in *S. cerevisiae*

Arl5	1	RO3G_07539.1*	In Metazoa associates with the Golgi (function unknown)	0	0	0	0	0	1	1	0	Absent from all genomes of Ascomycota in GenBank/EMBL/DDBJ
Arl8	2	RO3G_12397.1<br>RO3G_09848.1	In metazoa associates with lysosomes and regulates their transport	1	1	1	0	0	1	1	1	
ARFRP1 (as in human)	1	RO3G_10847.1*	Golgi-associated GTPase required for targeting of Arl1 GTPase to the Golgi	1	1	1	1	0	1	1	1	In *S. cerevisiae* confusingly called Arl3, which is used as a name for a different ARF-like GTPase conserved in eukaryotes (but lost from fungi)
Un-named ARF-like protein	1	Missing annotation on supercontig 3.1	Uncharacterised protein with an ARF-like GTPase domain and a novel C-terminal domain	0	1	0	0	0	0	0	1	Extremely limited phylogenetic distribution, beyond some Fungi found so far only in green algae *Chlamydomonas* and *Volvox*
Sar1	2	RO3G_05258.1<br>RO3G_00846.1	Recruits the COPII coat to the ER leading to vesicle budding	1	1	1	1	1	1	1	1	
Srp102	1	RO3G_00650.1*	Beta subunit of the signal recognition particle receptor	1	1	1	1	1	1	1	1	
Gpa1	5	RO3G_00005.1*<br>RO3G_00875.1<br>RO3G_01120.1<br>RO3G_09475.1<br>RO3G_06003.1*	Alpha subunit of heteromeric G-proteins	1	1	1	1	1	2	1	≥14	
Gpa2	2	RO3G_15639.1*<br>RO3G_06081.1*	Alpha subunit of heteromeric G-proteins	1	1	1	1	1	1	1	1	*Ustilago maydis* Gpa2 is in fact orthologous to Gna2
Gna2 (as in *Neurospora crassa*)	5	RO3G_00858.1*<br>RO3G_05285.1*<br>RO3G_15513.1*<br>RO3G_16598.1*<br>Missing annotation on supercontig 3.6	Alpha subunit of heteromeric G-proteins	1	1	0	0	0	1	1	1	
PARF/RBEL1 (as in human)	1	RO3G_00162.1*	Function unknown, human PARF (=RBEL1) interacts with the tumour-suppressor protein p14ARF, localises to the nucleus, and is overexpressed in breast cancer	0	0	0	0	0	0	0	0	Absent from all genomes of Dikarya in GenBank/EMBL/DDBJ
Gtr1	2	RO3G_03979.1*<br>RO3G_08768.1*	Subunit of the GSE complex involved in protein sorting in the endosomes, also regulates the function of the RAN GTPase	1	1	1	1	1	1	1	1	RagA and RagB in human
Gtr2	2	RO3G_12792.1*<br>RO3G_17066.1*	Subunit of the GSE complex involved in protein sorting in the endosomes, also regulates the function of the RAN GTPase	1	1	1	1	1	1	1	1	RagC and RagD in human
Dyn3	3	RO3G_07054.1*<br>RO3G_03898.1*<br>RO3G_09715.1*	Cytoplasmic dynein 1 light intermediate chain	1	1	1	1	1	1	1	1	
Tef1/Tef2	5<br>(all five encoded proteins identical)	RO3G_15351.1<br>RO3G_15047.1<br>RO3G_11153.1<br>RO3G_10090.1<br>RO3G_08488.1	Translation elongation factor EF-1α	1	1	1	2	2	1	1	1	
Tuf1	2	RO3G_01455.1<br>RO3G_16340.1*	Mitochondrial translation elongation factor EF-Tu	1	1	1	1	1	1	1	1	
Eft1/Eft2	4	RO3G_15791.1<br>RO3G_12127.1	Translation elongation factor EF-2	1	1	2	2	1	1	1	1	

		RO3G_09650.1<br>RO3G_08804.1										
Mef1	1	RO3G_07529.1	Mitochondrial translation elongation factor EF-G	1	1	1	1	1	1	1	1	
Mef2	1	RO3G_00845.1	Mitochondrial translation elongation factor EF-G	1	1	1	1	1	1	0	1	
Ria1	1	RO3G_01368.1	Cytoplasmic GTPase involved in biogenesis of the 60S ribosome	1	1	1	1	1	1	1	1	
Snu114	2	RO3G_05538.1<br>RO3G_04368.1	GTPase component of U5 snRNP involved in mRNA splicing	1	1	1	1	1	1	1	1	
Sup35	1	RO3G_13821.1	Translation termination factor eRF3	1	1	1	1	1	1	1	1	
Hbs1	1	RO3G_01063.1	GTPase regulating mRNA 'no-go decay'	1	1	1	2	2	1	1	1	
Fun12	1	RO3G_03429.1+<br>RO3G_03428.1<br>(gap in the genome sequence)	Eukaryotic translation initiation factor 5B	1	1	1	1	1	1	1	1	
Imf1	1	RO3G_12713.1	Mitochondrial translation initiation factor 2 (IF2)	1	1	1	1	1	1	1	1	
Gcd11	2	RO3G_17196.1<br>RO3G_05065.1	Gamma subunit of the translation initiation factor eIF2 (eIF2γ)	1	1	1	1	1	1	1	1	
GTPBP1 (as in human)	2	RO3G_06881.1*<br>RO3G_12386.1	Conserved GTPase with uncharacterised function	2	2	0	0	0	1	1	1	
Guf1	1	RO3G_09970.1	Mitochondrial translation elongation factor EF4 (LepA)	1	1	1	1	1	1	1	1	
BipA (as in *Escherichia coli*)	0	-	Translational factor widespread in eubacteria and rarely in eukaryotes (probably in mitochondria)	0	0	0	0	0	0	0	1	Absent from *Phycomyces blakesleeanus* and all genomes of Ascomycota in GenBank/EMBL/DDBJ but probably ancestrally present in Fungi (also in *Laccaria bicolor* and *Batrachochytrium dendrobatidis*) and lost on multiple occasions.
Bms1	1	RO3G_16413.1+<br>RO3G_16412.1<br>(gap in the genome sequence)	Nucleolar GTPase required for synthesis of the 40S ribosomal subunit and for processing of the 35S pre-rRNA	1	1	1	1	1	1	1	1	
Nog1	2	RO3G_10603.1<br>RO3G_06710.1	Nucleolar GTPase required for biogenesis of the 60 S ribosomal subunit	1	1	1	1	1	1	1	1	
Rgb1	1	RO3G_15244.1	Ribosome-interacting GTPase	1	1	1	1	1	1	1	1	
Rgb2	1	RO3G_10163.1	Conserved GTPase with uncharacterised function	1	1	1	1	1	1	1	1	
Mtg2	1	RO3G_09024.1	Mitochondrial GTPase essential for mitochondrial ribosome function	1	1	1	1	1	1	1	1	
OLA1 (as in human)	2	RO3G_07275.1<br>RO3G_11460.1	Conserved nucleolar or mitochondrial GTPase-like ATPase with uncharacterised function	1	1	2	2	2	1	1	1	Ylf2 and Ola1 in *S. cerevisiae*
Ygr210c	0	-	Uncharacterised putative GTPase, orthologs in Archaebacteria and sporadically in eukaryotes	1	1	1	1	1	0	0	0	Absent also from *Phycomyces blakesleeanus* and from all genomes of Basidiomycota checked, but present in *Batrachochytrium dendrobatidis*
Mss1	1	RO3G_09583.1	Mitochondrial GTPase involved in tRNA modification	1	1	1	1	1	1	1	1	
Ydr336w	1 (+1 pseudogene)	RO3G_09584.1	Possibly mitochondrial GTPase orthologous to	1	1	1	1	0	0	0	1	GTPBP8 in human

		(RO3G_11867.1)	prokaryotic YihA/EngB, ucharacterised in eukaryotes									
Era (as in *E. coli*)	1	RO3G_11108.1*	Possibly mitochondrial ortholog of the prokaryotic GTPase Era, which is involved in biogenesis of the small ribosome subunit	0	0	0	0	0	0	0	0	Absent from all genomes of Dikarya in GenBank/EMBL/DDBJ. ERAL1 in human
Cdc3	3	RO3G_13362.1<br>RO3G_11935.1<br>RO3G_11008.1	Septin GTPase involved in cell division	1	1	1	1	2	1	1	1	
Cdc10	4	RO3G_06303.1<br>RO3G_01893.1<br>RO3G_09681.1<br>RO3G_12834.1	Septin GTPase involved in cell division	1	1	1	1	1	1	1	1	
Cdc11	4	RO3G_13560.1<br>RO3G_05531.1<br>RO3G_14571.1<br>RO3G_07653.1	Septin GTPase involved in cell division (or sporulation)	1	1	3	3	2	1	1	2	Cdc11, Shs1, and Spr28 in *S. cerevisiae*
Cdc12	9	RO3G_07960.1<br>RO3G_14828.1<br>RO3G_04262.1<br>RO3G_16839.1<br>RO3G_10890.1<br>RO3G_09759.1<br>RO3G_06049.1<br>RO3G_00024.1<br>RO3G_01868.1	Septin GTPase involved in cell division (or sporulation)	1	1	2	2	2	1	1	1	Cdc12 and Spr3 in *S. cerevisiae*
AspE (as in *Aspergillus nidulans*)	5	RO3G_15353.1<br>RO3G_10088.1<br>RO3G_03700.1<br>RO3G_16251.1	Uncharacterised septin GTPase	1	1	0	0	0	0	1	2	
Un-named septin	1	RO3G_12633.1	?	0	0	0	0	0	0	0	0	Without obvious orthologs in Dikarya or Metazoa
Un-named GTPase-related protein	0	-	Family of uncharacterised GTPase-related proteins present sporadically throughout eukaryotes (e.g., EAL65477.1 in *Dictyostelium discoideum*)	2	0	0	0	0	0	0	0	Absent also from *Phycomyces blakesleeanus*. Orthologs in some Pezizomycotina, *Laccaria bicolor*, *Entamoeba*, *Dictyostelium*, *Giardia*, *Tetrahymena*, *Physcomitrella*, *Tribolium*
IMAP/AIG1/GtpA	0	-	Family of GTPase-related proteins with a role in anti-pathogen defence	2	0	0	0	0	0	0	>30	Absent also from *Phycomyces blakesleeanus*. Orthologs in Metazoa (IMAP family), plants (AIG1 family), *Dictyostelium* (GtpA), other ascomycetes and basidiomycetes
Nug1	1	RO3G_06983.1	Nuclear GTPase involved in export of 60S ribosomal subunits from the nucleus	1	1	1	1	1	2	2	2	
Nog2	1	RO3G_06651.1	Nucleolar GTPase required for maturation and export of pre-60S ribosomal subunits	1	1	1	1	1	1	1	1	
Mtg1	1	RO3G_12778.1	Mitochondrial GTPase involved in assembly of the large ribosomal subunit	1	1	1	1	1	1	1	1	
Lsg1	1	RO3G_09764.1	Cytoplasmic GTPase involved in 60S ribosomal subunit biogenesis	1	1	1	1	1	1	1	1	
GNL1 (as in human)	1	RO3G_13237.1	Uncharacterised conserved putative GTPase	0	0	0	0	0	1	1	1	Absent from all genomes of Ascomycota in GenBank/EMBL/DDBJ
Lrc5	1	RO3G_02308.1*	GTPase-like mitochondrial protein, ortholog	1	1	1	1	1	0	0	0	Absent from all genomes of

			from *Bacillus* (YqeH) required for ribosome assembly									Basidiomycota checked.
Vps1	2	RO3G_04899.1<br>RO3G_03500.1	Dynamin-related GTPase involved in vacuolar protein sorting and peroxisome biogenesis	1	1	2	1	1	1	1	1	
Dnm1	3	RO3G_05805.1<br>RO3G_04350.1<br>RO3G_09330.1*<br>(inaccurate genome sequence)	Cytoplasmic dynamin-related GTPase required for mitochondrial fission and the maintenance of mitochondrial morphology	1	1	1	1	1	1	1	1	
Mgm1	1	RO3G_16217.1	Mitochondrial intermembrane-space dynamin-related GTPase required for inner-membrane fusion and maintenance of cristae	1	1	1	1	1	1	1	1	
Fzo1	2	RO3G_16929.1<br>RO3G_04896.1	Mitochondrial outer membrane-integral dynamin-related GTPase involved in outer membrane fusion	1	1	1	1	1	1	1	1	
Mx	0	-	Dynamin-like proteins, in human implicated in myxovirus resistance	5	6	0	0	0	0	0	2	Present also in human and *Batrachochytirum dendrobatidis*
RME1 (as in *C. elegans*)	2 (+ 1 pseudogene)	RO3G_11210.1<br>RO3G_10389.1<br>(RO3G_12901.1)	GTPase-like ATPase and EH-domains containing protein involved in endosomal recycling	0	0	0	0	0	0	0	0	Absent from all genomes of Dikarya in GenBank/EMBL/DDBJ. EHD1-4 in human.
IRG (p47)	3	RO3G_02383.1*<br>RO3G_14116.1*<br>RO3G_06746.1*	Related to metazoan IRG (p47) interferon-inducible GTPases involved in resistance against intracellular pathogens	0	0	0	0	0	0	0	0	May have orthologs also in some Ascomycota (*Phaeosphaeria nodorum* and *Nectria haematococca*) but absent from all genomes of Basidiomycota checked.
Sey1	2	RO3G_10315.1<br>RO3G_01015.1	GTPase homologous to the Arabidopsis RHD3 implicated in ER to Golgi transport	1	1	1	1	1	1	1	1	
Srp54	1	RO3G_13370.1	Subunit of the Signal recognition particle (SRP)	1	1	1	1	1	1	1	1	
Srp101	1	RO3G_10756.1	Alpha subunit of the SRP receptor	1	1	1	1	1	1	1	1	

The sequences were identified by BLASP and PSI-BLAST searches of the database of predicted *Rhizopus* proteins (Assembly 3 annotation) and the nr database at NCBI (the remaining fungi). For *Rhizopus*, the genome was further checked by TBLASTN to identify possible homologs that had been annotated incorrectly or not at all. For the remaining fungal genomes analysed, TBLASTN searches were conducted only in cases of suspicious absence of a homolog in the protein database (leading in several cases to identification of an un-annotated gene), hence it is possible that some additional un-annotated genes have been missed. Assignment of mutual orthologs is based mainly on reciprocal BLAST comparisons and in a few cases should be verified by phylogenetic analysis (accession numbers of individual GTPases from Dikarya are available upon request). Unless stated otherwise, the names assigned to groups of orthologs follow the nomenclature of the corresponding *S. cerevisiae* genes. Probable function of individual GTPases in *Rhizopus* is assigned by consulting the literature dealing with characterisation of orthologs in other species. Green background indicates GTPases that seem to have been lost in the ancestor of Basidiomycota (based on their absence in the annotated proteomes of *Ustilago maydis*, *Cryptococcus neoformans*, and *Coprinopsis cinerea* available in GenBank/EMBL/DDBJ, as well as in *Phanerochaete chrysosporium*, *Laccaria bicolor*, and *Sporobolomyces roseus* downloaded from DOE JGI, http://genome.jgi-psf.org/euk_home.html). Violet background marks GTPases that appear to have been lost from the ancestor of Ascomycota (based on their absence from annotated proteomes of all Ascomycota in GenBank/EMBL/DDBJ as of 5/2/2007). Yellow background indicates GTPases probably lost in the ancestor of Dikarya and orange background indicates GTPases probably secondarily absent from *Rhizopus oryzae*. Gene IDs marked with an asterisk represent gene models with erroneously predicted exon/intron structure. Species abbreviation of Dikarya fungi: Ncr – *Neurospora crassa*, Afu – *Aspergillus fumigatus*, Yli – *Yarrowia lipolytica*, Sce – *Saccharomyces cerevisiae*, Spo – *Schizosaccharomyces pombe*, Uma - *Ustilago maydis*, Cne - *Cryptococcus neoformans*, Cci - *Coprinopsis cinerea*.
