# Supplementary material for: Genomic Analysis of the Basal Lineage Fungus Rhizopus oryzae Reveals a Whole-Genome Duplication
Source: PLoS Genet. 2009 Jul 3;5(7):e1000549. doi: 10.1371/journal.pgen.1000549 (PMC2699053; doi:10.1371/journal.pgen.1000549)
Supplement: Table S14 — Regulators of Ras superfamily GTPases in R. oryzae and dikaryotic fungi. (0.21 MB PDF) [file pgen.1000549.s021.pdf]

Table S14. Comparison of regulators of Ras superfamily GTPases in *Rhizopus oryzae* and Dikarya.

	Name(s) of orthologs	No. of paralogs in Rhizopus	Gene ID	Phyletic pattern of orthologs in Dikarya								Notes
				Ncr	Afu	Yli	Sce	Spo	Uma	Cne	Cci	
**RasGAP** domain proteins	Ira1/Ira2	8	RO3G_15507.1<br>RO3G_16654.1<br>RO3G_03157.1<br>RO3G_00611.1<br>RO3G_02179.1<br>RO3G_10923.1<br>RO3G_02034.1<br>RO3G_16652.1*+RO3G_16653.1*	1	0	1	2	0	1	1	1	NF1 in Metazoa
	Bud2	1	RO3G_00771.1*	1	1	1	1	0	1	0	1	Rap-specific GAP
	Iqg1	3	RO3G_05681.1<br>RO3G_10660.1<br>RO3G_05758.1	1	1	1	1	1	1	1	1	Related to IQGAP in Metazoa
	Gap1 (as in *S. pombe*)	4	RO3G_00167.1<br>RO3G_08138.1<br>RO3G_13371.1<br>RO3G_12218.1	1	1	0	0	1	1	1	1	Related to IQGAP in Metazoa
**RapGAP** domain proteins	TSC2 (as in human)	1	RO3G_03221.1	1	1	1	0	2	1	1	1	tuberin (tuberous sclerosis complex 2), GAP for the Rheb GTPase
	GARNL1/AS250 (as in human)	1	RO3G_15088.1*	0	0	0	0	0	0	0	0	Orthologs in Metazoa, absent from all genomes of Dikarya in GenBank/EMBL/DDBJ
	Rap1GAP/SIPA1/ GARNL3/GARNL4 etc. (as in human)	4	RO3G_12030.1*<br>RO3G_08783.1*<br>RO3G_04769.1*<br>RO3G_10959.1*	0	0	0	0	0	0	0	0	Orthologous to an independently diversified group of Metazoan RapGAP proteins. Absent from all genomes of Dikarya in GenBank/EMBL/DDBJ
**RasGEF** (Cdc25) domain proteins	Lte1	4	RO3G_12749.1*<br>RO3G_10474.1*<br>RO3G_00547.1*<br>RO3G_00890.1*	1	1	1	1	0	1	0	1	
	Bem2	2	RO3G_03905.1*<br>RO3G_09710.1*+RO3G_09709.1*	0	0	1	1	0	1	0	1	Has also a RhoGAP domain, see below. *A. fumigatus* Bem2 does not have the RasGEF domain, so it is not listed here
	Cdc25	8	RO3G_15392.1*<br>RO3G_16585.1*	1	1	1	1	2	1	1	≥4	In the reference *S. cerevisiae* strain S288C there is a second

			RO3G_13506.1*<br>RO3G_07261.1*<br>RO3G_00029.1*<br>RO3G_02361.1*<br>RO3G_16404.1*<br>RO3G_13944.1*<br>(inaccurate genome sequence)									paralog, pseudogene *SCD25*
	Sql2 (as in *U. maydis*)	1	RO3G_15046.1*	1	1	1	0	0	1	1	1	Ras2-specific GEF
	Un-named RasGEF domain protein	3	RO3G_00511.1*<br>RO3G_05450.1*<br>RO3G_09700.1*	1	1	0	0	0	1	1	1	
	Un-named RasGEF domain protein	2	RO3G_15992.1<br>RO3G_02886.1*	0	0	0	0	0	0	0	0	Orthologs in *Phycomyces blakesleeanus* and *Batrachochytrium dendrobatidis*. Absent from all genomes of Dikarya in GenBank/EMBL/DDBJ
	Un-named RasGEF domain protein	3	RO3G_04278.1<br>RO3G_04871.1*<br>RO3G_05302.1*	0	0	0	0	0	0	0	0	Orthologs in *Phycomyces blakesleeanus* and *Batrachochytrium dendrobatidis*. Absent from all genomes of Dikarya in GenBank/EMBL/DDBJ
	Un-named RasGEF domain protein	7	RO3G_06875.1<br>RO3G_04552.1*<br>RO3G_01921.1*<br>RO3G_13472.1*<br>RO3G_07467.1*<br>RO3G_09459.1*<br>RO3G_00769.1*	0	0	0	0	0	0	0	0	Related to Ral-specific GEFs from Metazoa. Absent from all genomes of Dikarya in GenBank/EMBL/DDBJ
**RhoGAP domain** proteins	Rga1/Rga2	5	RO3G_11831.1<br>RO3G_14386.1<br>RO3G_08587.1<br>RO3G_03394.1<br>RO3G_11177.1	1	1	1	2	1	1	1	1	
	Bem2	2	RO3G_03905.1<br>RO3G_09710.1*+RO3G_09709.1*	0	1	1	1	0	1	0	1	Has also a RasGEF domain (save *A. fumigatus*), see above
	Rgd1	2	RO3G_12149.1<br>RO3G_03176.1+ RO3G_03175.1	1	1	1	1	1	1	1	1	
	Lrg1	5	RO3G_10602.1<br>RO3G_11732.1<br>RO3G_02903.1<br>RO3G_16632.1	1	1	1	1	1	1	1	1	

			RO3G_05843.1									
	Bem3	2	RO3G_01659.1<br>RO3G_06509.1*+RO3G_06510.1*	1	2	2	1	1	1	1	1	
	Bag7/Sac7	3	RO3G_13168.1<br>RO3G_13009.1<br>RO3G_05294.1	3	1	1	2	1	1	1	1	
	Ecm25	1	RO3G_07553.1	0	0	1	1	0	1	0	1	
	KIAA1688 (as in human)	3	RO3G_04185.1<br>RO3G_15953.1<br>RO3G_16336.1	0	0	0	0	0	0	1	1	Absent from all genomes of Ascomycota in GenBank/EMBL/DDBJ
	Rgd2	4	RO3G_05223.1<br>RO3G_07014.1<br>RO3G_04920.1<br>RO3G_16939.1	1	1	2	2	2	1	1	1	
	ORCL (as in human)	1	RO3G_01102.1	1	1	1	0	1	0	1	2	
	Un-named RhoGAP protein	4	RO3G_13089.1<br>RO3G_10031.1<br>RO3G_10123.1<br>RO3G_13558.1	0	0	0	0	0	1	1	1	Absent from all genomes of Ascomycota in GenBank/EMBL/DDBJ
	Un-named RhoGAP protein	3	RO3G_08289.1<br>RO3G_04282.1<br>RO3G_02658.1	0	0	0	0	0	0	0	0	Without obvious orthologs in Dikarya or Metazoa, may be divergent paralog of Bem3
	Un-named RhoGAP protein	2	RO3G_15796.1<br>RO3G_09647.1*+ RO3G_09646.1*	0	0	0	0	0	0	0	0	Without obvious orthologs in Dikarya or Metazoa, may be divergent paralog of Bem3
**RhoGEF (DH) domain** proteins	Cdc24	5	RO3G_07086.1<br>RO3G_06839.1<br>RO3G_05390.1<br>RO3G_00602.1<br>RO3G_01873.1	1	1	1	1	1	1	1	1	
	Fus2	2	RO3G_10077.1<br>RO3G_10027.1	1	1	1	1	2	1	1	1	
	Intersectin (common name in Metazoa)	1	RO3G_00164.1	0	0	0	0	0	1	1	1	ITSN1 and ITSN2 in human. Pan1 from Ascomycota is homologous to the N-terminal half of Intersectin, but does not have the C-terminal RhoGEF domain
	Rom1/Tus1 group	9	RO3G_00951.1<br>RO3G_07161.1<br>RO3G_10407.1<br>RO3G_03084.1<br>RO3G_08775.1<br>RO3G_02600.1	2	2	2	3	3	2	3	4	Two groups of similar paralogs (Rom1/Rom2 and Tus1 in *S. cerevisiae*) both with the CNH domain conserved in Dikarya. It is unclear whether this split applies also to Rhizopus.

			RO3G_14312.1<br>RO3G_16337.1<br>RO3G_04648.1									
	Bud3	3	RO3G_09353.1<br>RO3G_02916.1<br>missing annotation on supercontig 3.9	1	1	1	1	1	1	1	1	
	Rsp (as in *Neurospora crassa*)	4	RO3G_03682.1<br>RO3G_16277.1<br>RO3G_02064.<br>RO3G_04570.1	1	1	1	0	0	1	1	1	
	GEF1 (as in *Cryptococcus neoformans*)	2	RO3G_00761.1<br>RO3G_07178.1	0	0	0	0	0	1	1	1	Absent from all genomes of Ascomycota in GenBank/EMBL/DDBJ
	Un-named RhoGEF domain protein	3	RO3G_01901.1<br>RO3G_06573.1<br>RO3G_07625.1	0	0	0	0	0	0	0	0	Orthologs difficult to define without a phylogenetic analysis
	Un-named RhoGEF domain protein	1	RO3G_05847.1	0	0	0	0	0	0	0	0	Orthologs difficult to define without a phylogenetic analysis
	Un-named RhoGEF domain protein	7	RO3G_09360.1<br>RO3G_12714.1<br>RO3G_02904.1<br>RO3G_04521.1<br>RO3G_06993.1<br>RO3G_11151.1*+ RO3G_11150.1*<br>RO3G_04760.1*+ RO3G_04761.1*	0	0	0	0	0	0	0	0	Orthologs difficult to define without a phylogenetic analysis
**CZH** family (GEFs for RHO GTPases)	Ylr422w	2	RO3G_08501.1<br>RO3G_00053.1	1	1	1	1	0	1	1	1	
**RHO GDI**	Rdi1	2	RO3G_14141.1<br>RO3G_02285.1	1	1	2	1	1	1	1	1	GDP-dissociation inhibitor of RHO GTPases
**REP/GDI** family	Gdi	2	RO3G_03976.1<br>RO3G_05580.1	1	1	1	1	1	1	1	1	GDP-dissociation inhibitor of RAB GTPases
	Mrs6	1	RO3G_16113.1	1	1	1	1	1	1	1	1	RAB escort protein (REP)
**TRAPP complex** GEF for RAB GTPases Ypt1/Rab1 (TRAPP-I) and Ypt3/Rab11 (TRAPP-II)	Bet3	1	RO3G_16065.1*+RO3G_16066.1*	1	1	1	1	1	1	1	1	Both TRAPP-I and TRAPP-II
	Bet5	1	RO3G_11159.1	1	1	1	1	1	1	1	1	Both TRAPP-I and TRAPP-II
	Trs20	1	Missing annotation on supercontig 3.2	1	1	1	1	1	1	1	1	Both TRAPP-I and TRAPP-II
	Trs23	1	RO3G_00822.1	1	1	1	1	1	1	1	1	Both TRAPP-I and TRAPP-II
	Trs31	1	RO3G_01760.1	1	1	1	1	1	1	1	1	Both TRAPP-I and TRAPP-II
	Trs33	1 (+ 1 pseudogene)	RO3G_12899.1<br>(RO3G_10390.1)	1	1	1	1	1	1	1	1	Both TRAPP-I and TRAPP-II
	Gsg1	1	RO3G_12377.1	1	1	1	1	2	1	1	1	Both TRAPP-I and TRAPP-II

	Trs120	2	RO3G_05389.1<br>RO3G_09669.1	1	1	1	1	1	1	1	1	Only in TRAPP-II
	Trs130	1	RO3G_08432.1	1	1	1	1	1	1	1	1	Only in TRAPP-II
	Kre11	1	RO3G_11258.1	1	1	1	1	0	1	1	1	Only in TRAPP-II
**VPS9 domain** proteins (RAB5 subfamily GEFs)	Vps9	2	RO3G_05970.1*<br>RO3G_10327.1*	1	1	1	1	1	1	1	1	
	Muk1	1	RO3G_16880.1*	1	1	1	1	1	1	1	1	
	Yml002w	2	RO3G_06004.1*<br>RO3G_00004.1*	1	1	1	1	0	0	0	0	Absent from all genomes of Basidiomycota checked
**Sec2 -like** GEF for the Sec4 GTPase	Sec2	8	RO3G_11194.1*<br>RO3G_04377.1*<br>RO3G_08373.1*<br>RO3G_16280.1<br>RO3G_03691.1*<br>RO3G_12699.1*<br>RO3G_12892.1*<br>RO3G_10400.1*	2	2	1	1	1	1	1	2	
**Ric1-Rgp1 complex** GEF for the Ypt6 GTPase	Ric1	1	RO3G_14200.1*	1	1	1	1	1	1	1	1	
	Rgp1	1	RO3G_06973.1*+ RO3G_06972.1*	1	1	1	1	1	1	1	1	
**Vam6** GEF for the Ypt7 GTPase	Vam6	1	RO3G_02435.1	1	1	1	1	1	1	1	1	Subunit of the HOPS tethering complex
**RCC1 domain** proteins (included only those directly talking to Ras superfamily GTPases)	Smr1	4	RO3G_05651.1<br>RO3G_17067.1<br>RO3G_16655.1<br>Missing annotation on supercontig 3.6	2	1	1	1	1	1	1	1	RCC1 in Metazoa, GEF for the RAN GTPase
	Un-named RCC1 domain protein	1	RO3G_15049.1	0	0	0	0	0	0	0	0	RCC1 domain protein most similar to Glo-4 from *C. elegans*, GEF for the RAB GTPase Glo-1 (=Rab32). Orthologs absent from all genomes of Dikarya in GenBank/EMBL/DDBJ
	RCC2 (as in human)	0	-	0	0	0	0	0	1	1	1	Present also in Metazoa and *Phycomyces blakesleeanus*, absent from all genomes of Ascomycota in GenBank/EMBL/DDBJ. Potential GEF for the Rac GTPase
**TBC domain** proteins (GAPs for RAB GTPases)	Gyp1	1	RO3G_00588.1*	1	1	1	1	1	1	1	1	
	Mdr1	3	RO3G_09061.1<br>RO3G_07207.1<br>RO3G_00751.1	1	1	1	1	2	1	1	1	

	Msb3/Msb4	3	RO3G_03537.1*<br>RO3G_13177.1*<br>Missing annotation on supercontig 3.8	1	1	1	2	1	0	0	0	Absent from all genomes of Basidiomycota checked
	Gyp5/Gyl1	13	RO3G_05966.1<br>RO3G_10323.1<br>RO3G_09504.1<br>RO3G_00639.1<br>RO3G_00320.1<br>RO3G_04495.1<br>RO3G_14812.1<br>RO3G_08446.1<br>RO3G_14017.1<br>RO3G_11763.1<br>RO3G_08672.1<br>RO3G_06612.1<br>RO3G_07454.1	2	2	2	2	2	1	1	1	
	Gyp6	1	RO3G_14233.1	1	1	1	1	0	1	1	1	
	Gyp7	3	RO3G_05764.1<br>RO3G_17026.1<br>RO3G_05445.1	1	1	1	1	1	1	1	1	
	Gyp8	1	RO3G_12284.1	1	1	1	1	1	1	1	1	
	USP6 (as in human)	1	RO3G_06304.1	1	1	0	0	1	1	1	1	
	TBC1D2 (as in human)	0	-	1	1	1	0	1	1	1	1	Absent also from *Phycomyces blakesleeanus* but conserved in Metazoa
	TBC1D10 (as in human)	1	RO3G_03397.1	0	0	0	0	0	1	1	1	Absent from all genomes of Ascomycota in GenBank/EMBL/DDBJ
	TBC1D13 (as in human)	2	RO3G_05305.1<br>RO3G_01069.1	0	0	0	0	1	1	1	1	
	TBC1D14 (as in human)	1	RO3G_03132.1	1	1	1	0	1	1	1	1	
	TBCKL (as in human)	1	RO3G_04794.1	0	0	0	0	0	0	0	0	Absent from all genomes of Dikarya in GenBank/EMBL/DDBJ
	Un-named TBC domain protein	1	RO3G_13954.1*	0	0	0	0	0	1	1	0	Absent from all genomes of Ascomycota in GenBank/EMBL/DDBJ
**Bub2-Bfa1** complex (GAP for the Tem1 GTPase)	Bub2	1	RO3G_01384.1*	1	1	1	1	1	1	1	1	TBC domain protein
	Bfa1	1	RO3G_03177.1	1	1	1	1	1	1	1	1	

**RanGAP**	Rna1	1	RO3G_03167.1	1	1	1	1	1	1	1	1	
**ArfGAP domain** proteins	Age2	2	RO3G_00007.1<br>RO3G_06180.1	1	1	1	1	1	1	1	1	
	Gts1	2	RO3G_12895.1<br>RO3G_08312.1	1	1	0	1	1	1	1	1	
	Age1	3	RO3G_14443.1<br>RO3G_09068.1<br>RO3G_01995.1	1	1	1	1	2	0	0	0	Absent from all genomes of Basidiomycota checked
	Glo3	1	RO3G_01480.1	1	1	1	1	1	1	1	1	
	Gsc1	1	RO3G_10897.1	1	1	1	2	1	0	1	1	
	CentB (as in human)	1	RO3G_08017.1*	0	0	0	0	0	0	0	0	Centaurin beta in Metazoa, absent from all genomes of Dikarya in GenBank/EMBL/DDBJ
**Sec7 domain** proteins (GEFs for ARF GTPases)	Sec7	1	RO3G_13039.1	1	1	1	1	2	1	1	1	
	Gea1/Gea2	2	RO3G_11489.1<br>RO3G_02731.1	1	1	1	2	1	1	1	1	
	Syt1	1	RO3G_10935.1	1	1	1	1	1	1	1	1	
	Yel1	9	RO3G_10627.1<br>RO3G_03013.1<br>RO3G_14057.1<br>RO3G_14056.1<br>RO3G_12588.1<br>RO3G_12330.1<br>RO3G_01540.1<br>RO3G_06627.1	1	1	2	1	2	1	1	1	
	Un-named Sec7 domain protein	1	RO3G_11024.1	0	0	0	0	0	1	1	1	Absent from all genomes of Ascomycota in GenBank/EMBL/DDBJ
**Sec23** (GAP for the Sar1 GTPase)	Sec23	2	RO3G_16013.1<br>RO3G_10467.1	1	1	2	1	2	1	1	1	Subunit of the COP-II coat complex
**Sec12** (GEF for the Sar1 GTPase)	Sec12	2	RO3G_09896.1<br>RO3G_08472.1	1	1	1	2	1	1	1	1	Sec12 and Sed4 in *S. cerevisiae*
**RGS** proteins (GAPs for heterotrimeric G-protein alpha subunits)	Sst2	5	RO3G_16098.1<br>RO3G_06205.1<br>RO3G_14307.1<br>RO3G_07201.1<br>RO3G_00754.1	1	1	1	1	1	1	1	1	FlbA in *Aspergillus nidulans*
	Rgs2	2	RO3G_00567.1<br>Missing annotation on supercontig 3.3	1	2	1	1	0	0	0	0	RgsA and RgsD in *Aspergillus nidulans*. Absent from all genomes of Basidiomycota

												checked
	Rax1	5	RO3G_08205.1<br>RO3G_16473.1<br>RO3G_01435.1<br>RO3G_07341.1<br>RO3G_13412.1	1	1	1	1	1	1	1	1	RgsB in *Aspergillus nidulans*
	Mdm1	1	RO3G_13123.1	1	1	1	1	1	1	1	1	RgsC in *Aspergillus nidulans*
	Un-named RGS protein	0	-	2	0	0	0	0	0	1	1	Present in *Phycomyces blakesleeanus*
	Un-named RGS protein	5	RO3G_02906.1<br>RO3G_11612.1<br>RO3G_04098.1<br>RO3G_10279.1<br>RO3G_02018.1	0	0	0	0	0	0	0	0	Orthologs in *Phycomyces blakesleeanus* and *Batrachochytrium dendrobatidis*. Absent from all genomes of Dikarya in GenBank/EMBL/DDBJ
	Un-named RGS protein	1	RO3G_05992.1	0	0	0	0	0	0	0	0	Except *Phycomyces blakesleeanus* without obvious orthologs elsewhere. Fusion of the RGS and Pyr_redox domains

Fungal sequences falling into (admittedly an inexhaustive selection of) various categories of known regulators of the Ras GTPase superfamily were identified and analysed by an approach similar to that used for the analysis of fungal GTPases (see Table S11). In addition, for identification of proteins containing poorly conserved regulatory domains, we also used HMMER searches with profile HMMs built from multiple alignments retrieved from Pfam (http://www.sanger.ac.uk/Software/Pfam/) or SMART (http://smart.embl-heidelberg.de/) collections. As for GTPases, the *Rhizopus* genome was further checked by TBLASTN for possible un-annotated homologs while the remaining fungal genomes were not analysed systematically by TBLASTN searches. The meaning of the colour coding is the same as in the Table S15. Orthology of a few *Rhizopus* Ras superfamily regulators (particularly in the RhoGEF group) to genes in other species could not be defined with confidence and a detailed phylogenetic analysis is required to determine their actual relationships. Gene IDs marked with an asterisk represent gene models with erroneously predicted exon/intron structure. Species abbreviation of Dikarya fungi: Ncr – *Neurospora crassa*, Afu – *Aspergillus fumigatus*, Yli – *Yarrowia lipolytica*, Sce – *Saccharomyces cerevisiae*, Spo – *Schizosaccharomyces pombe*, Uma - *Ustilago maydis*, Cne - *Cryptococcus neoformans*, Cci - *Coprinopsis cinerea*.
