# Supplementary material for: Genomic Analysis of the Basal Lineage Fungus Rhizopus oryzae Reveals a Whole-Genome Duplication
Source: PLoS Genet. 2009 Jul 3;5(7):e1000549. doi: 10.1371/journal.pgen.1000549 (PMC2699053; doi:10.1371/journal.pgen.1000549)
Supplement: Table S15 — Enriched proteases gene families. (0.08 MB PDF) [file pgen.1000549.s022.pdf]

Table S15 enriched proteases gene families *

Family	Name	*R. oryzae*	*C. neoformans*	*A. fumigatus*	*M. grisea*	*S. cerevisiae*	*C. albicans*
A1	secreted aspartic protease family	28	7	6	8	7	14
S8	Subtilisin family	23	2	4	7	4	2
C14	Caspase family	7	1	0	1	1	1
C26	Gamma-glutamyl hydrolase family	10	0	2	0	4	0
C44	Peptidase family C44	7	2	1	2	6	3
M24	Peptidase family M24	13	2	5	3	5	1
M28	Aminopeptidase Y family	8	3	3	6	7	0
S1	Chymotrypsin family	16	0	0	0	1	0
S9	Prolyl oligopeptidase family	25	4	3	14	3	1
S16	Lon protease family	5	0	0	0	1	1
S33	Peptidase family S33	9	1	2	18	4	0
C19	Ubiquitin-specific protease family	21	3	0	5	17	8
M67	Peptidase family M67	14	1	2	3	3	2
T1	Proteasome family	26	12	14	11	14	6

*The annotation is based on the Merops the peptidase database http://merops.sanger.ac.uk/index.htm[46]
