# Supplementary material for: Genomic Analysis of the Basal Lineage Fungus Rhizopus oryzae Reveals a Whole-Genome Duplication
Source: PLoS Genet. 2009 Jul 3;5(7):e1000549. doi: 10.1371/journal.pgen.1000549 (PMC2699053; doi:10.1371/journal.pgen.1000549)
Supplement: Table S16 — Annotation of cell wall synthesis enzymes and secreted proteases. (0.06 MB PDF) [file pgen.1000549.s023.pdf]

Table S16 Annotation of cell wall synthesis enzymes and secreted proteases

CHS	CDA	SAP	Subtilase
RO3G_00426	RO3G_01408	RO3G_01229*	RO3G_00045
RO3G_00522	RO3G_01485*	RO3G_01232*	RO3G_00079
RO3G_00942	RO3G_01608	RO3G_01236*	RO3G_02461*
RO3G_02895	RO3G_01628*	RO3G_01282	RO3G_02880*
RO3G_03037	RO3G_02639*	RO3G_01464*	RO3G_03817#
RO3G_03188	RO3G_02640*	RO3G_01479	RO3G_04043
RO3G_04443	RO3G_03009*	RO3G_01759*	RO3G_04302
RO3G_04703	RO3G_03155*	RO3G_01762*	RO3G_05735
RO3G_06167	RO3G_03353	RO3G_02121	RO3G_05984*
RO3G_08099	RO3G_04734	RO3G_02201*	RO3G_06132*
RO3G_08861	RO3G_04738#	RO3G_04164	RO3G_10128
RO3G_10151	RO3G_04739#	RO3G_04713*	RO3G_10488*
RO3G_10568	RO3G_04740	RO3G_05810*	RO3G_11852
RO3G_11165	RO3G_04890	RO3G_07000*	RO3G_12259*
RO3G_11725	RO3G_04891	RO3G_07001	RO3G_12287*
RO3G_11900	RO3G_05839*	RO3G_08001	RO3G_14159
RO3G_12476	RO3G_06820	RO3G_08248#	RO3G_15326
RO3G_13033	RO3G_06829	RO3G_08302	RO3G_15505*
RO3G_13214	RO3G_09560#	RO3G_11353#	RO3G_16044*
RO3G_14688	RO3G_10385*	RO3G_12822*	RO3G_16116
RO3G_15485	RO3G_10624#	RO3G_13330*	RO3G_16311
RO3G_16230	RO3G_10891*	RO3G_13840*	RO3G_16312
RO3G_17187	RO3G_11105	RO3G_13967*	RO3G_16659
	RO3G_11389*	RO3G_15092*	
	RO3G_11684*	RO3G_16415	
	RO3G_11699*	RO3G_16968*	
	RO3G_12766#	RO3G_17183*	
	RO3G_12767#		
	RO3G_12908*		
	RO3G_15196		
	RO3G_15691		
	RO3G_16202#		
	RO3G_17100*		

* predicted to be secreted
# predicted to be glycosyl-phosphatidylinositol (GPI)-proteins
Yellow shaded boxes highlight the recent tandem duplicates that have opposite oridentation and share 100% nucleotide sequence identity.
