# Supplementary material for: Genomic Analysis of the Basal Lineage Fungus Rhizopus oryzae Reveals a Whole-Genome Duplication
Source: PLoS Genet. 2009 Jul 3;5(7):e1000549. doi: 10.1371/journal.pgen.1000549 (PMC2699053; doi:10.1371/journal.pgen.1000549)
Supplement: Table S17 — Ergosterol biosynthesis pathway in R. oryzae. (0.06 MB PDF) [file pgen.1000549.s024.pdf]

Table S17 Ergosterol biosynthesis pathway in *R. oryzae*

Gene symbol	SC_gene_id	*Rhizopus* Homolog	Protein similarity (%)	E-Value	Blast score
ERG1	YGR175C	RO3G_11081.1	38.56	4.00E-87	317
		RO3G_02215.1	37.09	6.00E-82	300
ERG4	YGL012W	RO3G_10689.1	47.21	6.00E-103	369
		RO3G_08866.1	44.44	3.00E-98	354
		RO3G_15495.1	48.72	2.00E-68	255
ERG5	YMR015C	RO3G_04913.1	44.93	6.00E-118	419
		RO3G_16941.1	44.93	9.00E-117	416
		RO3G_04483.1	41.56	2.00E-102	368
ERG10	YPL028W	RO3G_14944.1	51.76	6.00E-117	416
		RO3G_14592.1	52.74	1.00E-115	411
		RO3G_00814.1	50.76	7.00E-95	342
ERG3	YLR056W	RO3G_07367.1	44.24	9.00E-80	292
		RO3G_13407.1	40.28	1.00E-78	288
ERG6	YML008C	RO3G_16049.1	55.8	2.00E-104	374
		RO3G_15767.1	56.67	1.00E-102	368
ERG11	YHR007C	RO3G_11790.1	44.25	8.00E-120	426
		RO3G_16595.1	41.54	2.00E-103	371
ERG24	YNL280C	RO3G_15373.1	47.25	1.00E-94	342
		RO3G_13480.1	37.03	1.00E-72	268
ERG25	YGR060W	RO3G_00626.1	48.75	2.00E-79	291
		RO3G_07587.1	46.38	4.00E-71	263
ERG26	YGL001C	RO3G_05740.1	41.74	2.00E-65	244
		RO3G_13830.1	38.44	1.00E-57	218
ERG2	YMR202W	RO3G_06146.1	44.59	1.00E-47	184
ERG7	YHR072W	RO3G_15243.1	47.33	8.00E-169	589
ERG8	YMR220W	RO3G_14539.1	37.07	2.00E-33	139
ERG9	YHR190W	RO3G_05144.1	47.33	3.00E-95	344
ERG12	YMR208W	RO3G_00394.1	37.57	6.00E-25	110
ERG13	YML126C	RO3G_13071.1	50.88	3.00E-123	437
ERG20	YJL167W	RO3G_14682.1	61.6	2.00E-125	444
ERG27	YLR100W	RO3G_07492.1	29.65	9.00E-26	112
ERG28	YER044C	RO3G_05441.1	45.31	9.00E-31	127
HMG1	YML075C	RO3G_14184.1	41.17	0	636
HMG2	YLR450W	RO3G_04292.1	55.8	2.00E-147	519
IDI1	YPL117C	RO3G_13592.1	54.87	1.00E-69	258
		RO3G_07411.1	57.01	1.00E-67	251
MCR1	YKL150W	RO3G_08200.1	38.8	9.00E-48	185
		RO3G_06161.1	37.6	2.00E-44	174
		RO3G_05026.1	34	3.00E-37	150
		RO3G_08293.1	34.24	4.00E-37	150
		RO3G_03220.1	34.15	4.00E-35	143
MVD1	YNR043W	RO3G_11218.1	52.1	2.00E-97	351
NCP1	YHR042W	RO3G_02475.1	39.2	6.00E-116	413
		RO3G_14252.1	37.63	7.00E-116	413
