# Supplementary material for: Genomic Analysis of the Basal Lineage Fungus Rhizopus oryzae Reveals a Whole-Genome Duplication
Source: PLoS Genet. 2009 Jul 3;5(7):e1000549. doi: 10.1371/journal.pgen.1000549 (PMC2699053; doi:10.1371/journal.pgen.1000549)
Supplement: Table S18 — Fungal homologs to Metazoa. (0.07 MB PDF) [file pgen.1000549.s025.pdf]

Table S18. Fungal homologs to Metazoa

Genomes	Total Genes	Metazoa homologs	NonHomologs	p value*
Rhizopus oryzae	13,936	4,632	9,304	-
Coprinus cinereus	13,544	3,288	10,256	5.00E-60
Magnaporthe grisea	12,841	3,276	9,565	1.00E-60
Neurospora crassa	9,845	2,758	7,087	1.00E-43
Fusarium verticillioides	14,199	3,485	10,714	2.40E-62
Aspergillus nidulans	10,665	3,273	7,392	0.000008

* The *p*-values for testing that there are significantly fewer metazoan homologous genes in each of the dikaryotic fungi compared to *R. oryzae* were computed using Fisher's exact tests.
