# Supplementary material for: Genomic Analysis of the Basal Lineage Fungus Rhizopus oryzae Reveals a Whole-Genome Duplication
Source: PLoS Genet. 2009 Jul 3;5(7):e1000549. doi: 10.1371/journal.pgen.1000549 (PMC2699053; doi:10.1371/journal.pgen.1000549)
Supplement: Table S19 — Comparison of the core elements of the MEN/SIN pathway. (0.09 MB PDF) [file pgen.1000549.s026.pdf]

Table S19. Comparison of the core elements of the MEN/SIN pathway

S. cerevisiae	*S. pombe*	*A. nidulans*	*C. neoformans*	*U. maydis*	*R. oryzae*
Tem1p	Spg1	AN7206	CNAG_05513	UM04012	RO3G_02554
Bub2p	Cdc16	AN0281	CNAG_05406	UM00561	RO3G_01384
Bfa1p	Byr4	AN9413	CNAG_06325	UM02890	RO3G_03177
Cdc15p	Cdc7	SEPH	CNAG_06845	UM00721	None*
Dbf2p/SidB	Sid2	SIDB	CNAG_02194	UM03446	None

* five paralogues share only the kinase domain: RO3G_16294.1 (352 aa), RO3G_12035.1 (333 aa), RO3G_16945.1 (314 aa), RO3G_04911.1 (538 aa), RO3G_03201.1 (495 aa), which are much smaller comparing to the other proteins (Supplementary Figure S7).
