# Supplementary material for: Genomic Analysis of the Basal Lineage Fungus Rhizopus oryzae Reveals a Whole-Genome Duplication
Source: PLoS Genet. 2009 Jul 3;5(7):e1000549. doi: 10.1371/journal.pgen.1000549 (PMC2699053; doi:10.1371/journal.pgen.1000549)
Supplement: Table S20 — Growth comparison (37°C) of R. oryzae 99–880 versus A. fumigatus AF293. (0.08 MB PDF) [file pgen.1000549.s027.pdf]

Table S20 Growth comparison (37°C) of *R. oryzae* 99-880 vs *A. fumigatus* AF293

Culture medium	PDA	YPD	PDA	YPD
Organism	*R. oryzae*	*R. oryzae*	*A. fumigatus*	*A. fumigatus*
24 hours	3.43±0.11	3.67±.016	0.53±0.04	0.3±0.07
48 hours	5.13±0.11	6.2±0.07	1.7±0.07	1.3±0.07
72 hours	≥8.50*	≥8.50	3.67±0.09	3.13±0.11

* ≥ 8.5 means covered the whole plate
